# Scopararanes C–G: New Oxygenated Pimarane Diterpenes from the Marine Sediment-Derived Fungus *Eutypella scoparia *FS26

**DOI:** 10.3390/md10030539

**Published:** 2012-02-27

**Authors:** Li Sun, Dongli Li, Meihua Tao, Yuchan Chen, Feijun Dan, Weimin Zhang

**Affiliations:** 1 Guangdong Provincial Key Laboratory of Microbial Culture Collection and Application, Guangdong Provincial Open Laboratory of Applied Microbiology, State Key Laboratory of Applied Microbiology (Ministry-Guangdong Province Jointly Breeding Base), South China, Guangdong Institute of Microbiology, Guangzhou 510070, China; Email: sunli525525525@yahoo.com.cn (L.S.); yichen802003@yahoo.com.cn (D.L.); taomeihua@yahoo.com.cn (M.T.); yuchan2006@126.com (Y.C.); 2 College of Chemistry and Life Science, China Three Gorges University, Yichang 443002, China; Email: danfj0458@yahoo.com.cn

**Keywords:** pimarane diterpenes, marine sediment-derived fungus, *Eutypella scoparia*, South China Sea, cytotoxic activity

## Abstract

Five new oxygenated pimarane diterpenes, named scopararanes C–G (**1**–**5**) were isolated from the culture of a marine sediment-derived fungus *Eutypella scoparia* FS26 obtained from the South China Sea. The structures of these compounds were established on the basis of extensive spectroscopic analysis. The absolute configurations of compounds **1**–**5**, were determined by CD spectroscopic analysis and comparison with literature data. All isolated compounds (**1**–**5**) were evaluated for their cytotoxic activities against MCF-7, NCI-H460, and SF-268 tumor cell lines by the 3-(4,5-dimethylthiazol-2-yl)-2,5-diphenyl-2*H*-tetrazolium bromide (MTT) method.

## 1. Introduction

In recent years, the rich and unprecedented chemical diversity of natural products from marine-derived fungi and their pronounced biological activities continue to attract the interest of natural product chemists, ecologists and pharmacologists alike [1,2,3,4,5]. The total number of new natural products from marine-derived fungi had already exceeded 1000 by the end of the year 2010, and approximately 16% of the new natural products were isolated from sediments [6]. Recently, we isolated a fungus strain, *Eutypella scoparia* FS26 from marine sediment.

**Figure 1:**
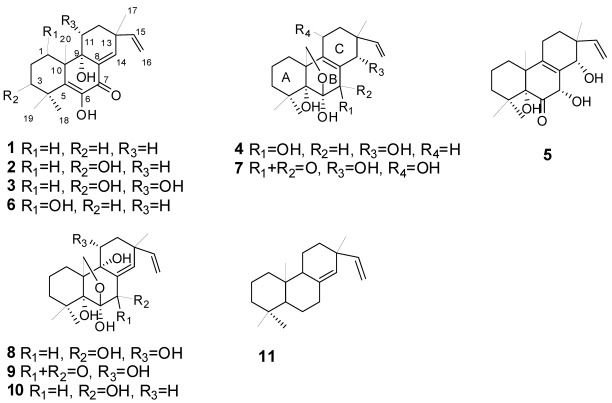
Chemical structures of compounds **1**–**11**.

*Eutypella* species are present in a diverse array of habitats, including soil in Antarctica and tropical forests of Australia and Thailand. It was also found in the marine pulmonate mollusc *Onchidium* sp. [7]. Although only few chemical investigations of the genus *Eutypella* were reported, the numerous secondary metabolites from this genus were isolated, including polyketides such as γ-lactones, benzopyran derivatives and cytosporin-related compounds, terpenoids such as *ent*-eudesmane sesquiterpenes, and pimarane diterpenes, and nitrogenous compounds such as cytochalasin derivatives and cyclic dipeptides [7,8,9,10]. Previously, we have reported eleven terpenoids, five ergosterol derivatives, and two nitrogen-containing metabolites from the culture of *E. scoparia* FS26 [11,12,13]. Among these terpenoids, there were six known pimarane diterpenes, libertellenone A (**6**), scopararane B (**7**), diaporthein A (**8**), diaporthein B (**9**), 11-deoxydiaporthein A (**10**), and isopimara-8(14),15-diene (**11**). We now report the isolation, structure elucidation and cytotoxicity of five new pimarane diterpenes, scopararanes C–G (**1**–**5**) (Figure 1). 

## 2. Results and Discussion

The fungus *E. scoparia* FS26 was isolated from a marine sediment sample from the South China Sea. It was cultured in potato dextrose broth (PDB) in 50% (v/v) sea water. The culture (100 L) was centrifuged to separate broth and mycelia, followed by extraction with ethyl acetate. The concentrated extracts were further purified by various chromatographic methods including silica gel, reversed-phase silica gel C_18_, and Sephadex LH-20 to yield scopararanes C–G (**1**–**5**).

### 2.1. Structural Elucidation

**Figure 2:**
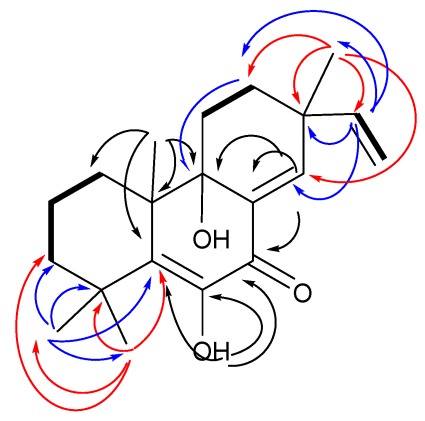
Key HMBC (arrows) and COSY (bold lines) correlations of compound **1**.

**Figure 3:**
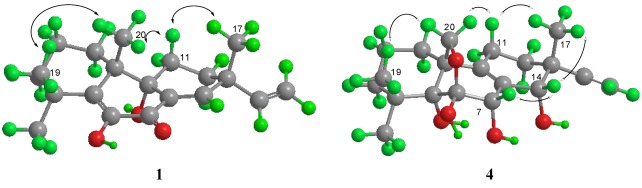
Key NOESY correlations of compounds **1** and **4**, whose conformation was modeled by using the MM2 minimum energy calculation [15].

Scopararane C (**1**) was obtained as colorless crystals. The molecular formula, C_20_H_28_O_3_, consistent with seven degrees of unsaturation, was determined by HR-EI-MS (*m/z* 316.2032, [M]^+^, C_20_H_28_O_3_^+^; calcd 316.2033). The ^1^H NMR spectrum of **1** demonstrated signals characteristic of terminal vinylic protons at [δ_H_ 5.87 (1H, dd, *J* = 17.5, 10.7 Hz, H-15), 5.10 (1H, dd, *J* = 17.5 Hz, 0.7 Hz , H-16a), and 5.06 (1H, dd, *J* = 10.7, 0.7 Hz, H-16b)] , an olefinic proton at [δ_H_ 7.04 (1H, d, *J* = 1.8 Hz, H-14)], and four tertiary methyl groups at [δ_H_ 1.41 (3H, s, H-18), 1.29 (3H, s, H-19), 1.21 (3H, s, H-20), and 1.13 (3H, s, H-17)] (Table 1; all spectra are provided as supplementary material). Analyses of ^1^H, ^13^C, DEPT135 and HSQC NMR data (Table 1) revealed that scopararane C contains 20 carbons including four singlet methyls, six methylenes (one sp^2^ and five sp^3^), two sp^2^ methines, and eight quaternary carbons (one α,β-unsaturated carbonyl, an oxygenated sp^2^, two sp^2^, an oxygenated sp^3^, and three sp^3^). The ^1^H and ^13^C NMR spectra of **1** were similar to those reported earlier for pimarane-type diterpene libertellenone A (**6**) [12,14], except the oxymethine at [δ_H_ 4.34 (dd, *J *= 9.5, 5.5 Hz, H-1); δ_C_ 69.3)] in **6** was replaced by the methylene at [δ_H_ 1.98 (m), 1.53 (m); δ_C_ 30.6)] in **1**, and the chemical shift of C-2 (δ_C_ 17.7) in **1** was upfield shifted by ∆δ 11.5 ppm compared with that of C-2 (δ_C_ 29.2) in **6** (Table 1). ^1^H,^1^H-COSY spectrum of **1** revealed the partial structure H-1 to H-3 via H-2, the connectivity between H-11 and H-12, and also the connectivity between H-15 and H-16 (Figure 2). The observed HMBC correlations from H-18 to C-3, C-4, C-5 and C-19, H-19 to C-3, C-4, C-5 and C-18, H-20 to C-1, C-5, C-9, and C-10, H-17 to C-12, C-13, C-14, and C-15, H-15 to C-12, C-13, C-14, and C-17, H-14 to C-7, C-8 and C-9, H-12 to C-9, and 6-OH to C-5, C-6, and C-7 (Figure 2), further confirmed the structure for **1**. The relative configuration of **1** was identical to that in libertellenone A (**6**) according to proton coupling constants and NOESY spectrum results (Figure 3). The coupling constants of H-11β (ddd, *J *= 12.4, 12.4, 3.2 Hz) showed the characteristics of geminal and axial-axial relationships, which indicated the pseudoaxial position of H-11β. H-11β showed NOE correlations with H-17 and H-20, and H-20 showed NOE correlation with H-19, demonstrating that C-17, C-19, and C-20 are in axial configurations on the top face of the molecule. The hydroxyl group at C-9 was established as beta-axial based upon the NOE correlation between H-11β and H-20.

**Table 1:** ^1^H and ^13^C NMR spectroscopic data of compounds **1**–**3**.

Position	1 ^a^		2 ^a^		3 ^b^	
	δ_C__, _mult.	δ_H_ ( *J *in Hz)	δ_C__, _mult.	δ_H_ ( *J *in Hz)	δ_C__, _mult.	δ_H_ ( *J *in Hz)
1α	30.6, CH_2_	1.98, m	28.8, CH_2_	2.12, ddd (13.0, 13.0, 4.1)	32.0, CH_2_	2.18, ddd (13.5, 13.5, 4.7)
1β		1.53, m		1.59, m		1.85, ddd (13.5, 4.2, 1.6)
2	17.7, CH_2_	1.65, 2m	25.7, CH_2_	1.78, 2m	26.7, CH_2_	1.74, 2m
3	41.4, CH_2_	1.55, m; 1.45, m	76.8, CH	3.42, dd (11.4, 4.5)	77.7, CH	3.40, dd (11.1, 5.0)
4	35.4, C		40.7, C		41.8, C	
5	142.8, C		143.3, C		145.6, C	
6	144.4, C	6.73, s (OH)	145.0, C		144.9, C	
7	181.6, C		182.1, C		183.3, C	
8	133.7, C		133.8, C		134.9, C	
9	74.1, C		73.6, C		76.1, C	
10	45.1, C		44.6, C		46.7, C	
11α	25.2, CH_2_	1.81, ddd (12.4, 3.2, 2.4)	25.5, CH_2_	1.74, m	65.8, CH	
11β		1.93, ddd (12.4, 12.4, 3.2)		2.03, ddd (13.6, 13.6, 3.0)		4.23, dd (12.1, 4.2)
12α	29.5, CH_2_	1.88, ddd (12.4, 12.4, 3.2)	29.1, CH_2_	1.92, ddd (13.6, 13.6, 3.0)	39.9, CH_2_	1.79, m
12β		1.58, m		1.59, m		1.65, ddd (12.1, 4.2, 1.9)
13	38.8, C		38.4, C		40.1, C	
14	147.9, CH	7.04, d (1.8)	147.9, CH	6.95, d (1.8)	147.0, CH	6.94, d (1.8)
15	145.4, CH	5.87, dd (17.5, 10.7)	145.4, CH	5.94, dd (17.5, 10.7)	146.1, CH	5.89, dd (17.5, 10.7)
16a	112.6, CH_2_	5.10, dd (17.5, 0.7)	111.3, CH_2_	5.13, dd (17.5, 0.9)	112.3, CH_2_	5.13, dd (17.5, 0.8)
16b		5.06, dd (10.7, 0.7)		5.07, dd (10.7, 0.9)		5.05, dd (10.7, 0.8)
17	23.3, CH_3_	1.13, s	22.3, CH_3_	1.15, s	24.0, CH_3_	1.18, s
18	30.1, CH_3_	1.41, s	24.9, CH_3_	1.55, s	25.9, CH_3_	1.54, s
19	26.9, CH_3_	1.29, s	18.1, CH_3_	1.25, s	19.0, CH_3_	1.21, s
20	29.4, CH_3_	1.21, s	28.7, CH_3_	1.22, s	29.2, CH_3_	1.24, s

^a^ Measured in CDCl_3_; ^b^ Measured in CD_3_OD. 500 MHz for ^1^H NMR; 125 MHz for ^13^C NMR.

Scopararane D (**2**) was obtained as colorless crystals. Its molecular formula, C_20_H_28_O_4_, was determined by HR-EI-MS (*m/z* 332.1983, [M]^+^, C_20_H_28_O_4_^+^; calcd 332.1982) indicating the presence of seven degrees of unsaturation. Comparison of the ^1^H and ^13^C NMR data with those of the scopararane C (**1**) revealed that the signals were similar, except one methylene signal at [δ_H_ 1.55 (m), 1.45 (m); δ_C_ 41.4] in **1** was absent, and one new hydroxyl-bearing methine was present in **2** at [δ_H_ 3.42 (dd, *J* = 11.4, 4.5 Hz); δ_C_ 76.8], which suggested that scopararane D (**2**) was the 3-hydroxyl derivative of scopararane C (**1**). The HMBC correlations observed between the proton at δ_H_ 3.42 (H-3) and the ^13^C signals at δ_C_ 40.7 (C-4), 28.8 (C-1), 24.9 (C-18), and 18.1 (C-19), further confirmed the assignment of new hydroxyl group to the 3-position. The entire sequence of H-atoms and the C-skeleton of **2** was established by ^1^H, ^1^H-COSY and HMBC. The relative configuration of **2** was identical to that in libertellenone A (**6**) and scopararane C (**1**) according to proton coupling constants and NOESY spectrum data. The proton coupling constants of H-11β (ddd, *J* = 13.6, 13.6, 3.0 Hz), H-1α (ddd, *J* = 13.0, 13.0, 4.1 Hz), and H-3α (dd, *J* = 11.4, 4.5 Hz), indicated the axial orientations of H-11β, H-1α, and H-3α. H-11β showed NOE correlations with H-17 and H-20, and H-20 showed NOE correlation with H-19, indicated the beta-axial positions of Me-17, Me-19, and Me-20. H-3α showed NOE correlations with H-1α and H-18, in agreement with the beta-equatorial position of 3-OH.

Scopararane E (**3**) was obtained as colorless crystals, which were analyzed for the molecular formula C_20_H_28_O_5_ (seven unsaturation equivalents) by HR-EI-MS (*m/z* 371.1833, [M + Na]^+^, C_20_H_28_O_5_Na^+^; calcd 371.1834). The ^1^H NMR spectrum of **3** was similar to that of scopararane D (**2**), except the methylene proton signal at [δ_H_ 2.03 (ddd, *J* = 13.6, 13.6, 3.0 Hz), 1.74 (m)] in **2** was replaced by the oxymethine proton resonance in **3** at [δ_H_ 4.23 (dd, *J* = 12.1, 4.2 Hz)]. Comparison of the ^13^C NMR data with those of **2** showed analogy of the chemical shifts except for C-11 and C-12. The above evidence suggested that scopararane E (**3**) was the 11-hydroxyl derivative of scopararane D (**2**). The HMBC correlations observed between the proton at δ_H_ 4.23 (H-11) and the ^13^C signals at δ_C_ 39.9 (C-12), and 46.7 (C-10), further confirmed the assignment of new hydroxyl group to the 11-position. Analyses of ^1^H, ^1^H COSY and HMBC spectral data allowed complete assignment of protons and carbons in **3** (Table 1). The relative configuration of **3** was identical to that in libertellenone A (**6**), scopararane C (**1**), and scopararane D (**2**) according to proton coupling constants and NOESY spectrum data.

The absolute stereostructures of compounds **1**–**3** were determined on the basis of their CD spectra. The absolute configuration of libertellenone A had been established by the modified Mosher method and its CD spectra data had also been reported in the literature [14]. Our results showed that the CD spectra of compounds **1**–**3** were virtually identical to that of libertellenone A with all demonstrating a clear negative Cotton effect between 350 and 353 nm, which was derived from the π-π* transition of the cross-conjugated system. Compounds **1**–**3** also have the same positive Cotton effect as libertellenone A at 308–312 nm. Based on the above evidence, compounds **1**–**3** and **6** belong to the same stereochemical series. Thus, the absolute stereochemistry of **1** was established as 9*S*, 10*S*, and 13*R*, that of **2** as 3*S*, 9*S*, 10*S*, and 13*R*, and that of **3** as 3*S*, 9*S*, 10*S*, 11*R*, and 13*R*. 

Scopararane F (**4**) was obtained as colorless crystals, having a molecular formula of C_20_H_30_O_5_ (six unsaturation equivalents), determined by HR-EI-MS (*m/z* 373.1984, [M + Na]^+^, C_20_H_30_O_5_Na^+^; calcd 373.1991). The ^1^H, ^13^C, DEPT135 and HSQC NMR spectra of **4** showed 20 carbon signals and 26 protons for three singlet methyls, seven methylenes (one sp^2^, an oxygenated sp^3^, and five sp^3^), three methines (one sp^2^, and two oxygenated sp^3^), and seven quaternary carbons (two sp^2^, two oxygenated sp^3^, and three sp^3^). Comparison of the ^1^H and ^13^C NMR data of **4** with those of scopararane B (**7**) [9,12] revealed similar signals, except one hydroxyl-bearing double doublet methine signal at [δ_H_ 4.42 (dd, *J* = 6.3, 4.8 Hz); δ_C_ 70.0], and the α,β-unsaturated ketone C-7 (δ_C_ 194.6) in **7** were absent, and one new methylene at [δ_H_ 2.19 (ddd, *J* = 13.6, 5.8, 2.9 Hz), 1.92 (m); δ_C_ 21.7], and a hydroxyl-bearing singlet methine signal at [δ_H_ 4.48 (s); δ_C_ 74.6] were present in **4**. The new methylene protons and singlet methine signal were then attributed to H_2_-11 and H-7 respectively. HMBC cross-peaks of H-7 with C-6, C-8, and C-9, and H-11 with C-8, C-9, C-12, and C-13, further confirmed the 7-position of the methine and 11-position of the methylene respectively. Analyses of ^1^H, ^1^H COSY and HMBC spectral data allowed complete assignment of protons and carbons in **4** (Table 2). The relative configuration of **4** was determined by analysis of the ^1^H–^1^H *J*-value and NOESY data (Figure 3). The small coupling constants of H-11α [δ_H_ 2.19 (dd, *J* = 5.8, 2.9 Hz)] with H-12, indicated the axial location of H-11β (δ_H_ 1.92). H-11β showed NOE correlations with H-17 and H-20b [δ_H_ 3.40 (d, *J* = 9.2 Hz)], and H-20a [δ_H_ 4.13 (d, *J* = 9.2 Hz)] showed NOE correlation with H-19, indicating the beta-axial orientation of Me-17, Me-19, and H-20. H-14 showed NOE correlations with H-7 and H-17, in agreement with alpha-axial positions of 7-OH and 14-OH. The relative configurations of C-7 and C-14 were identical to that in sphaeropsidin E [16]. Compound **4** has the same negative optical rotation as compounds **1**–**3** and **6**, and also has the same relative configuration as compounds **1**–**3** and **6**. On the base of biogenetical reasons, the absolute configuration of compound **4** can be assumed to be similar to those of compounds **1**–**3** and **6**. Accordingly, the absolute stereochemistry of **4** was established as 6*R*, 7*R*, 8*S*, 10*R*, 13*R*, and 14*S*.

**Table 2:** ^1^H and^ 13^C NMR data of compounds **4**–**5**.

Position	4 ^b^		5 ^a^	
	δ_C__, _mult.	δ_H_ ( *J *in Hz)	δ_C__, _mult.	δ_H_ ( *J *in Hz)
1	24.8, CH_2_	1.55, 2m	29.1, CH_2_	1.63, 2m
2	18.9, CH_2_	1.61, 2m	17.9, CH_2_	1.67, 2m
3	38.7, CH_2_	1.64, m; 1.11, m	36.3, CH_2_	1.71, m; 1.53, m
4	37.4, C		36.7, C	
5	80.0, C		82.6, C	
6	107.2, C		210.9, C	
7	74.6, CH	4.48, s	74.6, CH	5.23, s
8	132.6, C		131.8, C	
9	141.7, C		139.8, C	
10	52.1, C		50.7, C	
11α	21.7, CH_2_	2.19, ddd (13.6, 5.8, 2.9)	21.2, CH_2_	2.15, m
11β		1.92, m		1.90, m
12α	28.0, CH_2_	1.80, m	30.2, CH_2_	1.83, m
12β		1.41, m		1.49, m
13	39.6, C		39.7, C	
14	70.6, CH	4.01, s	72.4, CH	4.13, s
15	146.2, CH	6.07, dd (17.5, 11.1)	142.0, CH	6.03, dd (17.3, 1.4)
16a	112.1, CH_2_	5.07, dd (17.5, 1.5)	114.8, CH_2_	5.23, dd (17.3, 1.4)
16b		5.05, dd (11.1, 1.5)		5.21, dd (11.4, 1.4)
**Position**	**4 ^b^**		**5 ^a^**	
	**δ_C__, _mult.**	**δ_H_ (*J *in Hz)**		**δ_C__, _mult.**
17	21.3, CH_3_	0.93, s	22.8, CH_3_	1.08, s
18	24.3, CH_3_	1.46, s	23.8, CH_3_	1.46, s
19	28.9, CH_3_	1.23, s	27.8, CH_3_	1.07, s
20	72.4, CH_2_	4.13, d (9.2)	23.5, CH_3_	1.07, s
		3.40, d (9.2)		

^a^ Measured in CDCl_3_; ^b^ Measured in CD_3_OD. 500 MHz for ^1^H NMR; 125 MHz for ^13^C NMR.

Scopararane G (**5**) was obtained as a colorless oil. The HR-EI-MS (*m/z *334.2138, [M]^+^, C_20_H_30_O_4_^+^; calcd 334.2144) indicated a molecular formula of C_20_H_30_O_4_, requiring six unsaturations. The ^1^H and ^13^C NMR spectra of **5** were similar to those of scopararane F (**4**), except for the missing signal of the oxygenated methylene at [δ_H_ 4.13 (d, *J* = 9.2 Hz), 3.40 (d, *J* = 9.2 Hz); δ_C_ 72.4] in **4**, the presence of a singlet methyl at [δ_H_ 1.07 (s); δ_C_ 23.5] in **5**, and the replacement from the oxygenated quaternary C-6 at (δ_C_ 107.2) in **4** to the carbonyl resonance at (δ_C_ 210.9) in **5**. Based on these spectral data, scopararane G (**5**) was assigned as the ketone derivative of scopararane F (**4**). HMBC cross-peaks of H-20 with C-1, C-5, and C-9, and H-7 with C-6, C-8, and C-9, further confirmed the 20-position of the methyl and 6-position of the carbonyl respectively. The entire sequence of H-atoms and the C-skeleton of **5** was established by ^1^H, ^1^H COSY and HMBC (Table 2). The relative configurations of rings A and C were identical to those in **4,** and the relative configurations of C-7 and C-14 were also identical to those in sphaeropsidin E [16] according to NOE results. The absolute stereochemistry of **5** was assigned by comparison its CD spectra with those of sphaeropsidin E [16]. Compound **5** showed similar Cotton effect to sphaeropsidin E at 277 nm, 250 nm, and 227 nm. Due to the presence of carbonyl in B ring of **5**, an additional negative Cotton effect was also observed at 342 nm. Based on the evidence above, in addition to biogenetical reasons, the absolute stereochemistry of **5** was established as 6*R*, 8*S*, 10*R*, 13*R*, and 14*S*.

### 2.2. Cytotoxic Activities

Compounds **1**–**11** were evaluated for their *in vitro* cytotoxic activities against three human cell lines, including SF-268 (human glioma cell line), MCF-7 (human breast adenocarcinoma cell line) and NCI-H460 (human non-small cell lung cancer cell line) by the MTT method. The results of the cytotoxicity tests suggested that all the analogues demonstrated quite different cytotoxic activities (Table 3). Diaporthein B (**9**) exhibited strong cytotoxicities against SF-268, MCF-7 and NCI-H460 cell growth with IC_50_ values ranging from 4.4–9.9 μM. Libertellenone A (**6**) displayed varying cytotoxic activities against the three cell lines with IC_50_ values in the range of 12.0–40.2 μM. Scopararanes C (**1**) and D (**2**) showed moderate cytotoxic activities against the tumor cell line MCF-7 with IC_50_ values of 35.9 μM and 25.6 μM, respectively. The other compounds displayed no appreciable activity, except that compounds **3**, **5**, and **7** showed weak cytotoxic activities against the MCF-7 cell line with IC_50_ values of 74.1 μM, 85.5 μM, and 60.1 μM, respectively.

**Table 3:** Cytotoxic activities of compounds **1**–**11**.

Compounds	IC_50_ (μM)		
	SF-268	MCF-7	NCI-H460
**1**	>100	35.9	>100
**2**	43.5	25.6	46.1
**3**	>100	74.1	>100
**4**	>100	>100	>100
**5**	>100	85.5	>100
**6**	20.5	12.0	40.2
**7**	80.1	60.1	>100
**8**	>100	>100	>100
**9**	9.2	4.4	9.9
**10**	>100	>100	>100
**11**	>100	>100	>100
**Cisplatin**	4.0	9.2	1.5

Data for compounds **6** to **11** were taken from [12].

By comparing the structures of diaporthein B (**9**) with diaporthein A (**8**), scopararane B (**7**), and libertellenone A (**6**), we found that compound **9** displayed strong analogies with compounds **8** and **7**, differing only in the presence of a ketone moiety at C-7 replacing a hydroxy group, and a ∆^8,^^ 14^ olefinic bond instead of a ∆^8,^^ 9^ olefinic bond, respectively; compound **9** exhibited high structural similarity with compound **6** and the main structural difference between them is at position C-20. It also should be noted that the only structural differences between libertellenone A (**6**) and scopararanes C–E (**1**–**3**) are the number and positions of hydroxyl groups. All the above suggested that the carbonyl at position C-7, ∆^8,14^ olefinic bond, whether the C-20 methyl forms a furan ring with C-6, C-5, and C-10, and the number and positions of hydroxyl groups, might be the important structural factors leading to the distinction of their cytotoxicities. Diaporthein B and the 11-dehydroxy derivative of diaporthein B (scopararane A) were reported to show significant cytotoxic activities against human cancer cell lines (KB, MCF-7, NCI-H187) and nonmalignant Vero cells (African green monkey kidney fibroblasts) [10,17], which further demonstrates that the structural features described above are important determinants of cytotoxic activity. The target of the cytotoxic effect of these compounds is still unknown.

## 3. Experimental Section

### 3.1. General Experimental Procedures

^1^H, ^13^C NMR and DEPT spectra and 2D NMR were recorded on a Bruker Avance-500 spectrometer with TMS as internal standard; at 500/125 MHz, respectively; δ in ppm, *J* in Hz. HR-EI-MS were measured on a Thermo MAT95XP High Resolution mass spectrometer and EIMS on a Thermo DSQ EI-mass spectrometer; in *m/z*. Optical rotations were measured in methanol using a Perkin-Elmer 341 polarimeter from Perkin Elmer. CD measurements were carried out under N_2_ gas on a Jasco 820 spectropolarimeter. IR spectra were recorded on a Bruker EQUINOX 55 spectrophotometer. UV spectra were measured on a Biochrom Ultrospec 6300 pro UV-visible spectrophotometer; λ_max_ (log ε) in nm. Column chromatography (CC) was performed with silica gel (SiO_2_; 200–300 mesh; Qingdao Haiyang Chemical Group Co., Qingdao, China), Chromatorex ODS (40–75 μm; Fuji Silysia), and Sephadex LH-20 (Amersham Biosciences). TLC was conducted with precoated glass plates GF-254 (Qingdao Haiyang Chemical Group Co., Qingdao, China). Melting points were measured with a Netzsch DSC 204 apparatus. Solvents were of the industrial purity and distilled prior to use.

### 3.2. Fungal Material and Identification

The strain FS26 was isolated from a marine sediment sample, which was collected at the depth of 139 m in the South China Sea (18°42.878′N，111°2.797′E), in August 2008. The strain was identified by sequence analysis of the rDNA ITS (internal transcribed spacer) region. The sequence of the ITS region of the marine fungus *E. scoparia* FS26 has been submitted to GenBank (Accession No. HM989831). By using BLAST (nucleotide sequence comparison program) to search the GenBank database, *E. scoparia* FS26 has 100% similarity to *E. scoparia* BAFC 3321 (Accession No. EU702431.1). The strain is preserved at the Guangdong Provincial Key Laboratory of Microbiol Culture Collection and Application, Guangdong Institute of Microbiology [11,12].

### 3.3. Fermentation, Extraction and Isolation

*E. scoparia* FS26 was cultured in potato dextrose broth (potato 20%, glucose 2%, K_2_HPO_4_ 0.3%, MgSO_4_·7H_2_O 0.15%, vitamin B_1_ 10 mg/L), which was prepared in 50% (v/v) sea water instead of distilled water. The fungus *E. scoparia* FS26 was maintained on 50% (v/v) sea water potato dextrose agar medium at 28 °C for 5 days, and then three pieces (0.5 × 0.5 cm^2^) of mycelial agar plugs were inoculated into 20 × 500 mL Erlenmeyer flasks, each containing 250 mL 50% (v/v) sea water potato dextrose broth. After 6 days of incubation at 28 °C on a rotary shaker at 130 r/m, 25 mL seed cultures was aseptically transferred into a total of 200 flasks (1000 mL) containing 500 mL of 50% (v/v) sea water potato dextrose broth. The liquid cultivation that followed was kept for 7 days at 28 °C and 130 r/m on a rotary shaker.

The culture (100 L) was centrifuged to give the broth and mycelia. The broth was exhaustively extracted with EtOAc four times, then the EtOAc layers were combined and evaporated under reduced pressure at a temperature not exceeding 40 °C to yield a dark brown gum (24.8 g). Fungal mycelia were thoroughly extracted with EtOAc four times and then the extracts were combined and concentrated under reduced pressure to give a mycelial crude extract (132.4 g). Both crude EtOAc extracts were subjected to silica gel column chromatography (200–300 mesh) with a gradient system of increasing polarity (petroleum ether/AcOEt/MeOH), to afford 20 fractions (Fr.A–Fr.V) for the liquid culture and 20 fractions for the mycelia (Fr.1–Fr.20), respectively. 

The Fr.F eluting with 15:1 petroleum ether-EtOAc was subjected to CC on Sephadex LH-20 (CHCl_3_/MeOH, 1:1), to yield compound **1** (114.2 mg). The Fr.M eluting with 3:1 petroleum ether-EtOAc was subjected to CC on Sephadex LH-20 (CHCl_3_/MeOH, 1:1), to give 2 sub-fractions Fr.M-1 and Fr.M-2. Fr.M-2 was first purified by CC over reversed-phase silica gel C_18_ (MeOH/H_2_O, 60:40), then followed by CC (SiO_2_; petroleum ether/AcOEt, 5:1) to get compound **5** (57.1 mg). The Fr.N eluting with 7:3 petroleum ether-EtOAc was subjected to CC on Sephadex LH-20 (CHCl_3_/MeOH, 1:1), and then followed by CC over reversed-phase silica gel C_18_ (MeOH/H_2_O, 50:50 to 70:30) to yield compounds **2** (36.6 mg). The Fr.P eluting with 1:1 petroleum ether-EtOAc was subjected to CC on Sephadex LH-20 (CHCl_3_/MeOH, 1:1), and followed by CC over reversed-phase silica gel C_18_ (MeOH/H_2_O, 40:60 to 60:40) to yield 3 sub-fractions Fr.P-1 to Fr.P-3. Fr.P-2 was further purified by CC (SiO_2_; petroleum ether/AcOEt, 2:1) to get compound **3** (8.0 mg). The Fr.14 eluting with 2:1 petroleum ether-EtOAc was subjected to CC on Sephadex LH-20 (CHCl_3_/MeOH, 1:1), followed by CC over reversed-phase silica gel C_18_ (MeOH/H_2_O, 70:30) and CC (SiO_2_; petroleum ether/AcOEt, 2:1) to get compound **4** (11.1 mg).

### 3.4. Characterization Data

Scopararane C (**1**): colorless crystals; mp 170.4 °C; [*α*]^25^_D_ −160.40 (*c* 1.53, CHCl_3_); UV (MeOH) λ_max_ (log ε) 219 (3.75), 263 (3.68), 325 (3.92) nm; CD (CH_3_CN) (∆ε) 352 (−32.4), 308 (+7.3); IR (KBr) *v*_max_ 3528, 3412, 3011, 2954, 2924, 2866, 1652, 1622, 1590, 1462, 1394, 1369, 1287, 1211, 1122, 1085, 1046, 997, 842, 806, 780, 679, 545 cm^−1^; ^1^H and ^13^C NMR, see Table 1; HR-EI-MS *m/z* 316.2032 [M]^+^(calcd for C_20_H_28_O_3_^+^, 316.2033).

Scopararane D (**2**): colorless crystals; mp 197.4 °C; [*α*]^25^_D_ −200.36 (*c* 0.84, CHCl_3_); UV (MeOH) λ_max_ (log ε) 218 (3.94), 260 (3.81), 325 (4.02) nm; CD (CH_3_CN) (∆ε) 351 (−37.7), 310 (+9.1); IR (KBr) *v*_max_ 3522, 3410, 3089, 2968, 2877, 1650, 1620, 1591, 1465, 1392, 1369, 1289, 1231, 1206, 1142, 1085, 1044, 1012, 957, 919, 878, 806, 780, 679, 548 cm^−1^; ^1^H and ^13^C NMR, see Table 1; HR-EI-MS *m/z* 332.1983 [M]^+^ (calcd for C_20_H_28_O_4_^+^, 332.1982).

Scopararane E (**3**): colorless crystals; colorless crystals; mp 100.0 °C; [*α*]^25^_D_ −92.30 (*c* 0.77, MeOH); UV (MeOH) λ_max_ (log ε) 218 (4.02), 259 (3.90), 324 (4.12) nm; CD (CH_3_CN) (∆ε) 350 (−36.4), 312 (+15.1); IR (KBr) *v*_max_ 3417, 3087, 2970, 2941, 2874, 1653, 1622, 1592, 1464, 1396, 1374, 1333, 1289, 1230, 1153, 1064, 1041, 1017, 1002, 968, 916, 877, 835, 778, 731, 678, 545 cm^−1^; ^1^H and ^13^C NMR, see Table 1; HR-EI-MS *m/z* 371.1833 [M *+ *Na]^+^ (calcd for C_20_H_28_O_5_ Na^+^, 371.1834).

Scopararane F (**4**): colorless crystals; mp 170.0 °C; [*α*]^25^_D_ −94.70 (*c* 0.66, MeOH); UV (MeOH) λ_max_ (log ε) 207 (3.41) nm; CD (CH_3_CN) (∆ε) 207 (−103.4); IR (KBr) *v*_max_ 3382, 3086, 2932, 1641, 1460, 1393, 1302, 1247, 1212, 1180, 1125, 1001, 949, 914, 877, 845, 804, 709 cm^−1^; ^1^H and ^13^C NMR, see Table 2; HR-EI-MS *m/z* 373.1984 [M *+ *Na]^+^ (calcd for C_20_H_30_O_5_Na^+^, 373.1991).

Scopararane G (**5**): a colorless oil; [*α*]^25^_D_ +1.43 (*c* 0.42, MeOH); UV (MeOH) λ_max_ (log ε) 206 (3.67), 232 (3.45), 278 (3.21) nm; CD (CH_3_CN) (∆ε) 342 (−0.8), 277 (+0.4), 250 (−1.1), 227 (+1.8); IR (KBr) *v*_max_ 3436, 2960, 2928, 2874, 1725, 1461, 1379, 1286, 1125, 1074, 1043, 844, 805, 745 cm^−1^; ^1^H and ^13^C NMR, see Table 2; HR-EI-MS *m/z* 334.2138 [M]^+^ (calcd for C_20_H_30_O_4_^+^, 334.2144).

Libertellenone A (**6**): colorless crystals; CD (CH_3_CN) (∆ε) 353 (−46.3), 311 (+13.8).

### 3.5. Cytotoxicity Assay

Cytotoxicities of compounds (**1**–**5**) were assayed against three tumor cell lines MCF-7, NCI-H460 and SF-268. Assays were performed by the MTT method [18].

## 4. Conclusion

Investigation of biologically active secondary metabolites from the marine-derived fungus *Eutypella scoparia* in the South China Sea has resulted in the isolation of five novel oxygenated pimarane diterpenes, scopararanes C–G (**1**–**5**). Cytotoxic tests for the five novel compounds (**1**–**5**) and their related six analogues [12] displayed quite different cytotoxic activities. Study of the structure-activity relationship indicated that the carbonyl at position C-7, ∆^8,^^ 14^ olefinic bond, whether the C-20 methyl forms a furan ring with C-6, C-5, and C-10, and the number and positions of hydroxyl groups might be the important structural factors leading to the distinction of their cytotoxicities.

## Supplementary Files

Supplementary File 1: PDF-Document (PDF, 4722 KB)
