# Supplementary material for: Scopararanes C–G: New Oxygenated Pimarane Diterpenes from the Marine Sediment-Derived Fungus Eutypella scoparia FS26
Source: Mar Drugs. 2012 Feb 27;10(3):539–50. doi: 10.3390/md10030539 (PMC3347013; doi:10.3390/md10030539)
Supplement: Supplementary File 1: — PDF-Document (PDF, 4722 KB) [file marinedrugs-10-00539-s001.pdf]

Supplementary Material

Manuscript ID: marinedrugs-13384

Type of manuscript: Article

Title: Scopararanes C–G：New Oxygenated Pimarane Diterpenes from themarine sediment-derived fungus *Eutypella scoparia* FS26

Authors: Li Sun, Dongli Li, Meihua Tao, Yuchan Chen, Feijun Dan, Weimin Zhang

1H-NMR of scopararane C

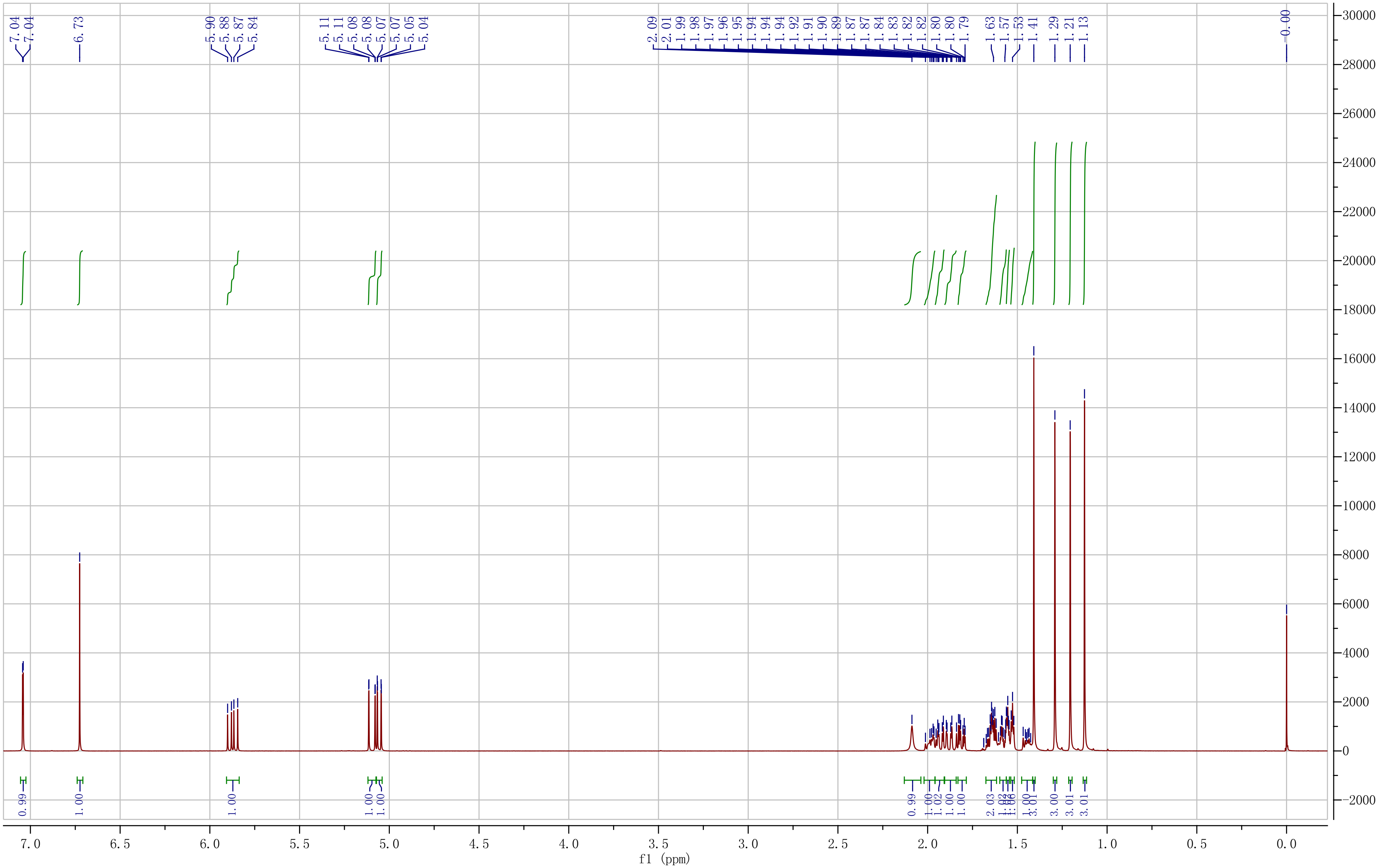

13C-NMR of scopararane C

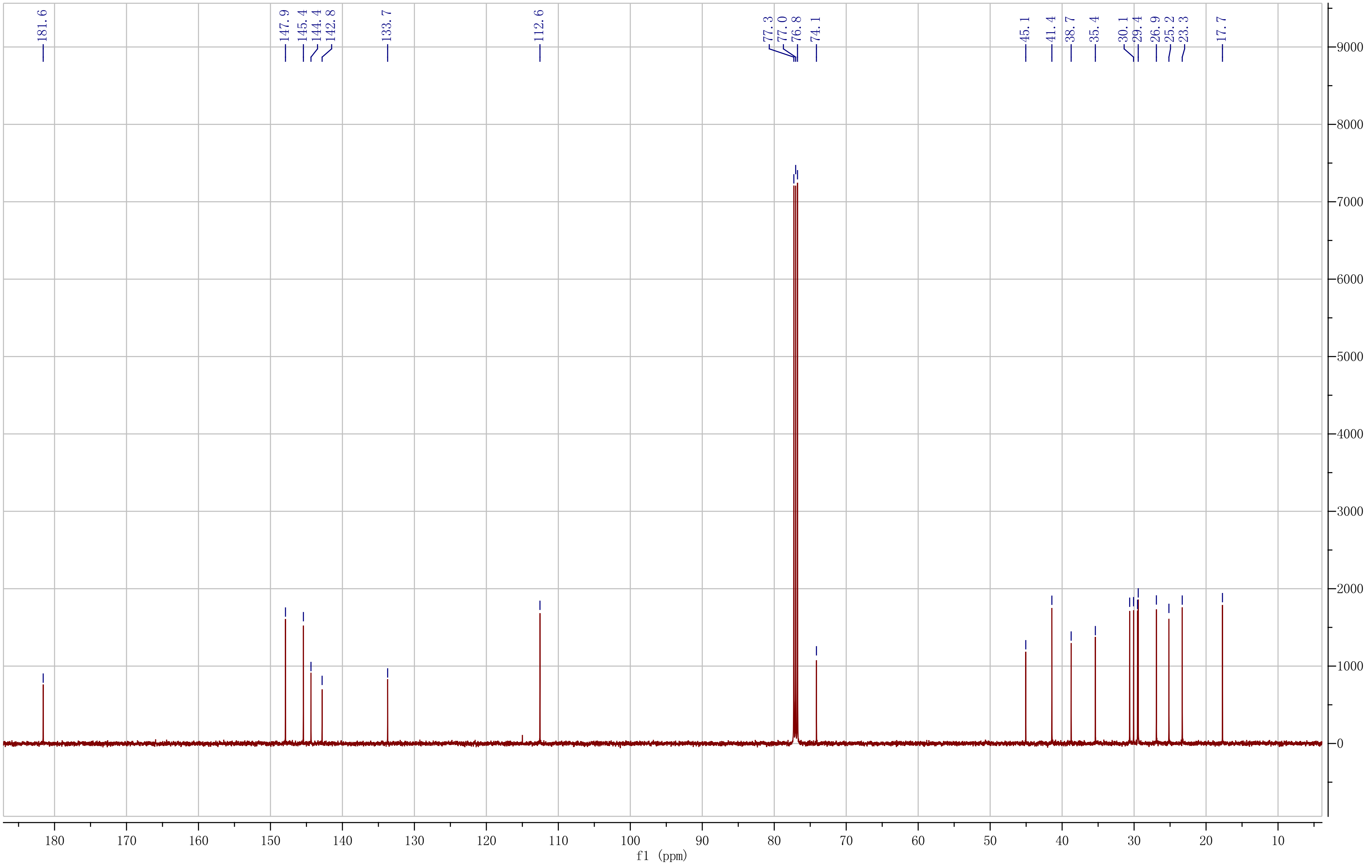

DEPT 135 of scopararane C

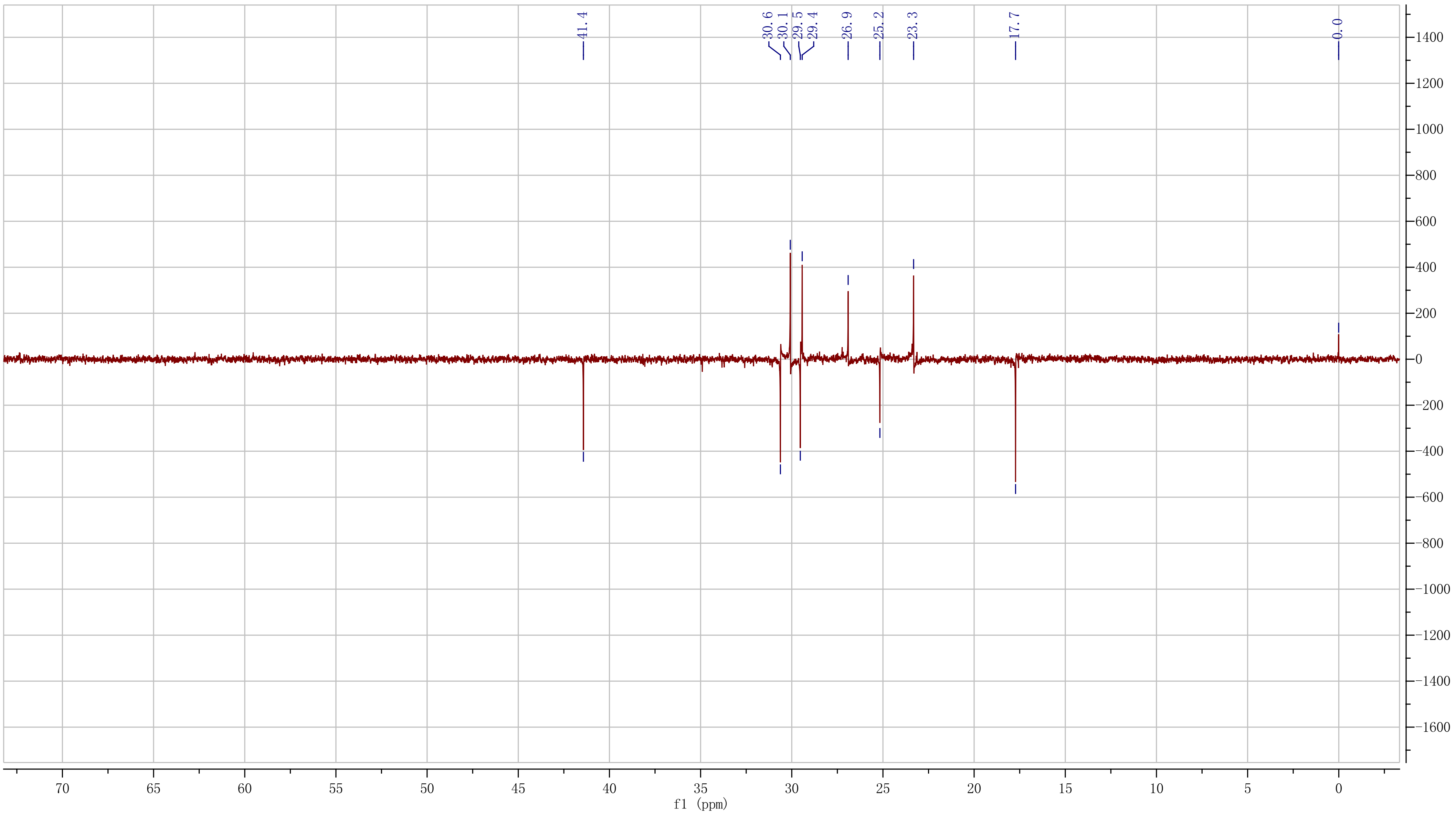

1H-1H COSY of scopararane C

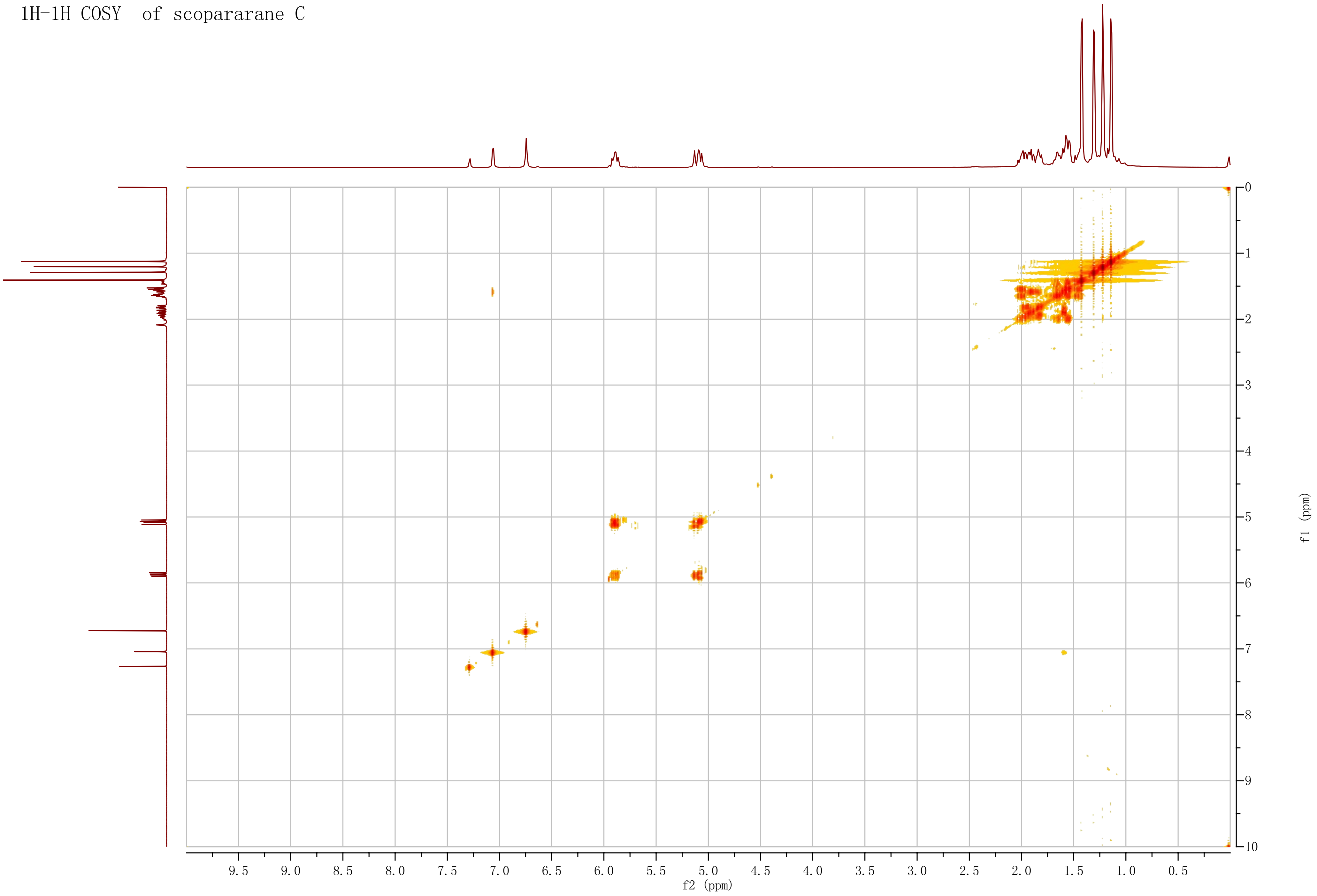

HSQC of scopararane C

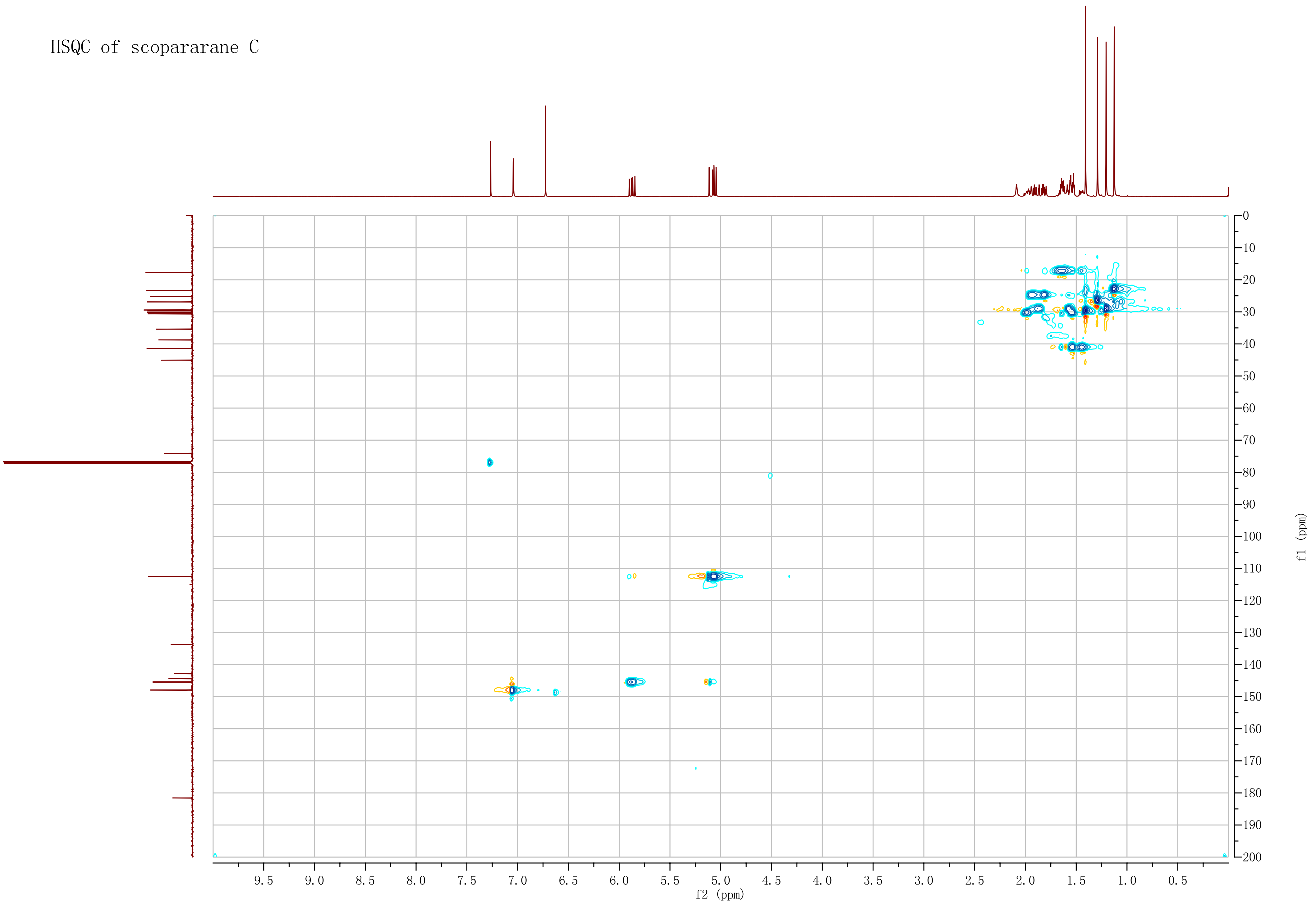

HMBC of scopararane C

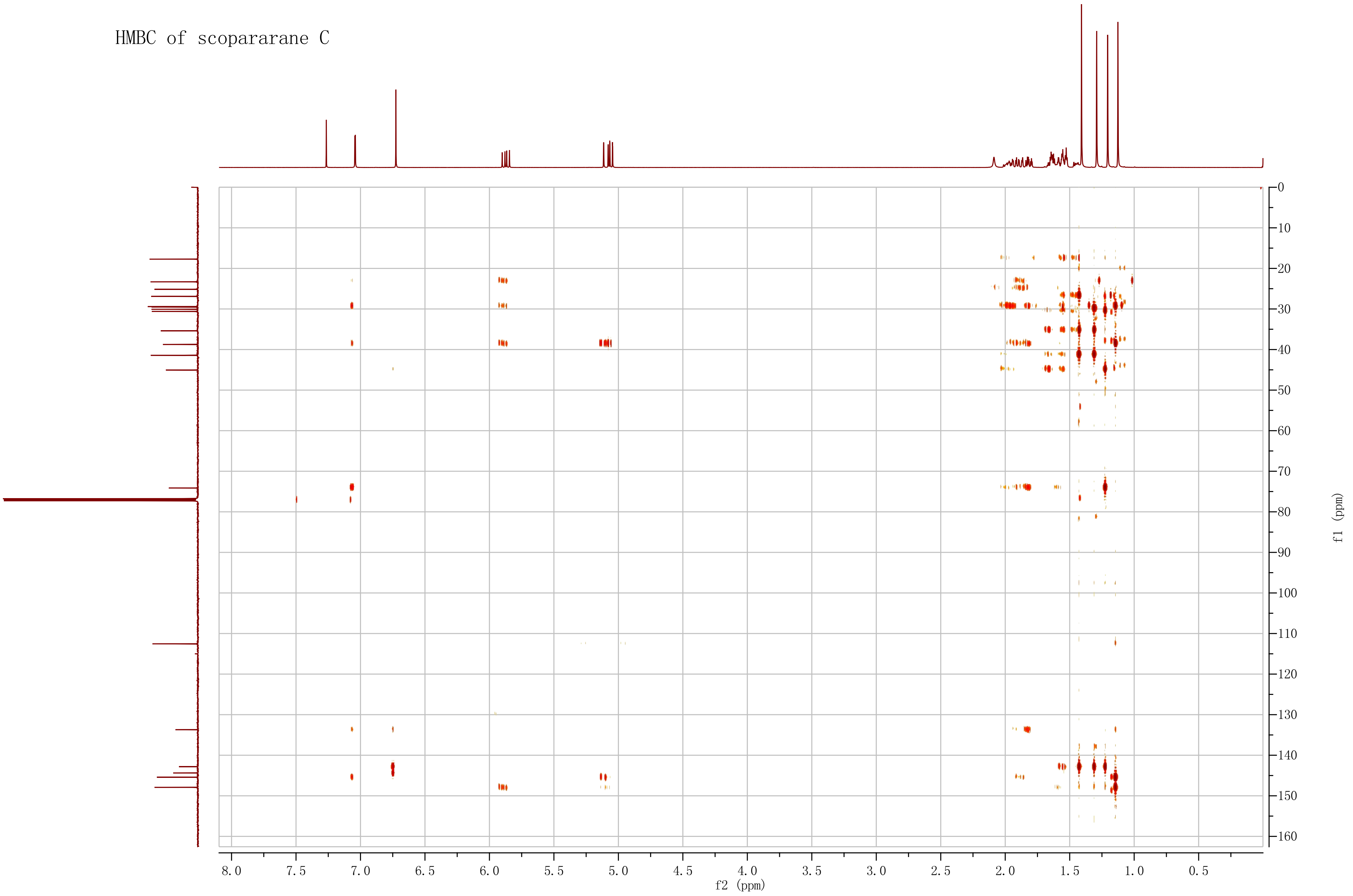

1H-NMR of scopararane D

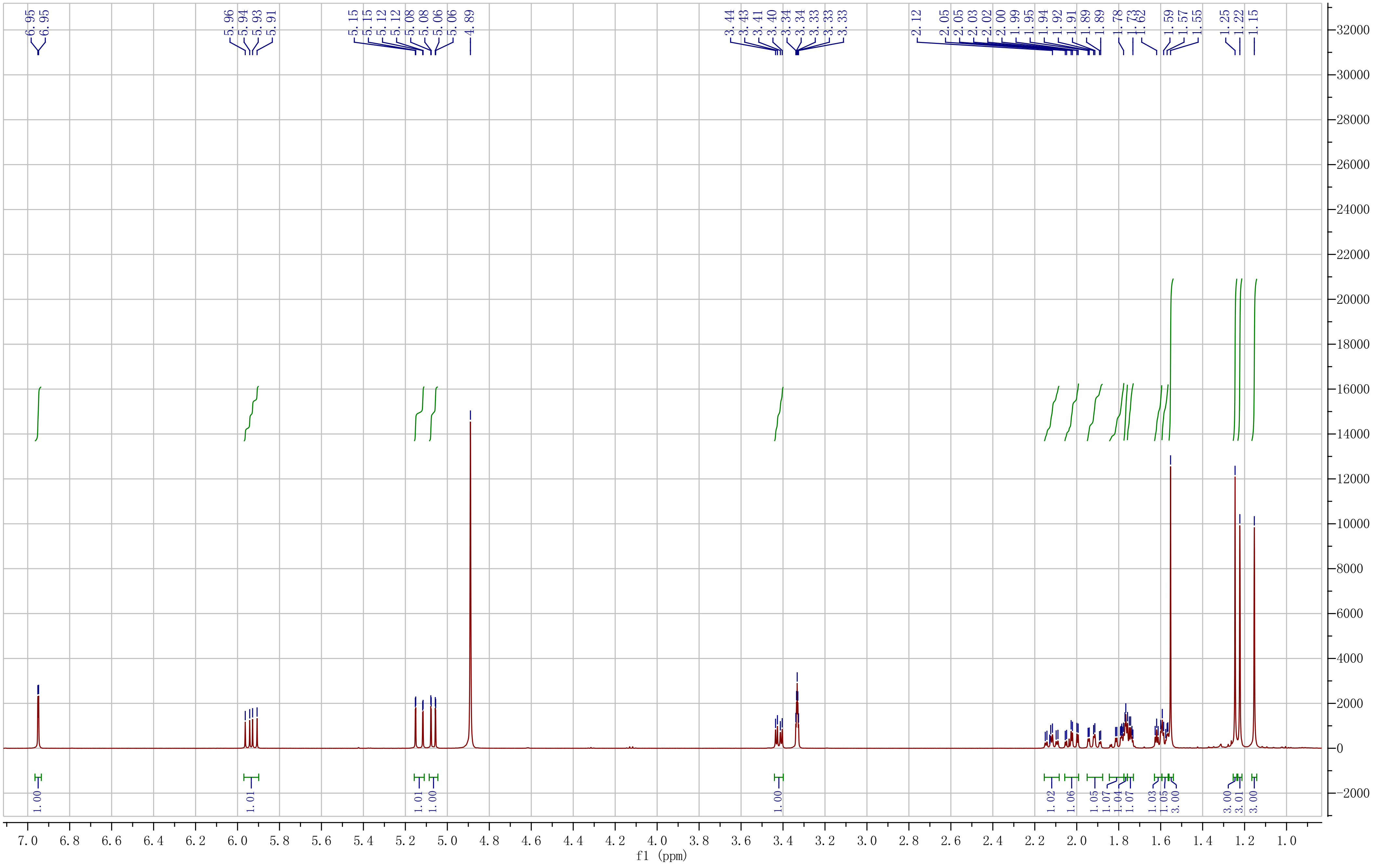

13C-NMR of scopararane D

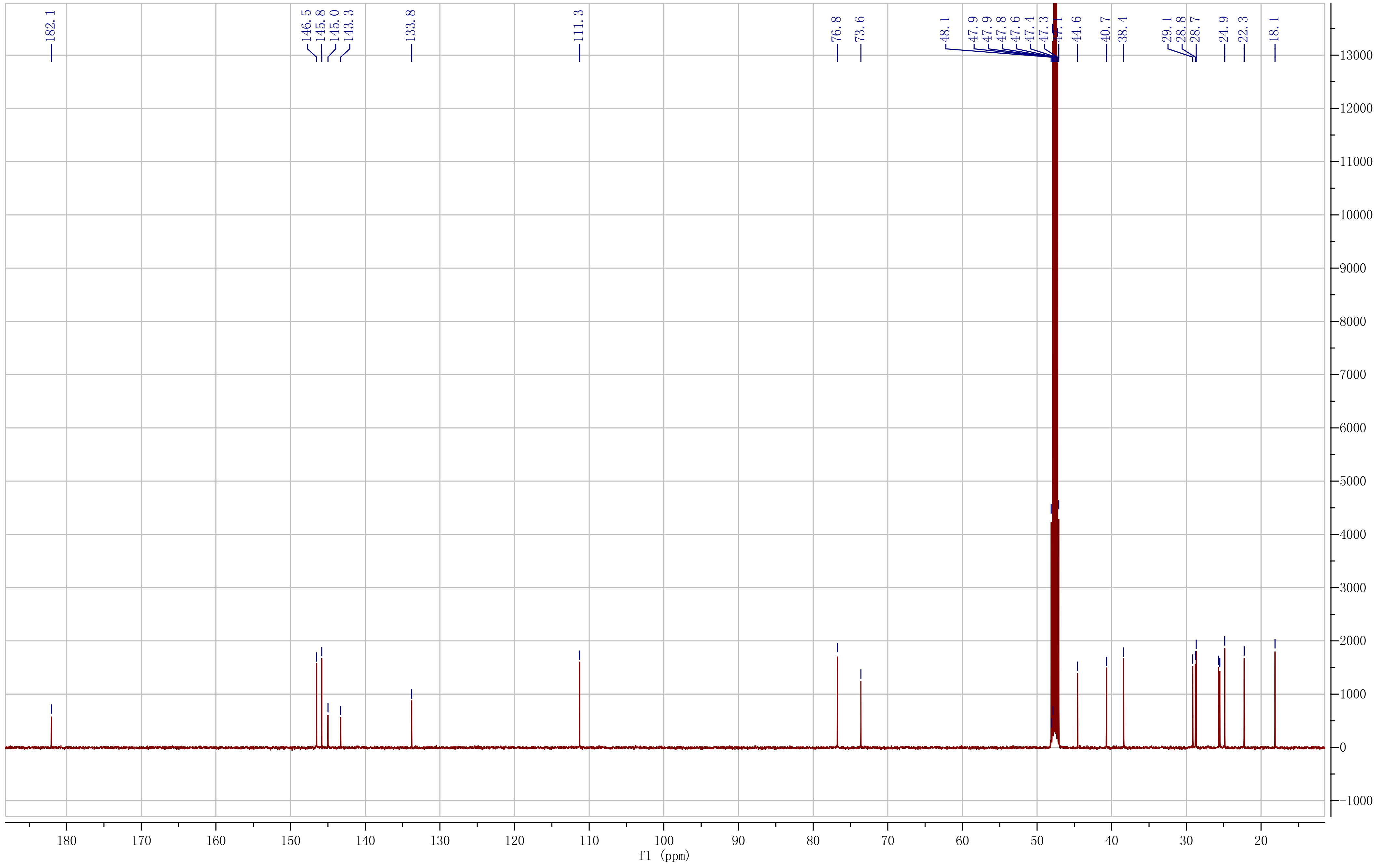

DEPT 135 of scopararane D

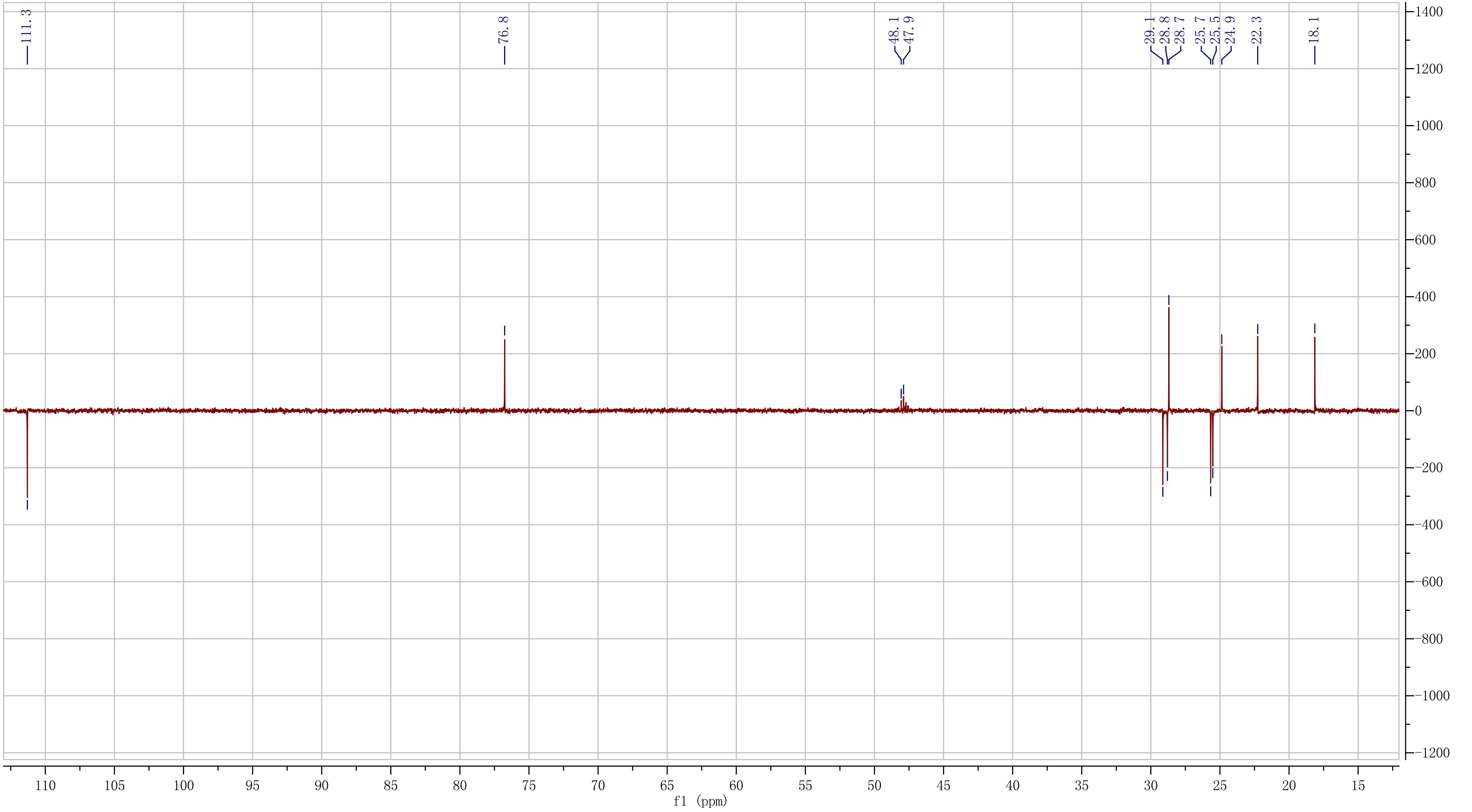

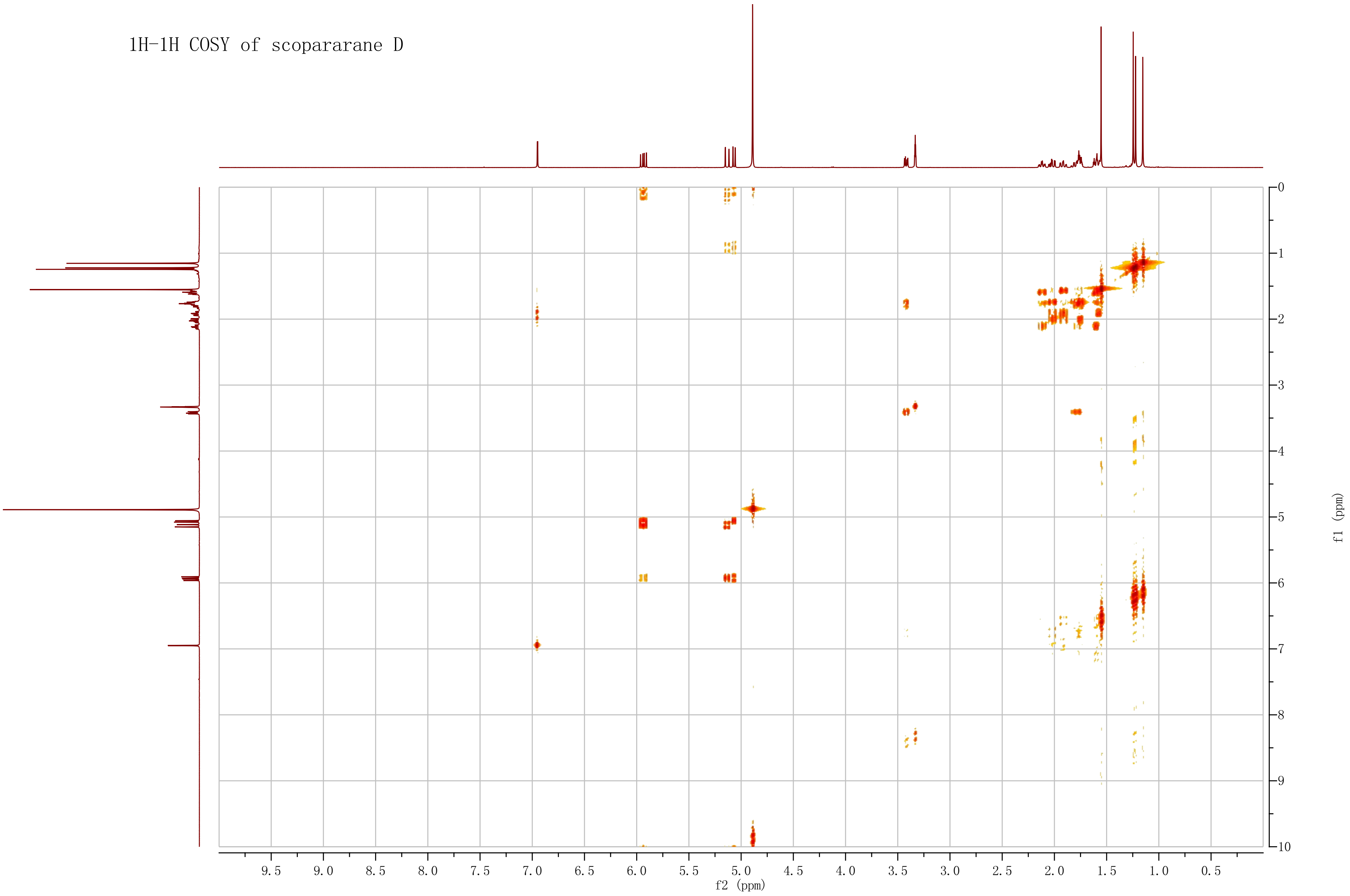
1H-1H COSY of scopararane D
f2 (ppm)
f1 (ppm)
9.5
9.0
8.5
8.0
7.5
7.0
6.5
6.0
5.5
5.0
4.5
4.0
3.5
3.0
2.5
2.0
1.5
1.0
0.5
0
1
2
3
4
5
6
7
8
9
10

HSQC of scopararane D

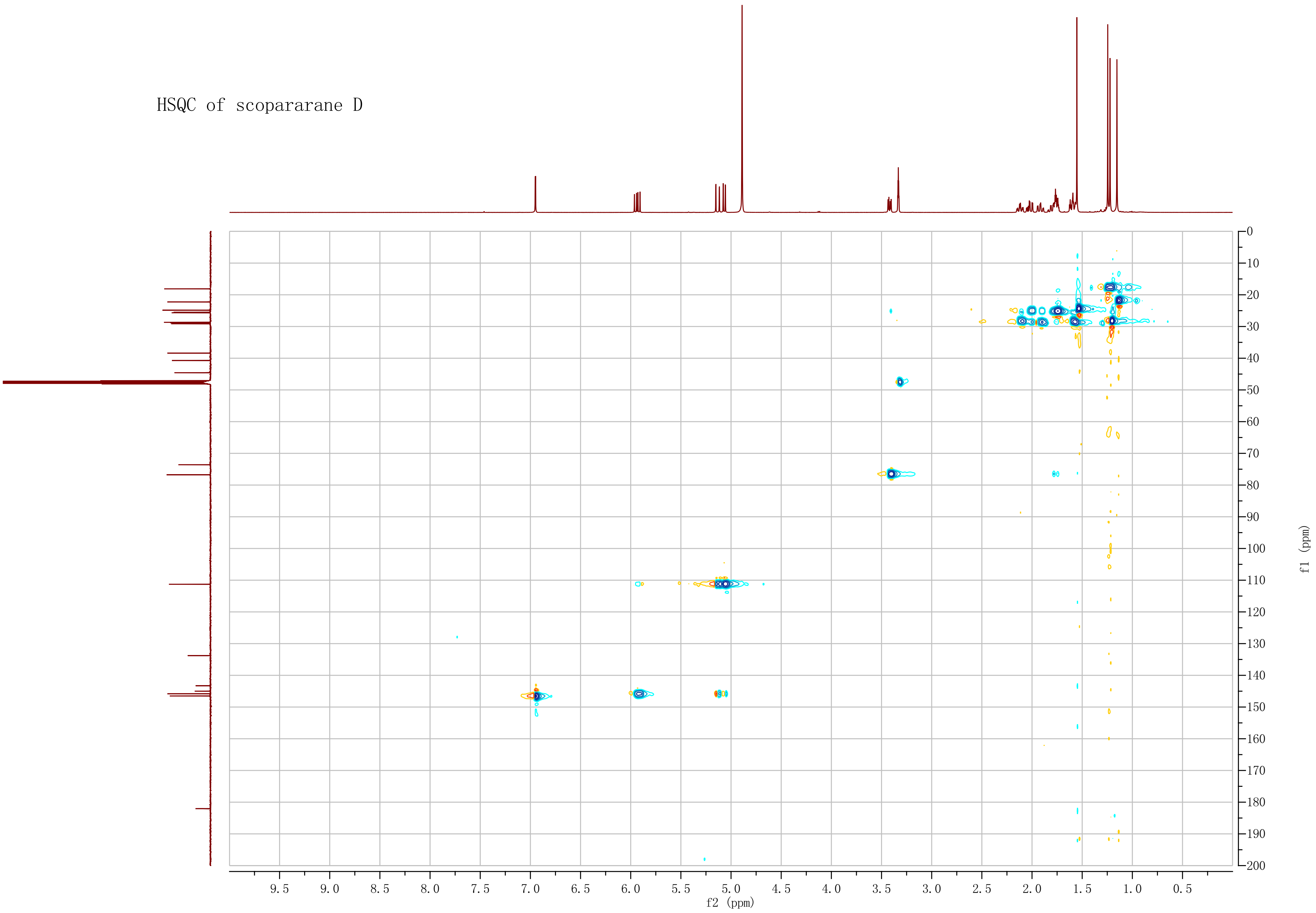

HMBC of scopararane D

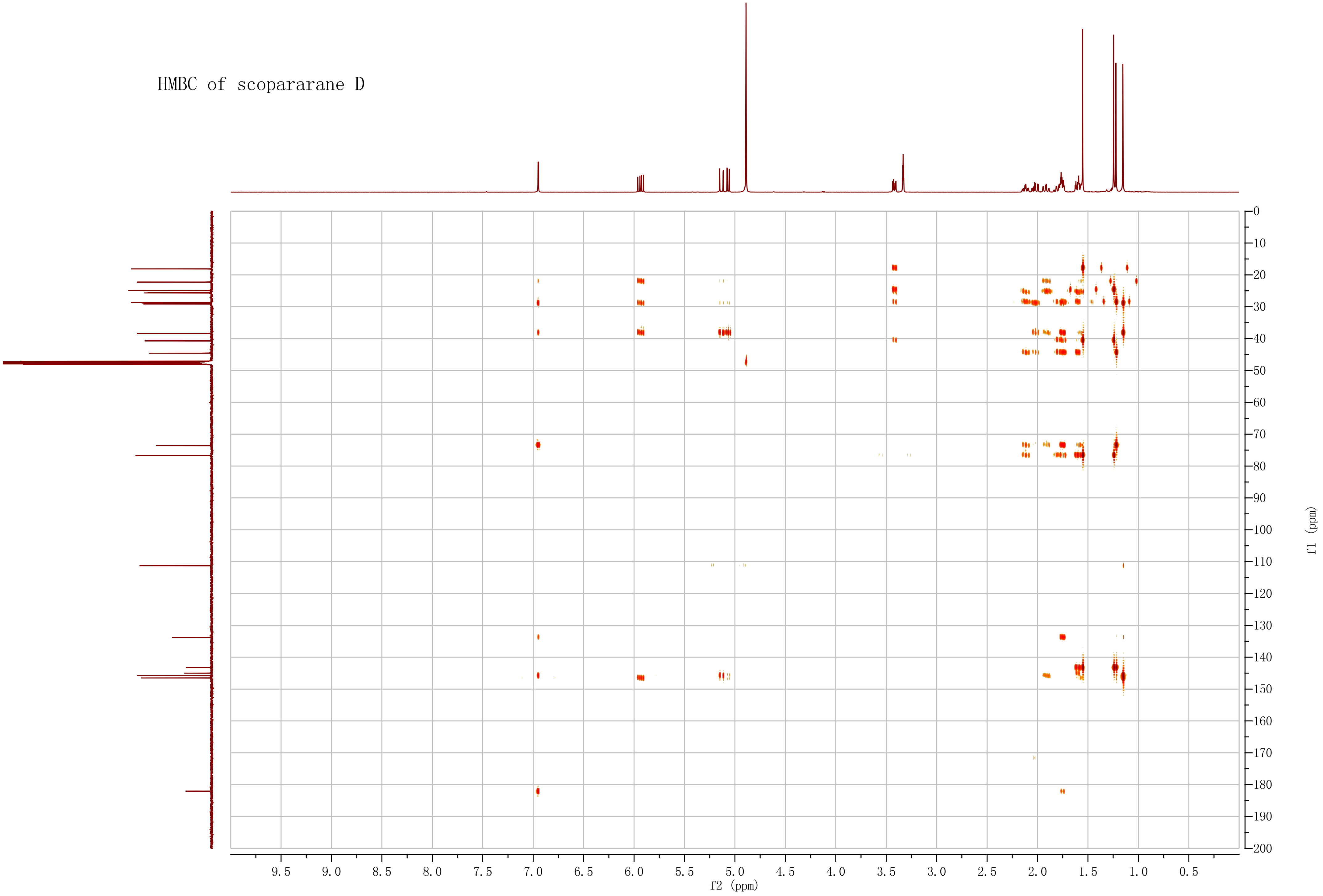

1H-NMR of scopararane E

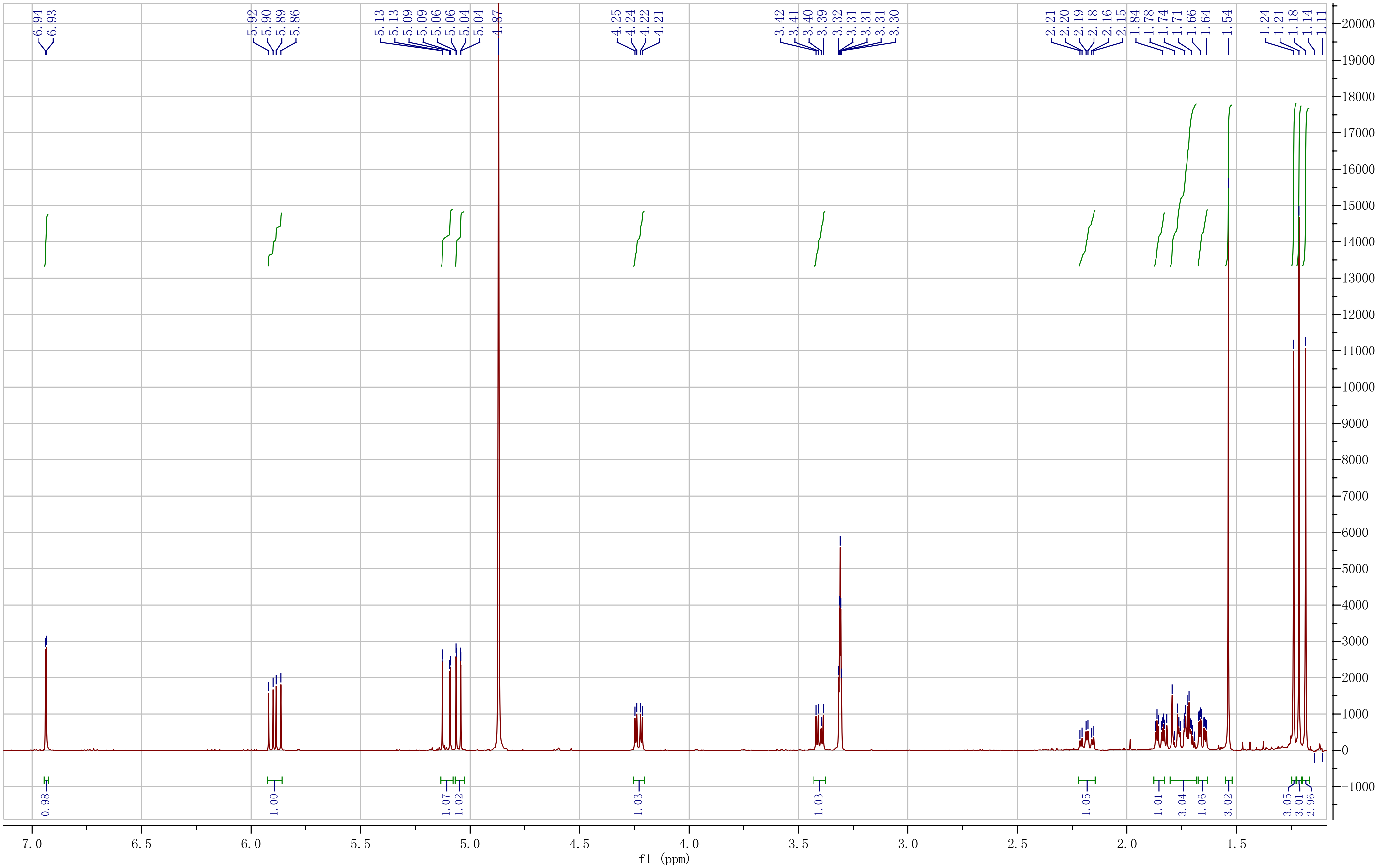

13C-NMR of scopararane E

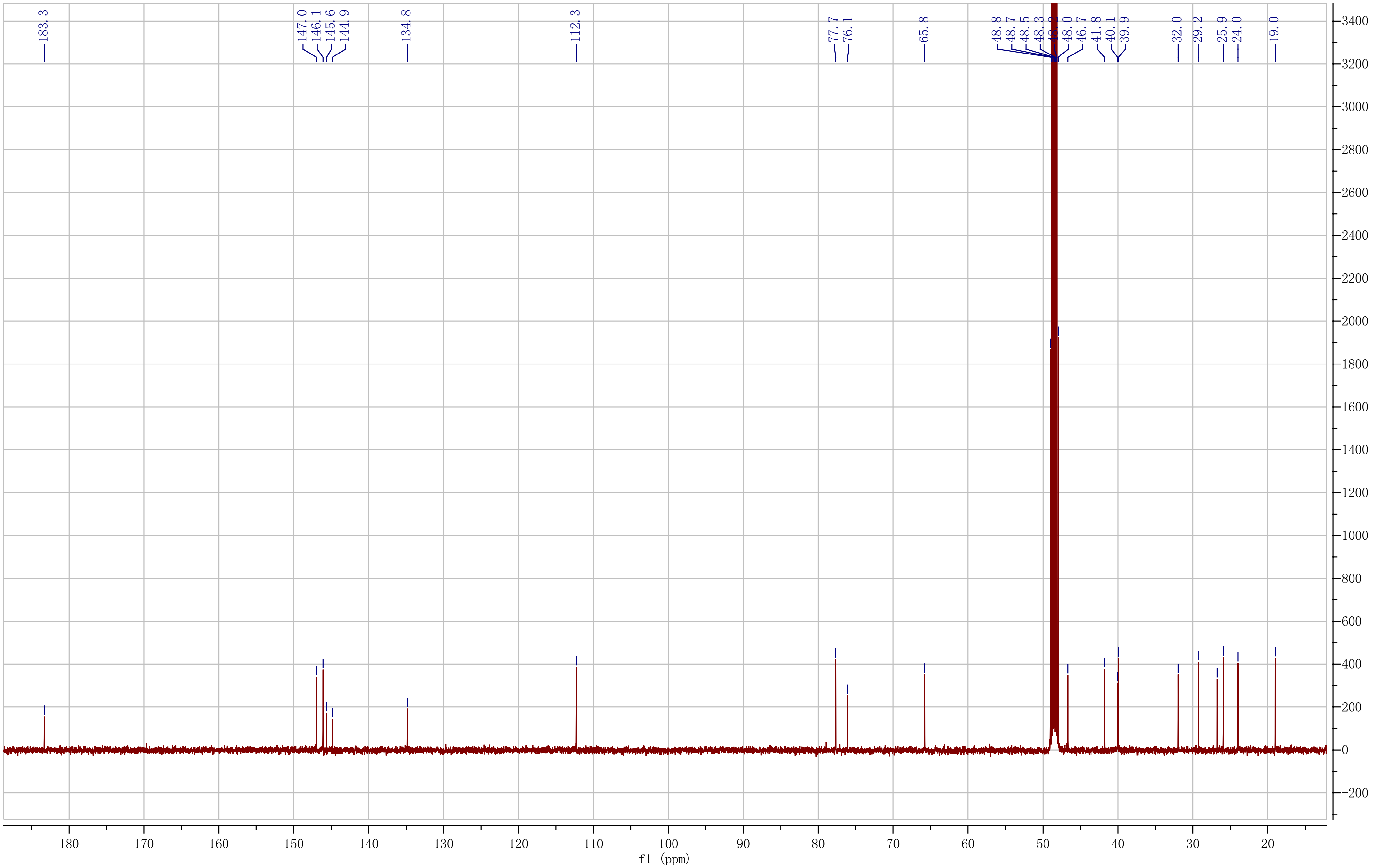

DEPT135 of scopararane E

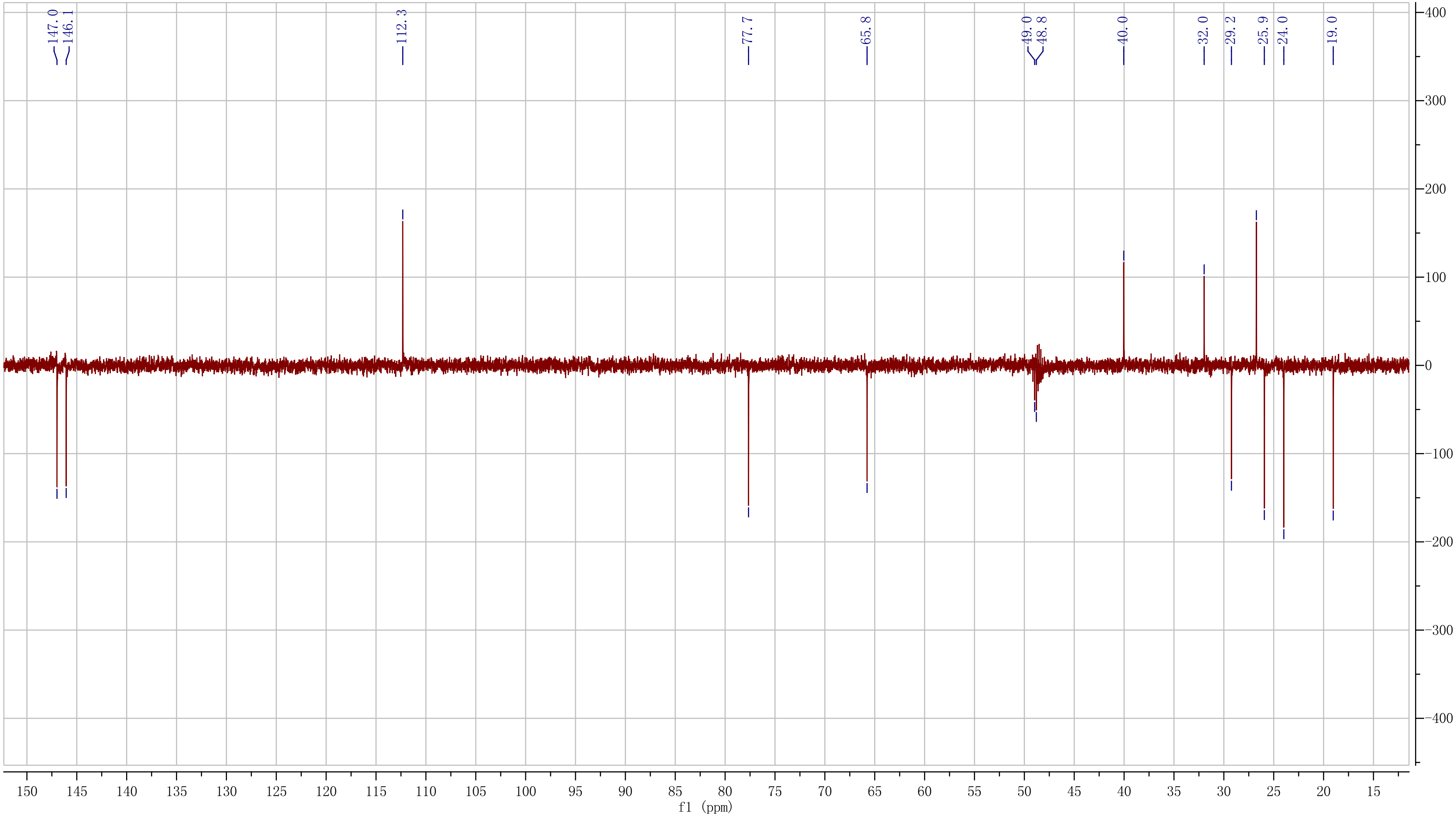

1H-1H COSY of scopararane E

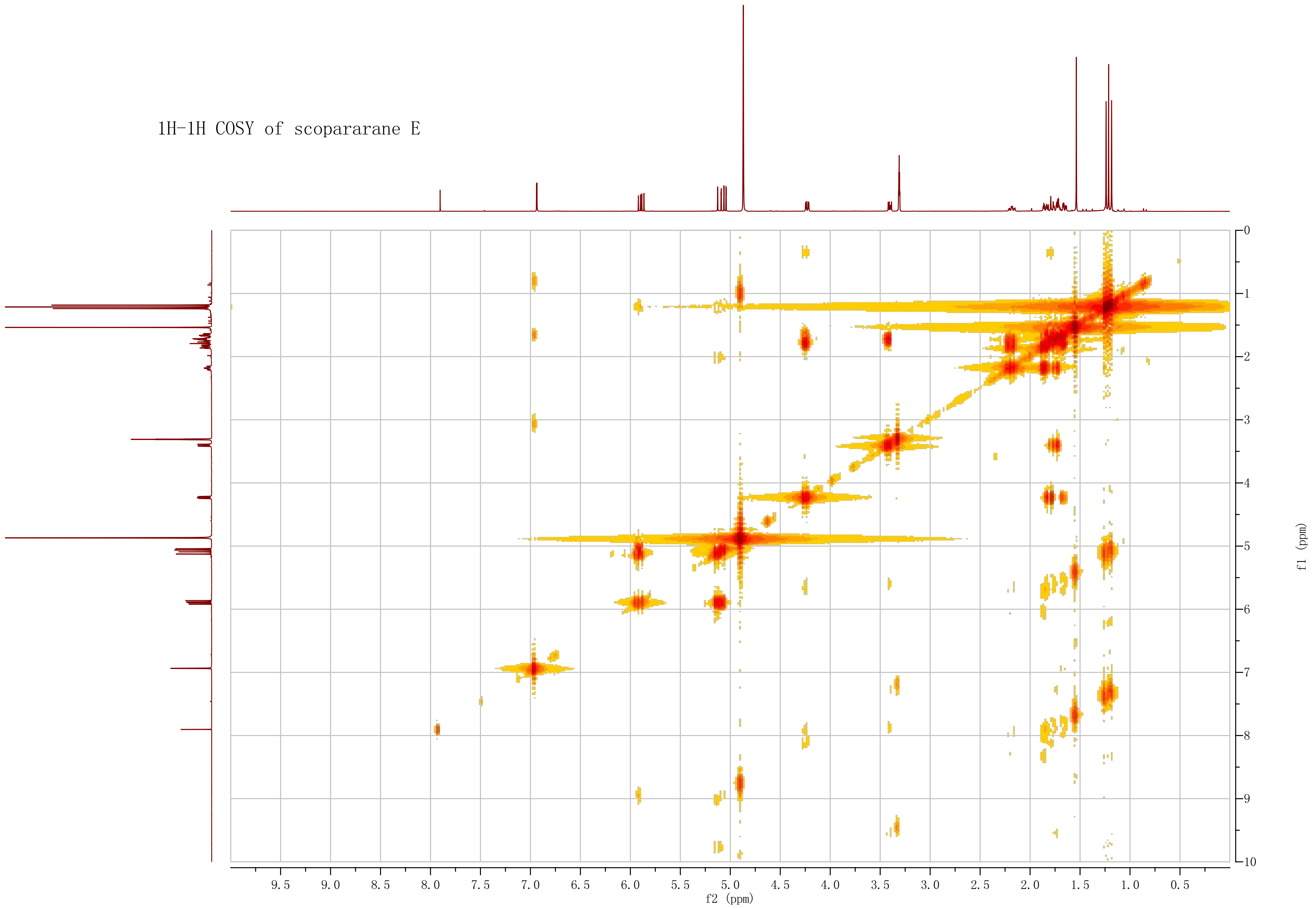

HSQC of scopararane E

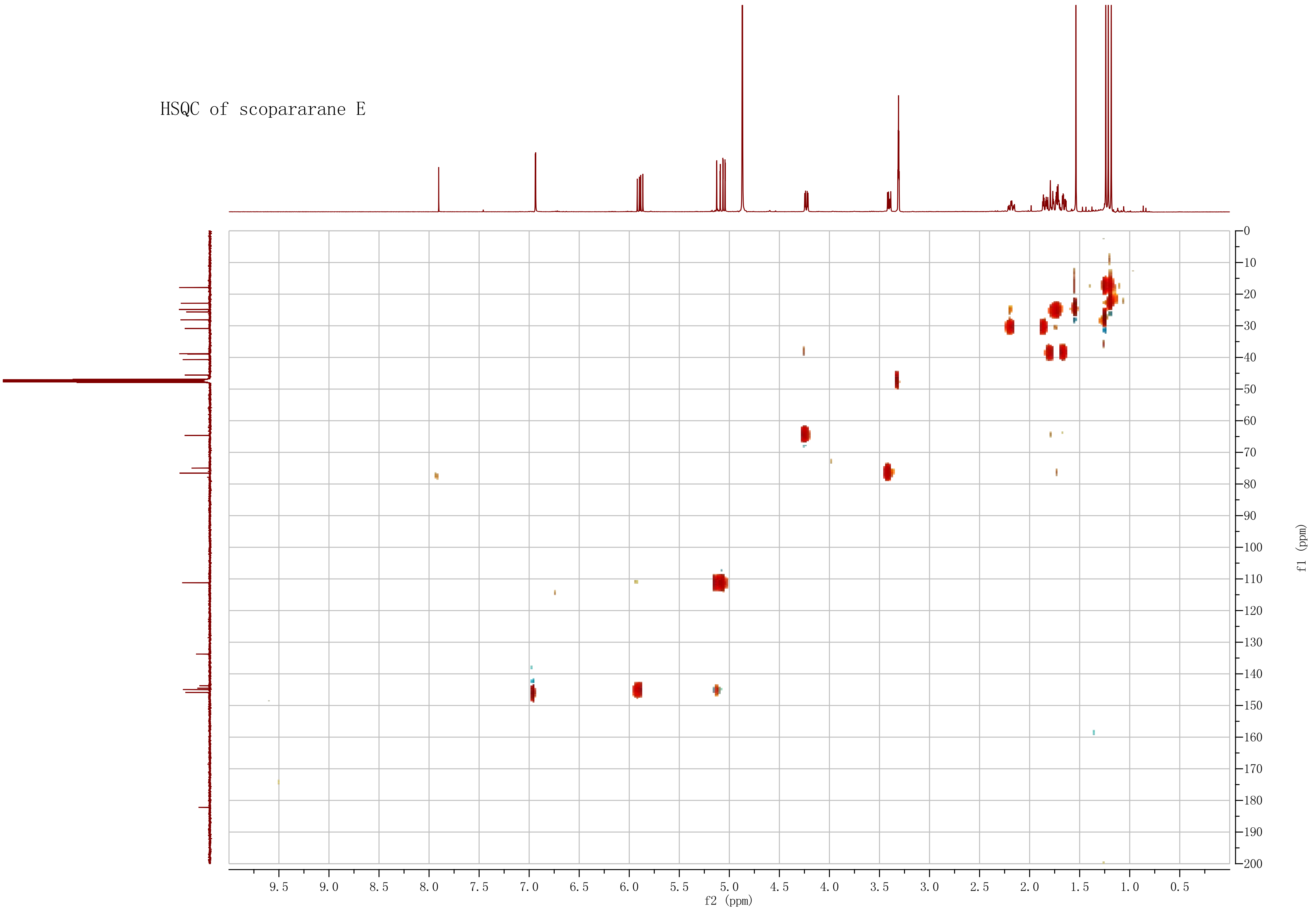

HMBC of scopararane E

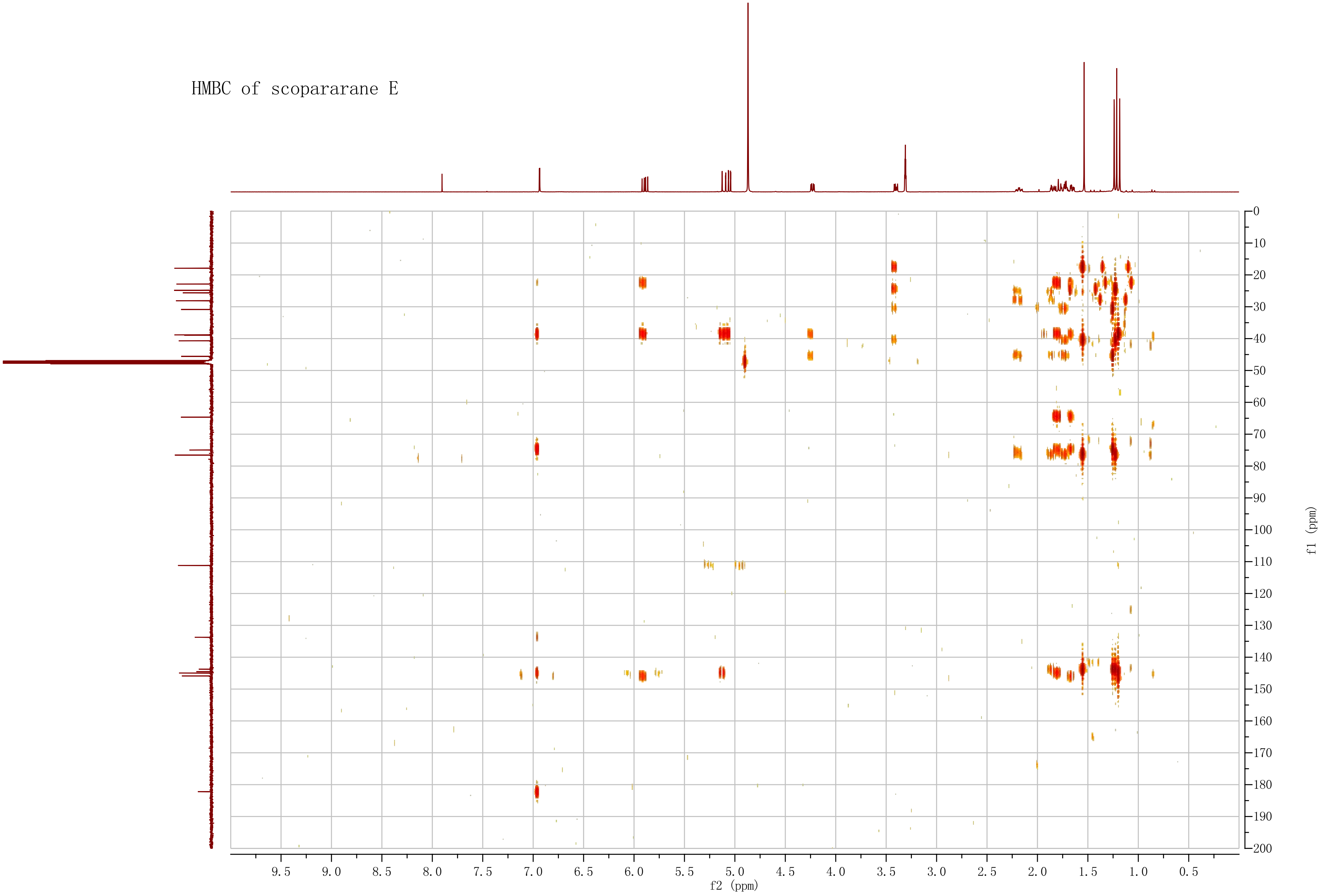

1H-NMR of scopararane F

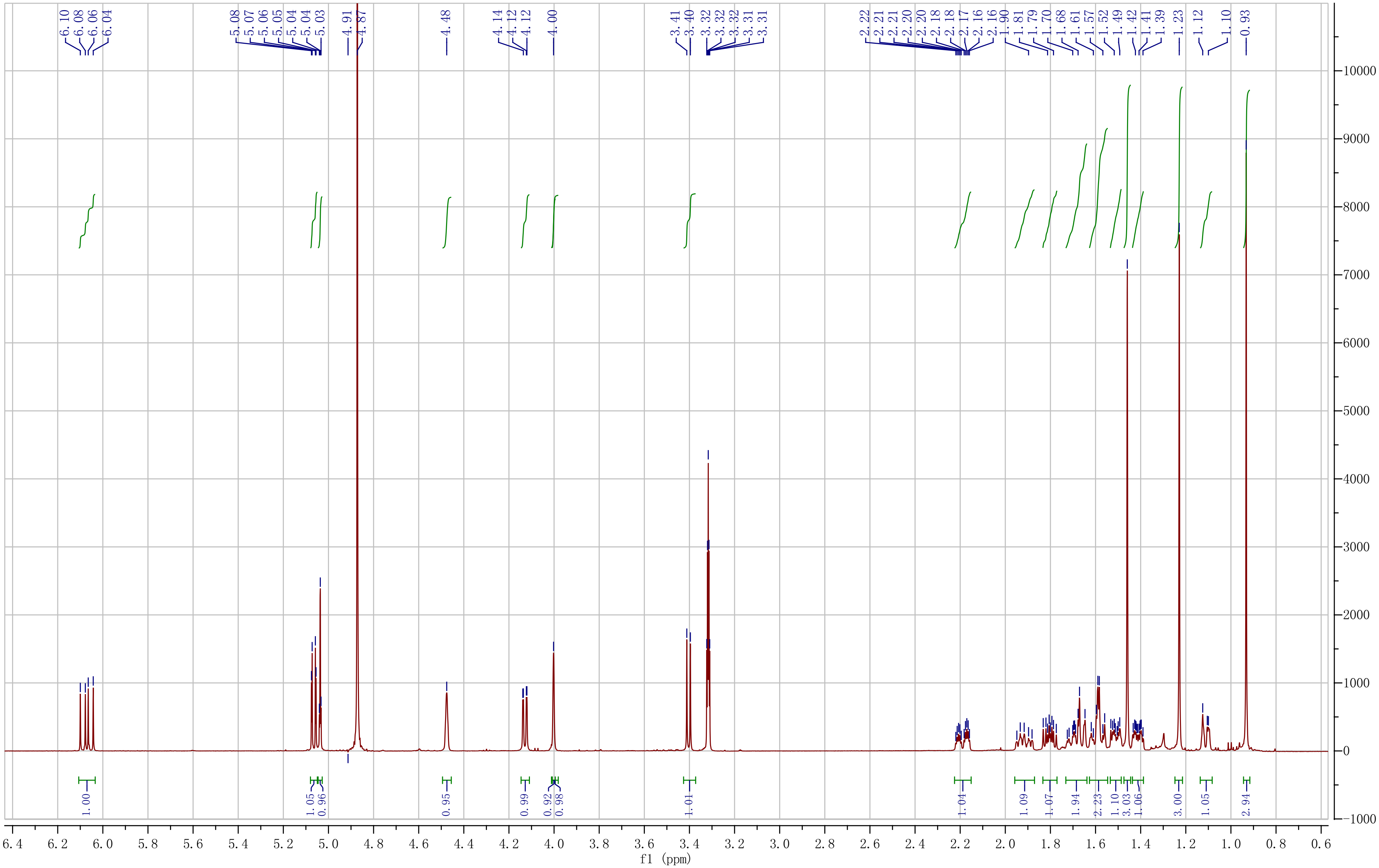

13C-NMR of scopararane F

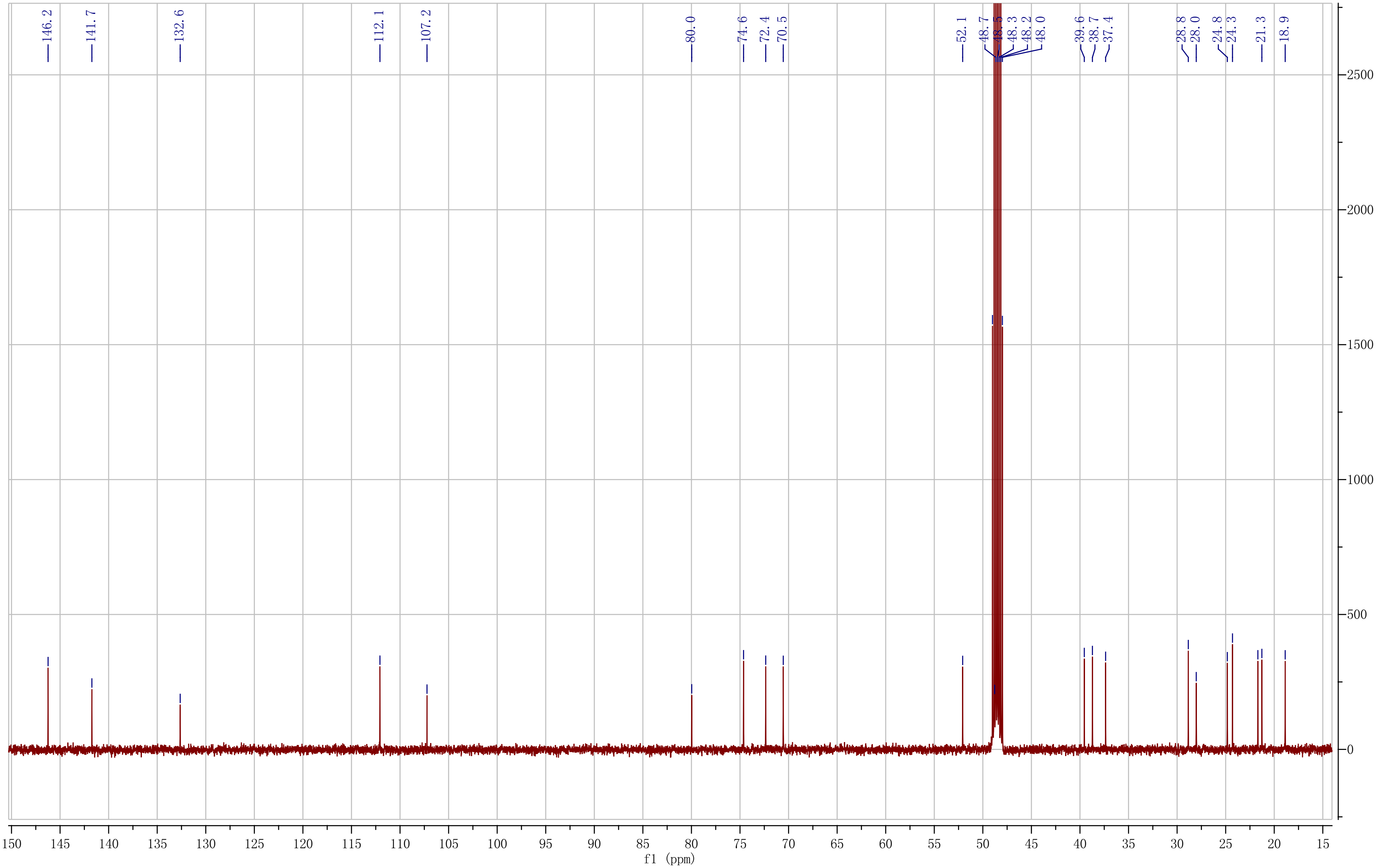

DEPT 135 of scopararane F

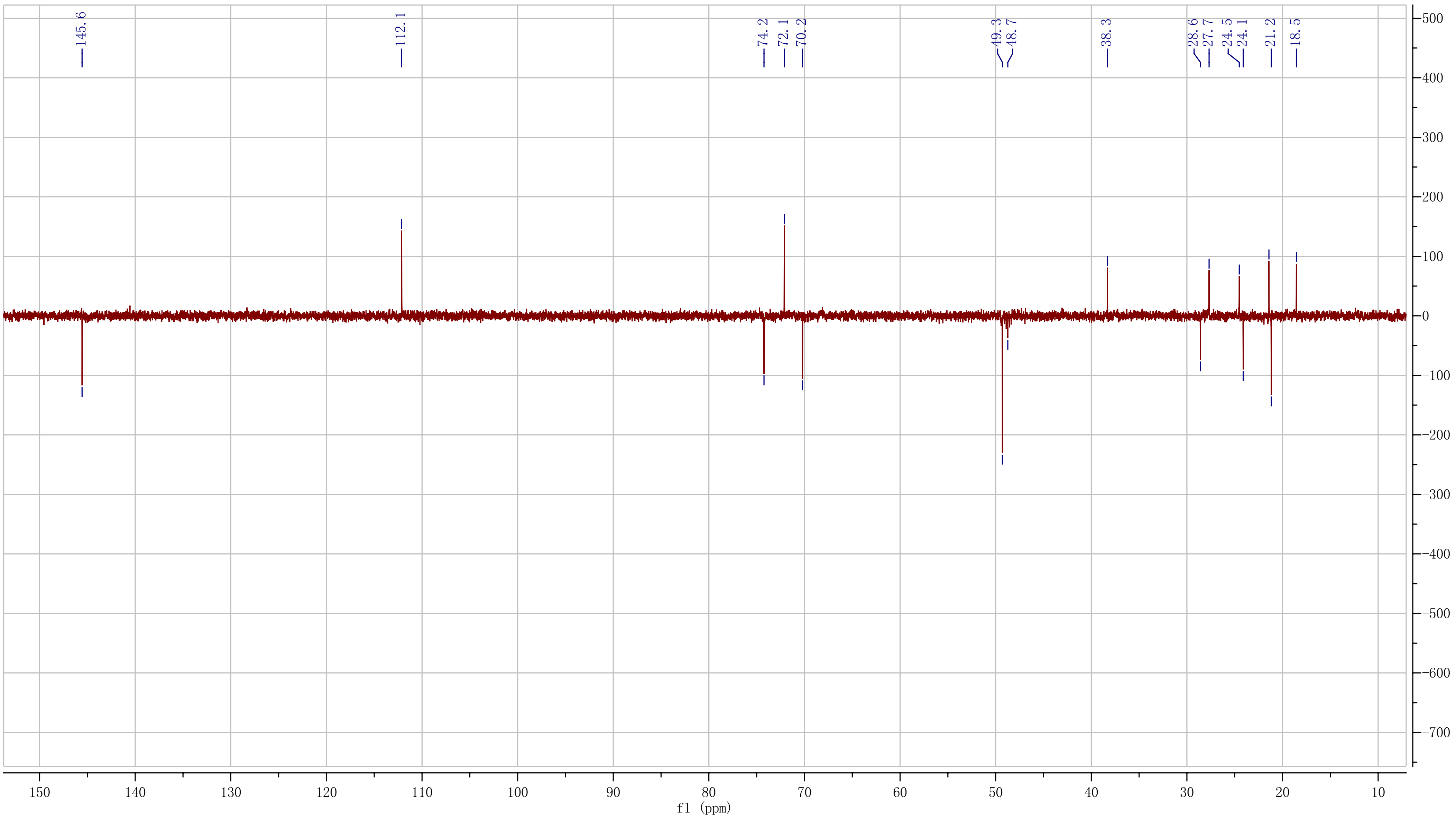

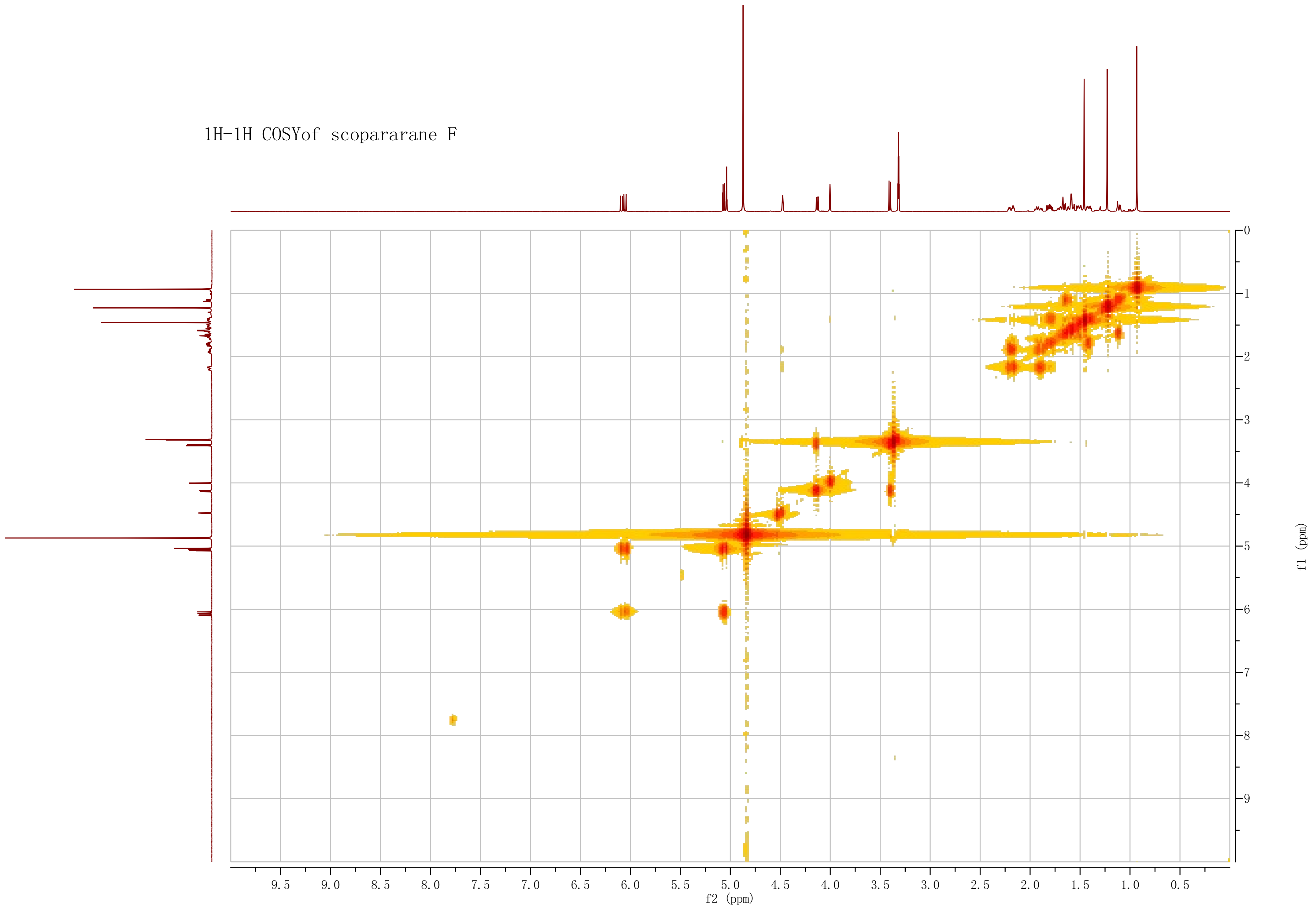
1H-1H COSYof scopararane F
f2 (ppm)
f1 (ppm)
9.5
9.0
8.5
8.0
7.5
7.0
6.5
6.0
5.5
5.0
4.5
4.0
3.5
3.0
2.5
2.0
1.5
1.0
0.5
0
1
2
3
4
5
6
7
8
9

HSQC scopararane F

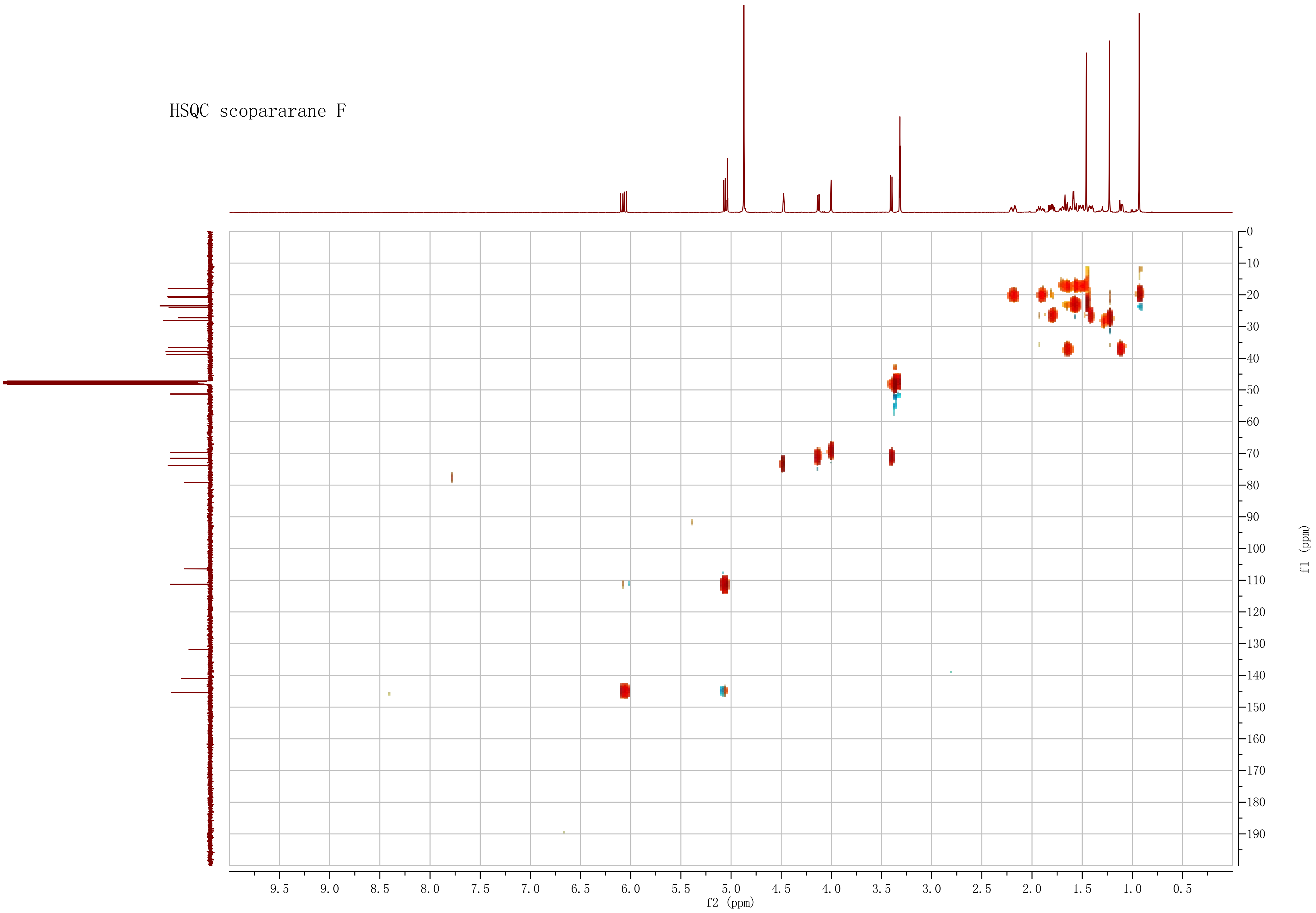

HMBC of scopararane F

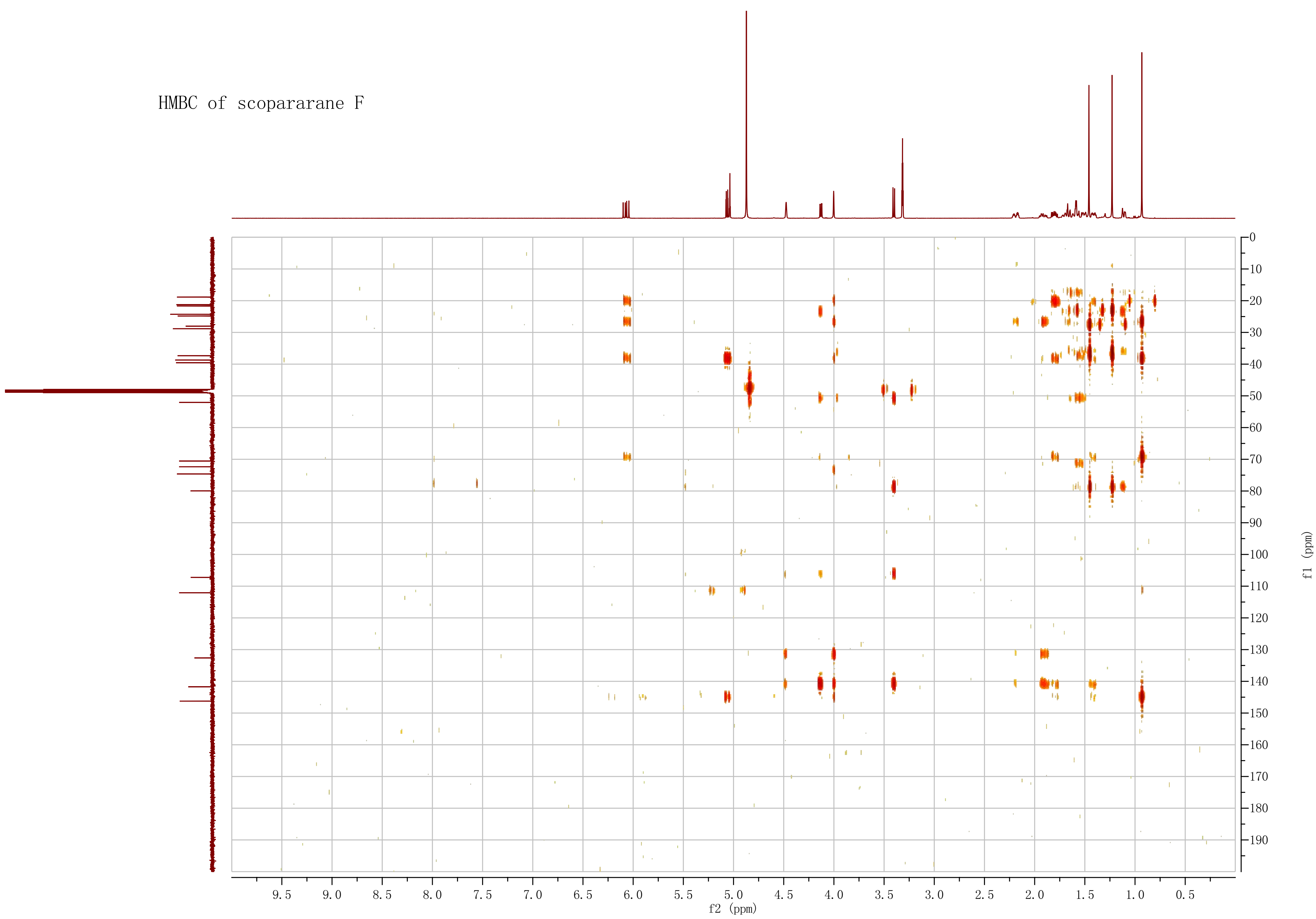

1H-NMR of scopararane G

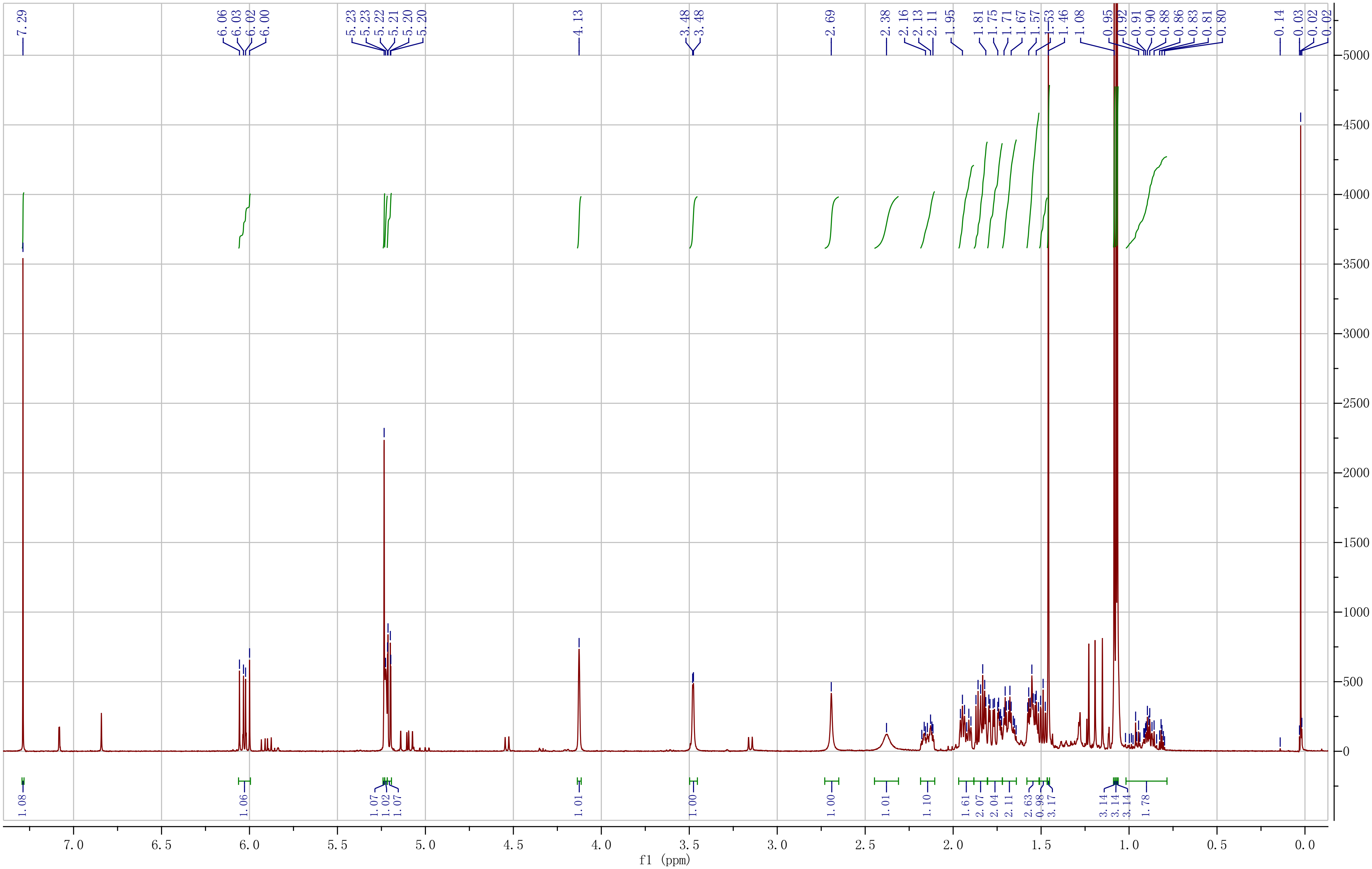

13C-NMR of scopararane G

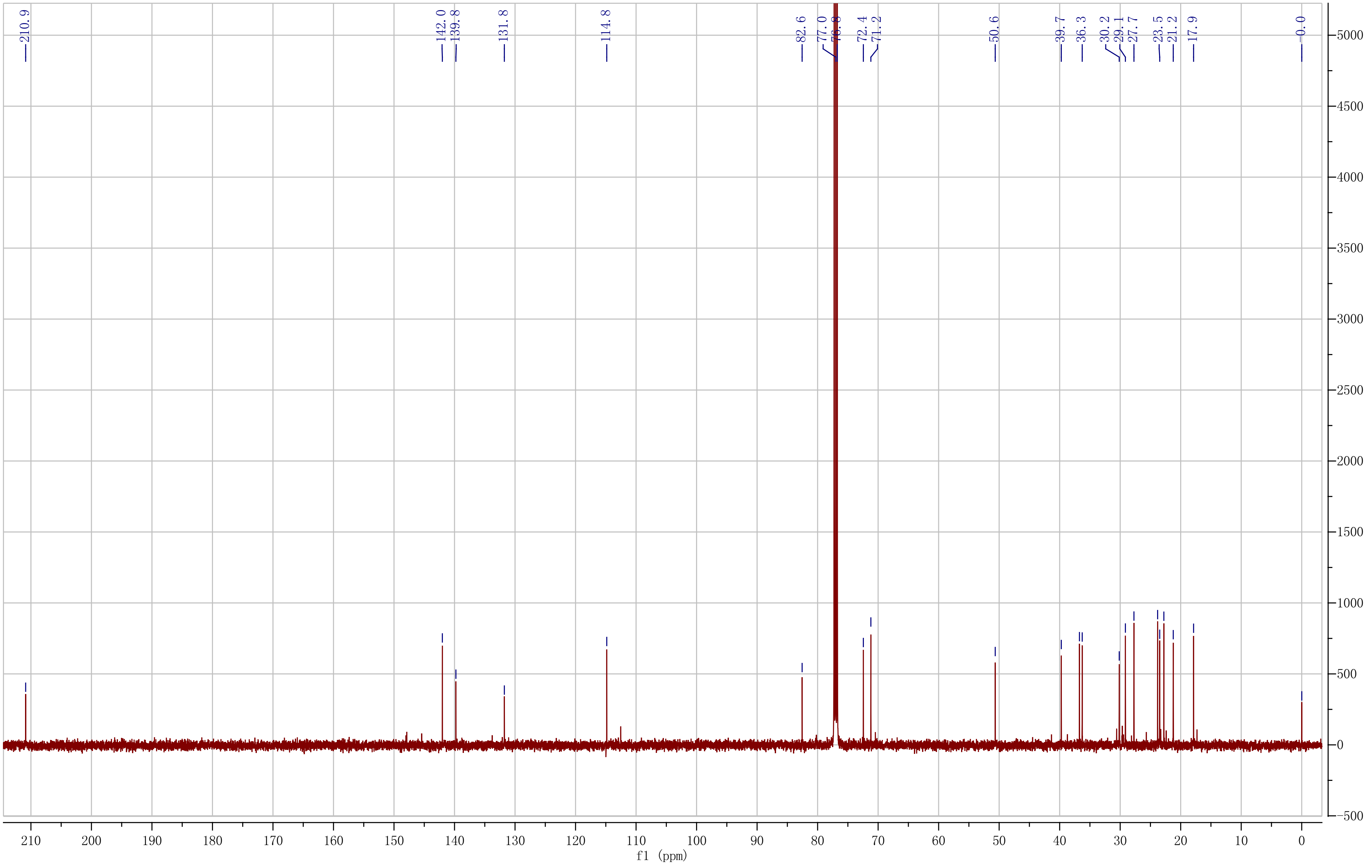

DEPT 135 of scopararane G

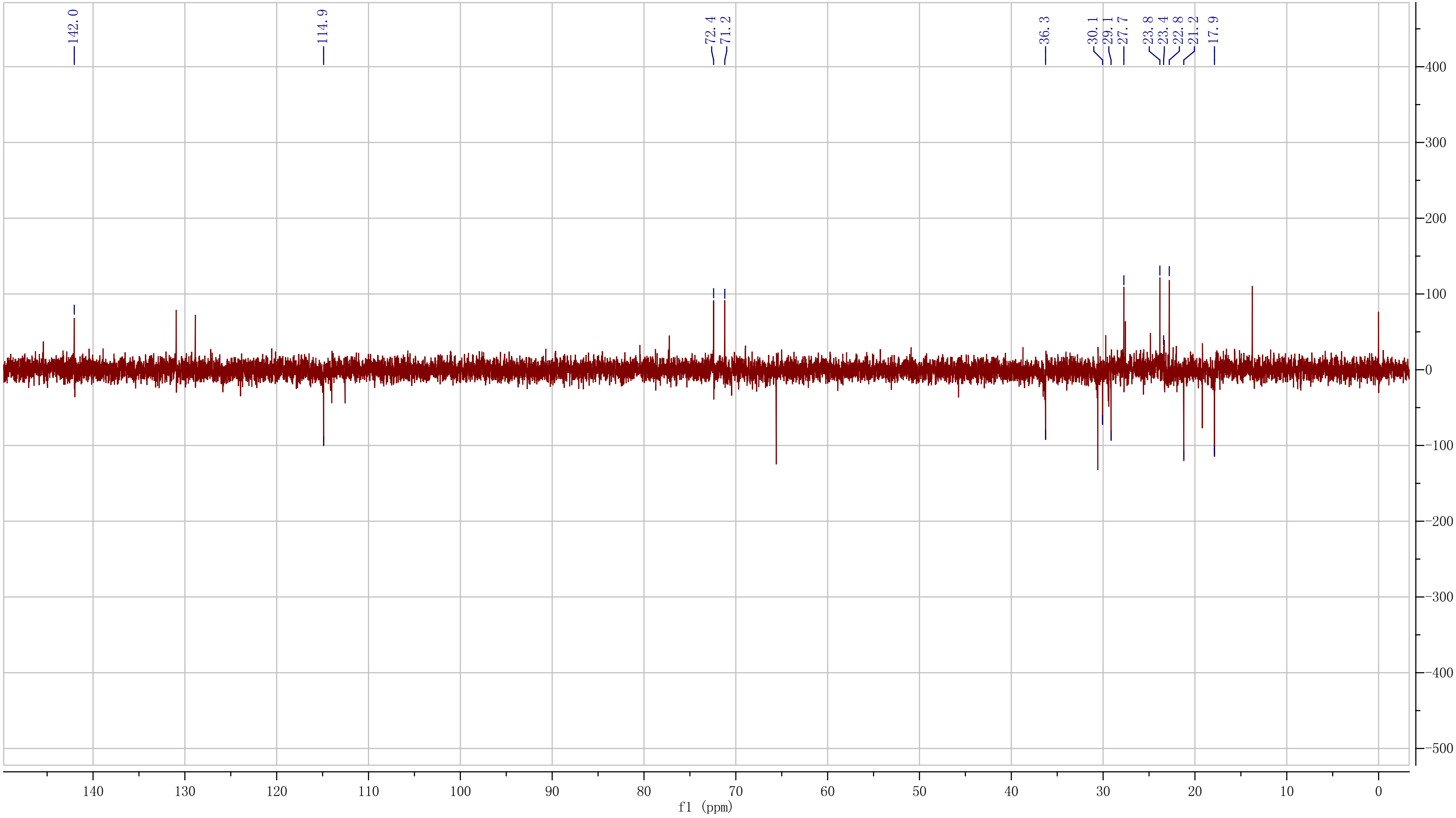

NOESY of scopararane D (1)

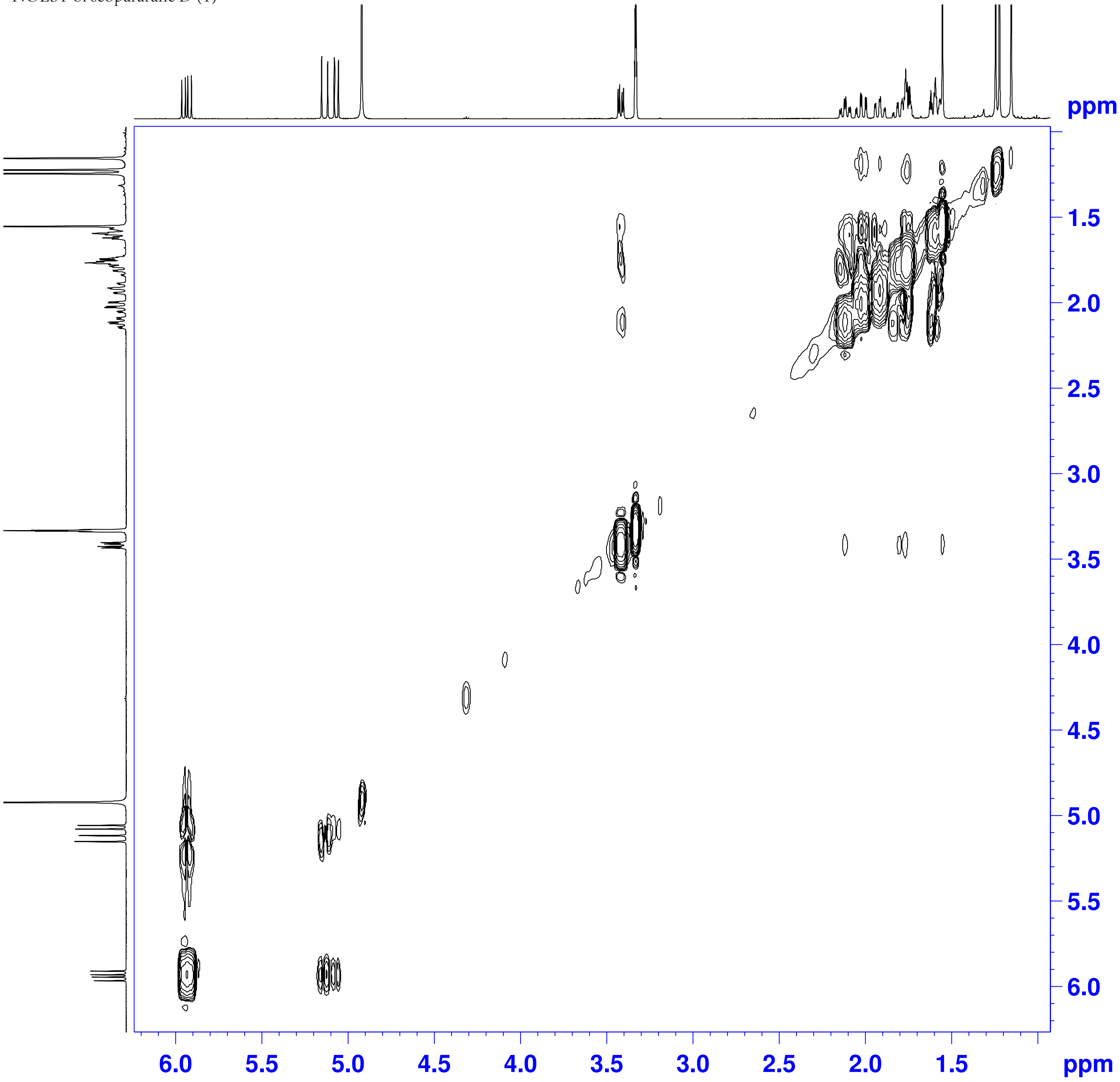

NAME	N24
EXPNO	10
PROCNO	1
Date_	20101214
Time	10.51
INSTRUM	spect
PROBHD	5 mm PABBO BB-
PULPROG	roesygpph19.2
TD	1024
SOLVENT	MeOD
NS	32
DS	16
SWH	5000.000
FIDRES	4.882813
AQ	0.1024500
RG	203
DW	100.000
DE	6.50
TE	293.0
D0	0.00008687
D1	2.00000000
D12	0.00002000
D16	0.00020000
D19	0.00025000
IN0	0.00019995
L4	500
P15	200000.00

======== CHANNEL f1 ====

NUC1	1H
P0	14.30
P1	14.30
P25	200.00
P27	14.30
PL1	2.50
PL18	2.50
PL27	19.39
PL1W	14.12537575
PL18W	14.12537575
PL27W	0.28906804
SFO1	500.1325007

====== GRADIENT CHANNEL

GPNAM1	SINE.100
GPNAM2	SINE.100
GPZ1	20.00
GPZ2	20.00
P16	1000.00
ND0	1
TD	128
SFO1	500.1325
FIDRES	39.072853
SW	10.000
FnMODE	States-TPPI
SI	1024
SF	500.1299996
WDW	QSINE
SSB	2
LB	0.00

NOESY of scopararane D (2)

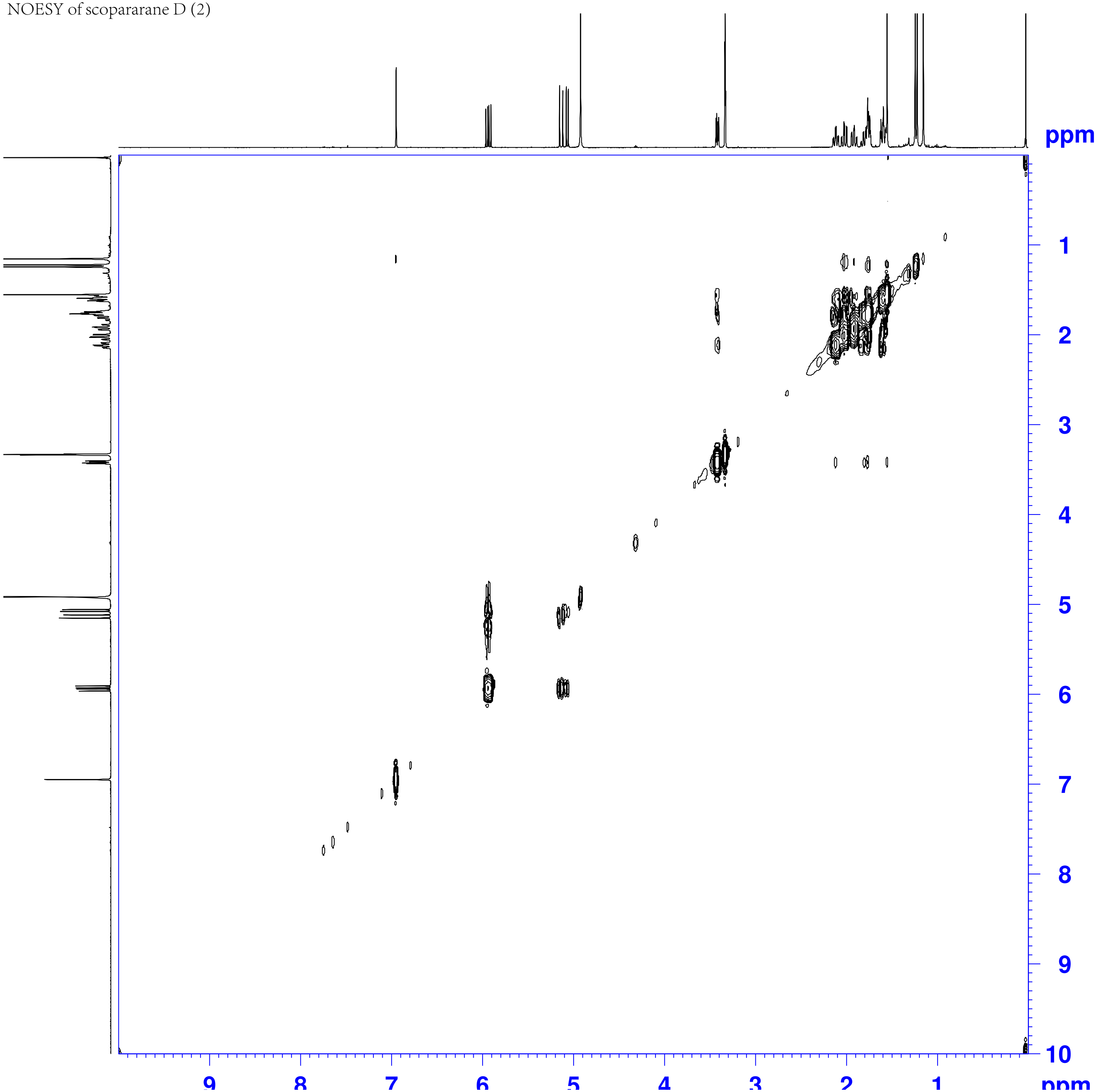

NAME	N24
EXPNO	10
PROCNO	1
Date_	20101214
Time	10.51
INSTRUM	spect
PROBHD	5 mm PABBO BB-
PULPROG	roesygpph19.2
TD	1024
SOLVENT	MeOD
NS	32
DS	16
SWH	5000.000
FIDRES	4.882813
AQ	0.1024500
RG	203
DW	100.000
DE	6.50
TE	293.0
D0	0.00008687
D1	2.00000000
D12	0.00002000
D16	0.00020000
D19	0.00025000
IN0	0.00019995
L4	500
P15	200000.00
======== CHANNEL f1 ====	
NUC1	1H
P0	14.30
P1	14.30
P25	200.00
P27	14.30
PL1	2.50
PL18	2.50
PL27	19.39
PL1W	14.12537575
PL18W	14.12537575
PL27W	0.28906804
SFO1	500.1325007
====== GRADIENT CHANNEL	
GPNAM1	SINE.100
GPNAM2	SINE.100
GPZ1	20.00
GPZ2	20.00
P16	1000.00
ND0	1
TD	128
SFO1	500.1325
FIDRES	39.072853
SW	10.000
FnMODE	States-TPPI
SI	1024
SF	500.1299996
WDW	QSINE
SSB	2
LB	0.00

NOESY of scopararane E (1)

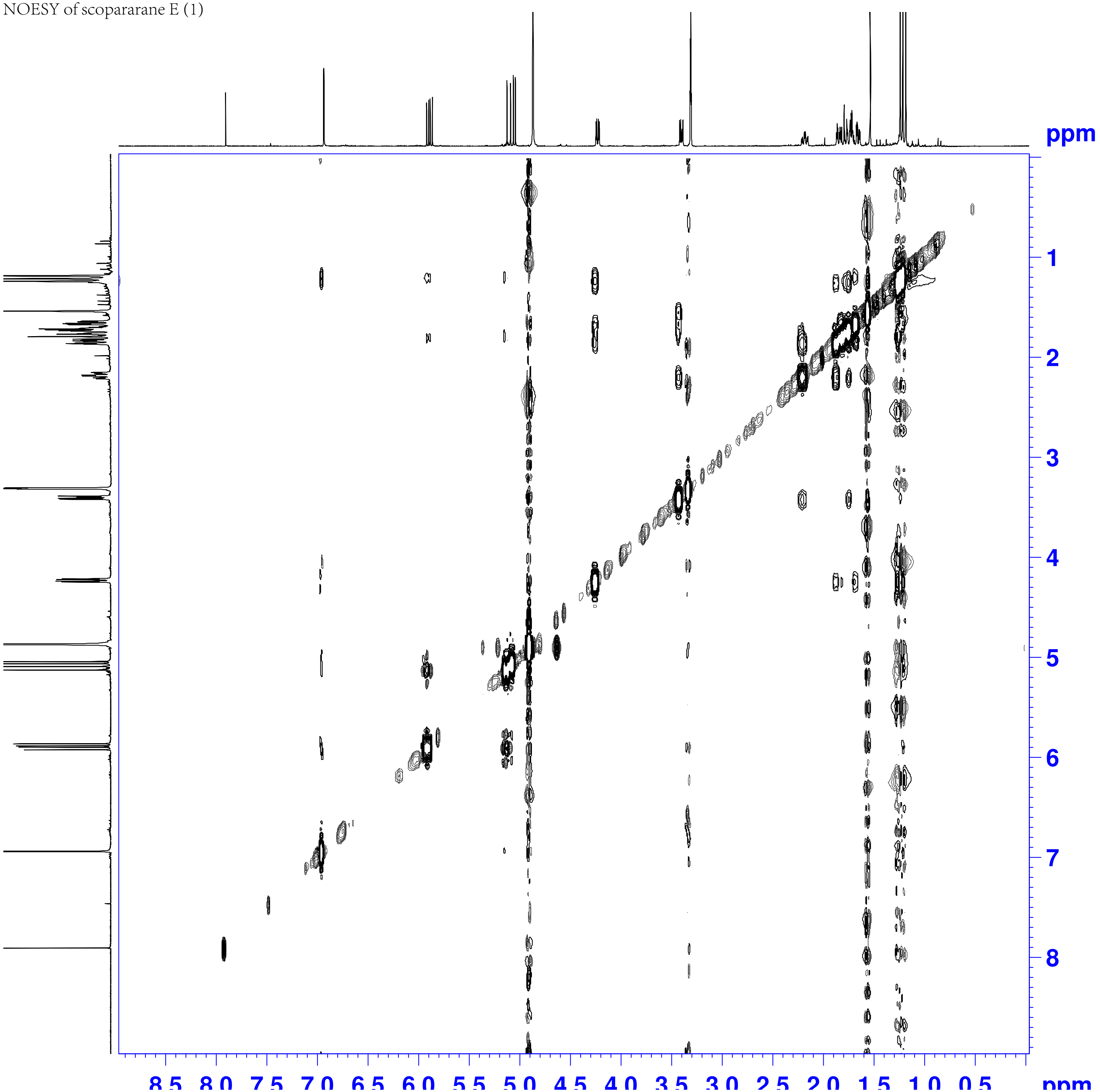

NAME	P23a2
EXPNO	8
PROCNO	1
Date_	20101022
Time	9.53
INSTRUM	spect
PROBHD	5 mm PABBO BB-
PULPROG	noesygpph
TD	1024
SOLVENT	MeOD
NS	8
DS	16
SWH	4504.504
FIDRES	4.398930
AQ	0.1137140
RG	64
DW	111.000
DE	6.50
TE	295.0
D0	0.00009287
D1	2.00000000
D8	0.50000000
D16	0.00020000
IN0	0.00022215
======== CHANNEL f1 ====	
NUC1	1H
P1	14.30
P2	28.60
PL1	2.50
PL1W	14.12537575
SFO1	500.1322506
====== GRADIENT CHANNEL	
GPNAM1	SINE.100
GPNAM2	SINE.100
GPZ1	20.00
GPZ2	20.00
P16	1000.00
ND0	1
TD	128
SFO1	500.1323
FIDRES	35.165550
SW	9.000
FnMODE	States-TPPI
SI	1024
SF	500.1300000
WDW	QSINE
SSB	2
LB	0.00
GB	0
PC	1.40
SI	1024
MC2	States-TPPI
SF	500.1300000
WDW	QSINE
SSB	2
LB	0.00
GB	0

NOESY of scopararane E (2)

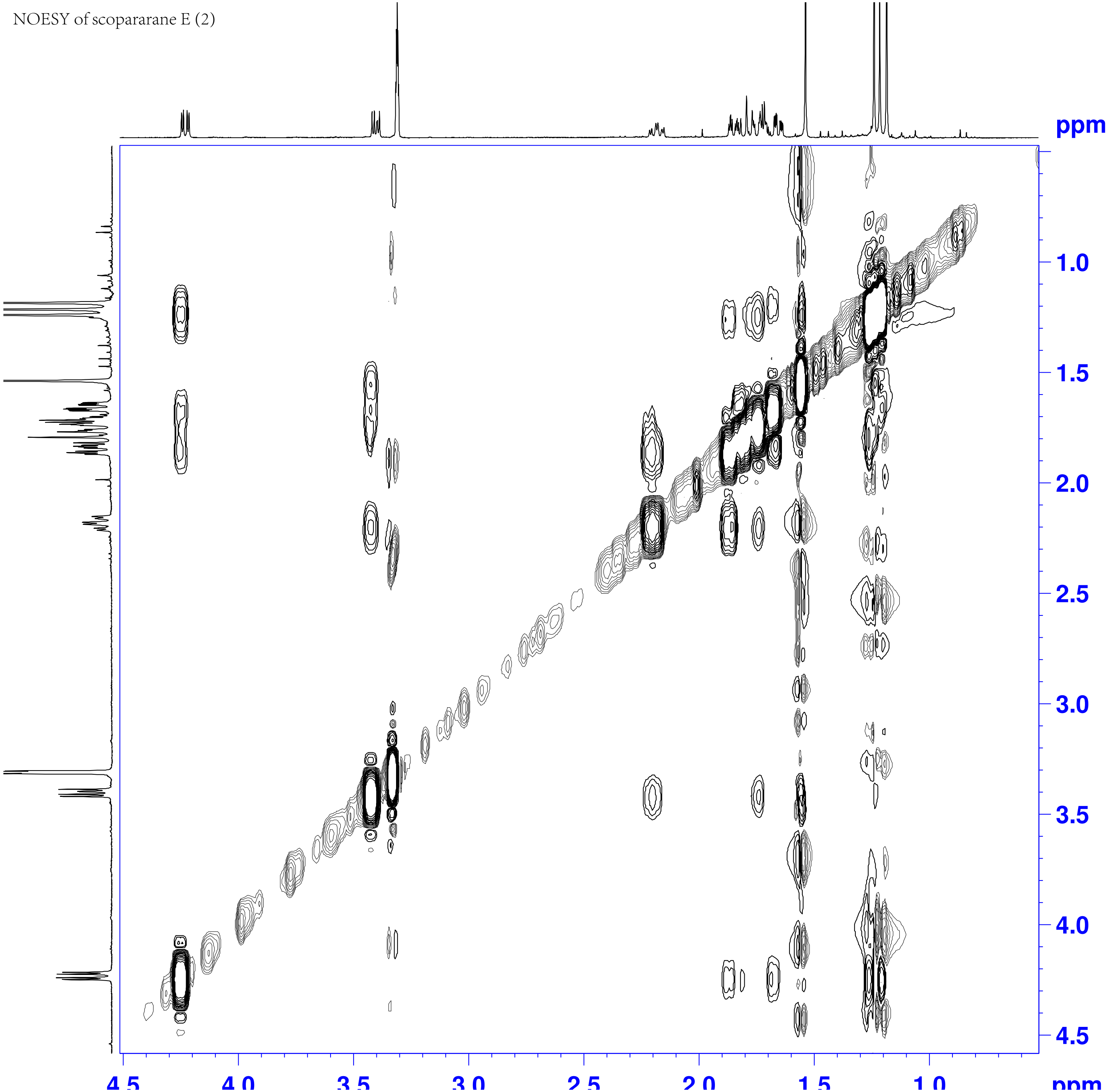

NAME	P23a2
EXPNO	8
PROCNO	1
Date_	20101022
Time	9.53
INSTRUM	spect
PROBHD	5 mm PABBO BB-
PULPROG	noesygpph
TD	1024
SOLVENT	MeOD
NS	8
DS	16
SWH	4504.504
FIDRES	4.398930
AQ	0.1137140
RG	64
DW	111.000
DE	6.50
TE	295.0
D0	0.00009287
D1	2.00000000
D8	0.50000000
D16	0.00020000
IN0	0.00022215

======== CHANNEL f1 ====

NUC1	1H
P1	14.30
P2	28.60
PL1	2.50
PL1W	14.12537575
SFO1	500.1322506

====== GRADIENT CHANNEL

GPNAM1	SINE.100
GPNAM2	SINE.100
GPZ1	20.00
GPZ2	20.00
P16	1000.00
ND0	1
TD	128
SFO1	500.1323
FIDRES	35.165550
SW	9.000
FnMODE	States-TPPI
SI	1024
SF	500.1300000
WDW	QSINE
SSB	2
LB	0.00
GB	0
PC	1.40
SI	1024
MC2	States-TPPI
SF	500.1300000
WDW	QSINE
SSB	2
LB	0.00
GB	0

NOESY of scopararane F (1)

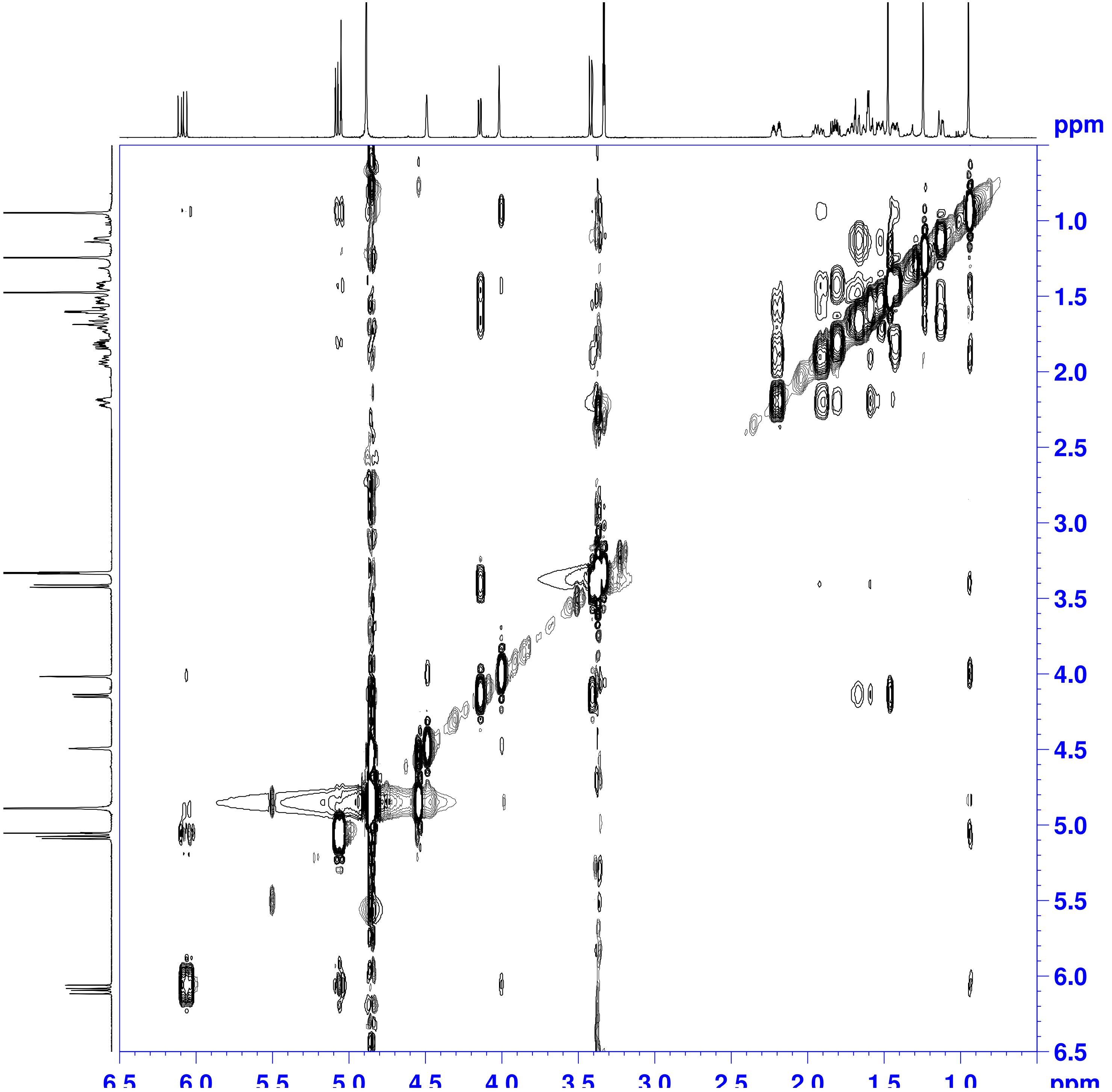

NAME	S021
EXPNO	8
PROCNO	1
Date_	20101022
Time	10.52
INSTRUM	spect
PROBHD	5 mm PABBO BB-
PULPROG	noesygpph
TD	1024
SOLVENT	MeOD
NS	8
DS	16
SWH	4504.504
FIDRES	4.398930
AQ	0.1137140
RG	64
DW	111.000
DE	6.50
TE	295.1
D0	0.00009287
D1	2.00000000
D8	0.50000000
D16	0.00020000
IN0	0.00022215

======== CHANNEL f1 ====

NUC1	1H
P1	14.30
P2	28.60
PL1	2.50
PL1W	14.12537575
SFO1	500.1322506

====== GRADIENT CHANNEL

GPNAM1	SINE.100
GPNAM2	SINE.100
GPZ1	20.00
GPZ2	20.00
P16	1000.00
ND0	1
TD	128
SFO1	500.1323
FIDRES	35.165550
SW	9.000
FnMODE	States-TPPI
SI	1024
SF	500.1300000
WDW	QSINE
SSB	2
LB	0.00
GB	0
PC	1.40
SI	1024
MC2	States-TPPI
SF	500.1300000
WDW	QSINE
SSB	2
LB	0.00
GB	0

NOESY of scopararane F (2)

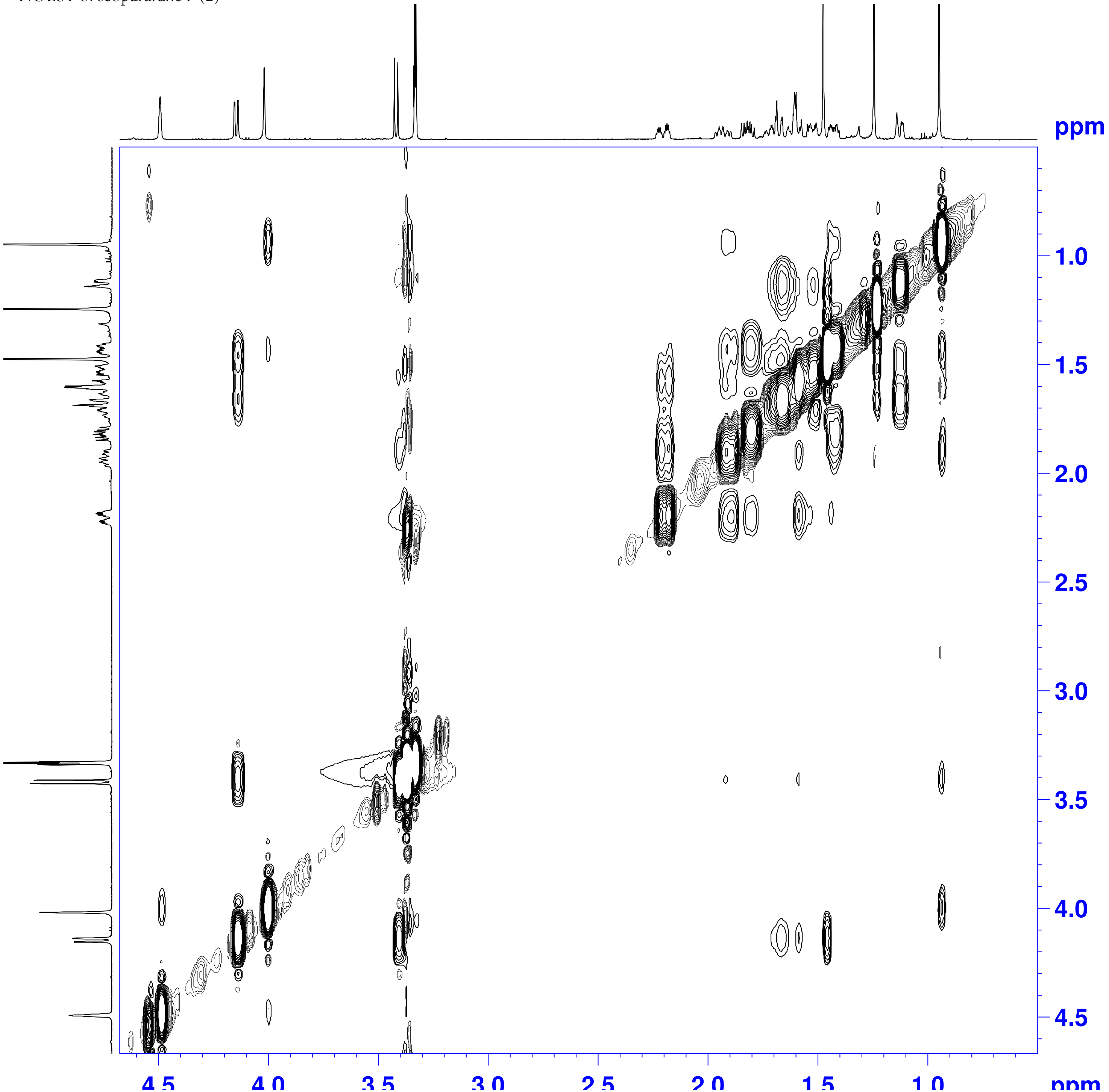

NAME	S021
EXPNO	8
PROCNO	1
Date_	20101022
Time	10.52
INSTRUM	spect
PROBHD	5 mm PABBO BB-
PULPROG	noesygpph
TD	1024
SOLVENT	MeOD
NS	8
DS	16
SWH	4504.504
FIDRES	4.398930
AQ	0.1137140
RG	64
DW	111.000
DE	6.50
TE	295.1
D0	0.00009287
D1	2.00000000
D8	0.50000000
D16	0.00020000
IN0	0.00022215

======== CHANNEL f1 ====

NUC1	1H
P1	14.30
P2	28.60
PL1	2.50
PL1W	14.12537575
SFO1	500.1322506

====== GRADIENT CHANNEL

GPNAM1	SINE.100
GPNAM2	SINE.100
GPZ1	20.00
GPZ2	20.00
P16	1000.00
ND0	1
TD	128
SFO1	500.1323
FIDRES	35.165550
SW	9.000
FnMODE	States-TPPI
SI	1024
SF	500.1300000
WDW	QSINE
SSB	2
LB	0.00
GB	0
PC	1.40
SI	1024
MC2	States-TPPI
SF	500.1300000
WDW	QSINE
SSB	2
LB	0.00
GB	0

NOESY of scopararane F (3)

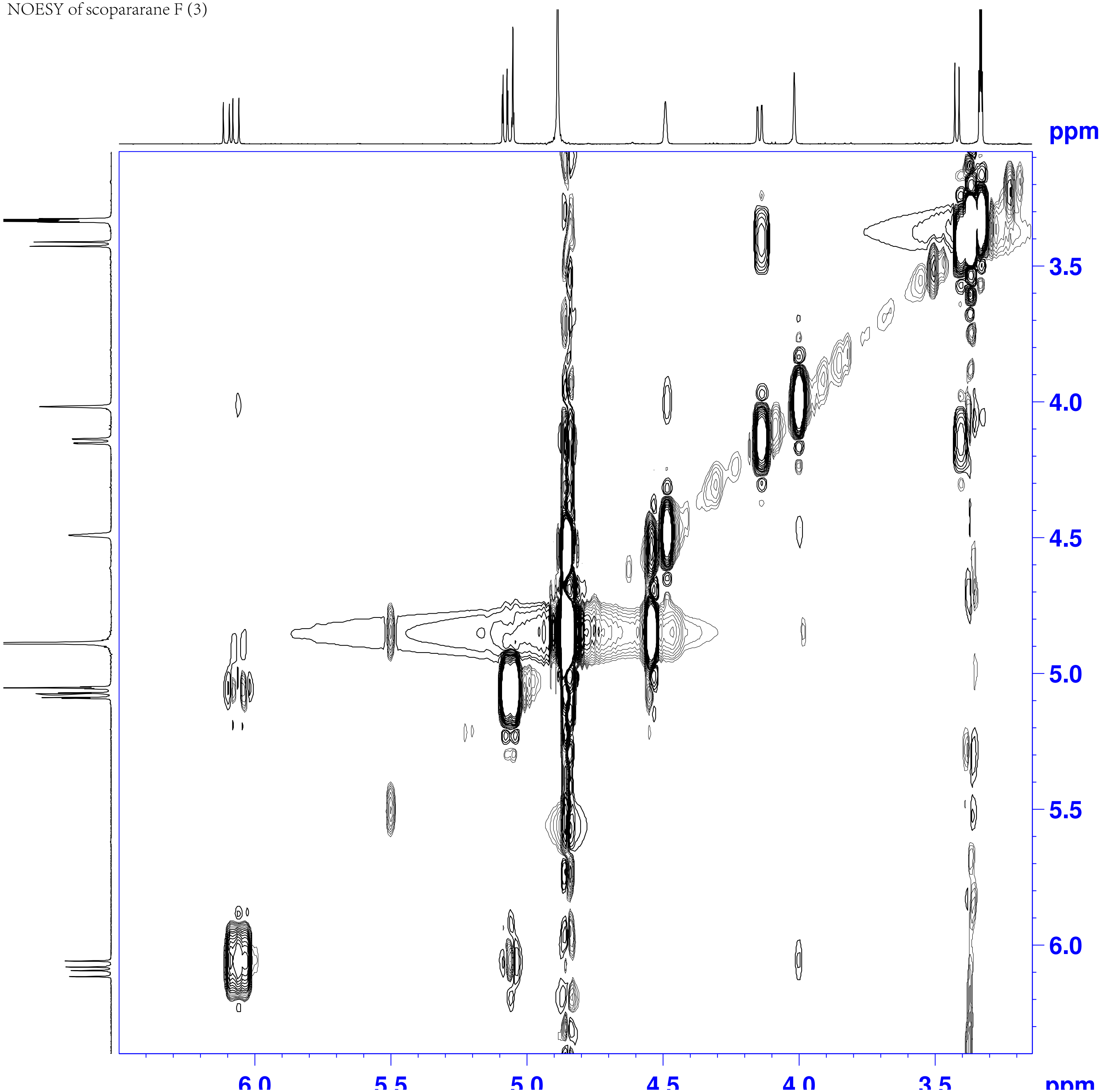

NAME	S021
EXPNO	8
PROCNO	1
Date_	20101022
Time	10.52
INSTRUM	spect
PROBHD	5 mm PABBO BB-
PULPROG	noesygpph
TD	1024
SOLVENT	MeOD
NS	8
DS	16
SWH	4504.504
FIDRES	4.398930
AQ	0.1137140
RG	64
DW	111.000
DE	6.50
TE	295.1
D0	0.00009287
D1	2.00000000
D8	0.50000000
D16	0.00020000
IN0	0.00022215
======== CHANNEL f1 ====	
NUC1	1H
P1	14.30
P2	28.60
PL1	2.50
PL1W	14.12537575
SFO1	500.1322506
====== GRADIENT CHANNEL	
GPNAM1	SINE.100
GPNAM2	SINE.100
GPZ1	20.00
GPZ2	20.00
P16	1000.00
ND0	1
TD	128
SFO1	500.1323
FIDRES	35.165550
SW	9.000
FnMODE	States-TPPI
SI	1024
SF	500.1300000
WDW	QSINE
SSB	2
LB	0.00
GB	0
PC	1.40
SI	1024
MC2	States-TPPI
SF	500.1300000
WDW	QSINE
SSB	2
LB	0.00
GB	0

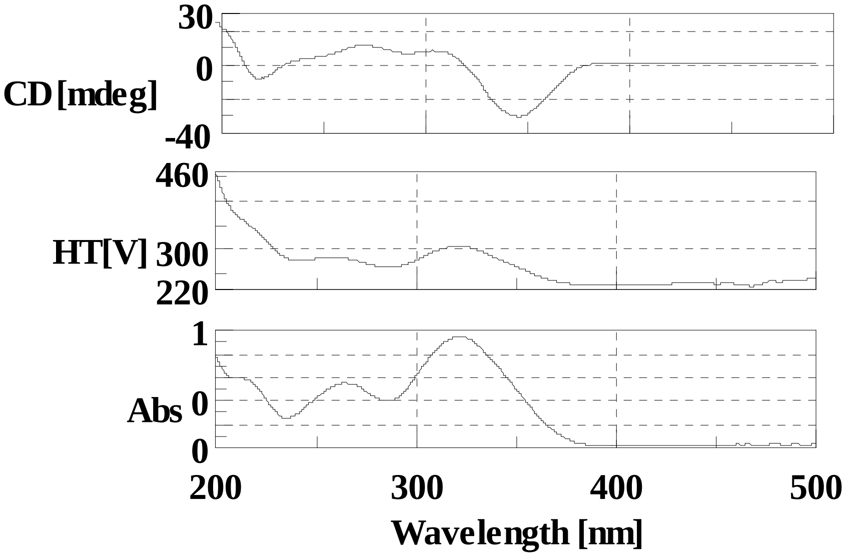

CD of scopararane C (1)

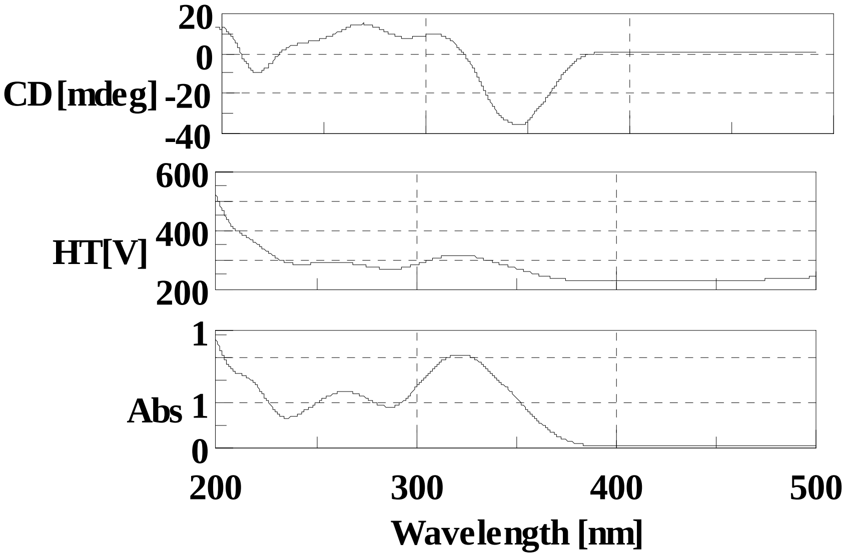

CD of scopararane D (2)

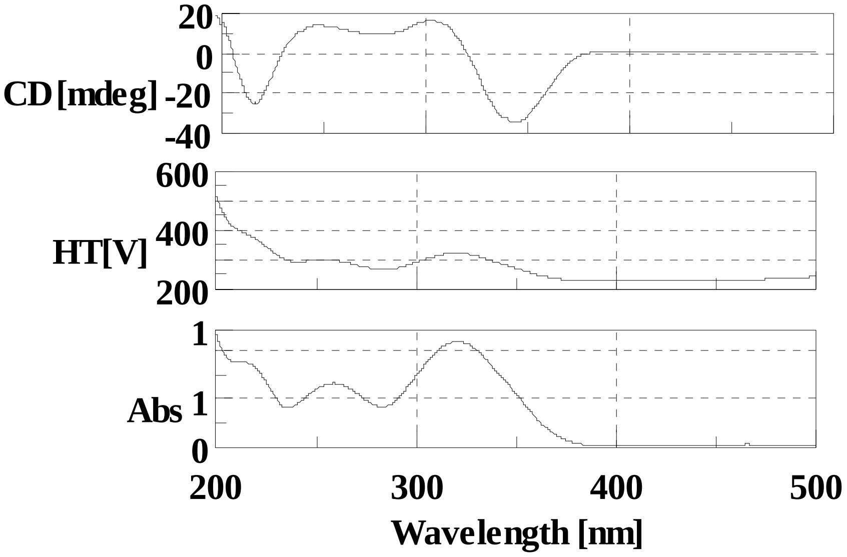

CD of scopararane E (**3**)

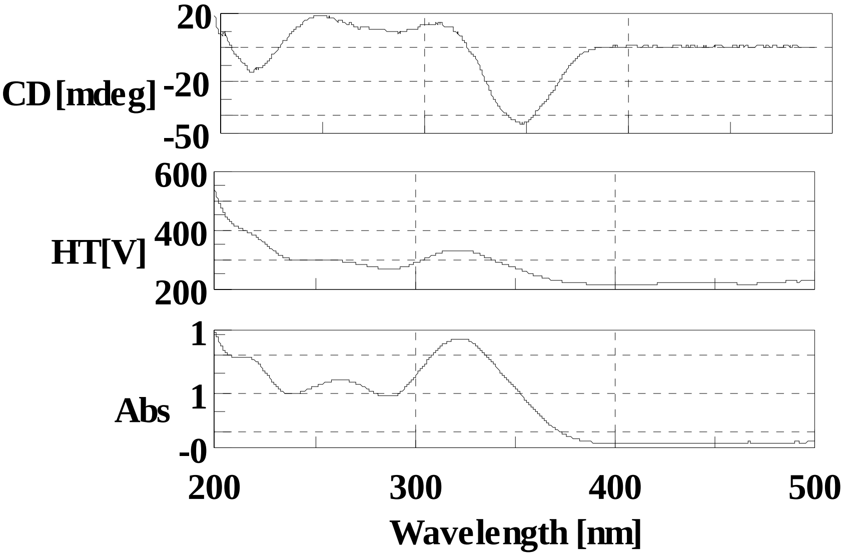

CD of **Libertellenone A (6)**

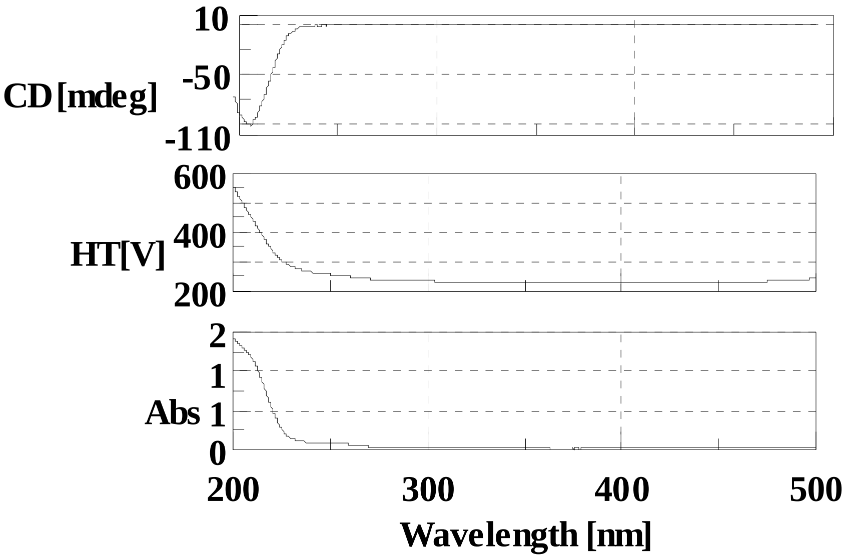

CD of scopararane F (**4**)

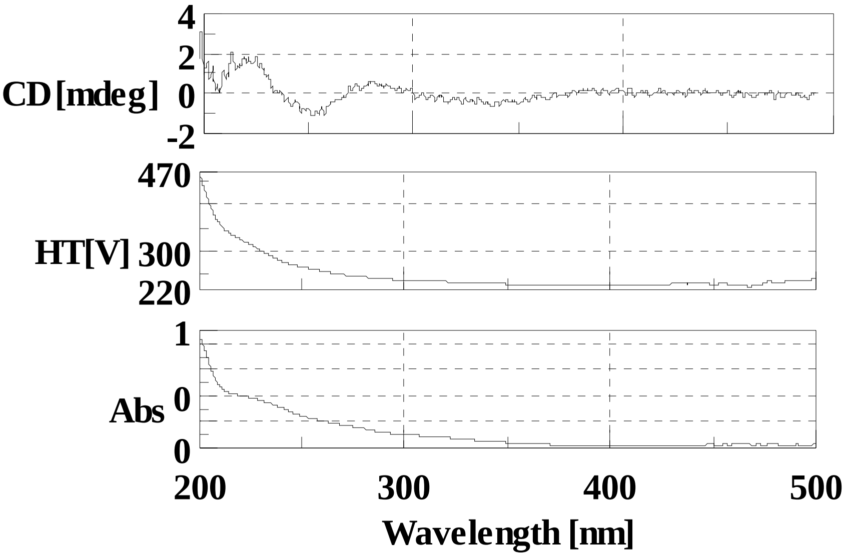

CD of scopararane G (**5**)

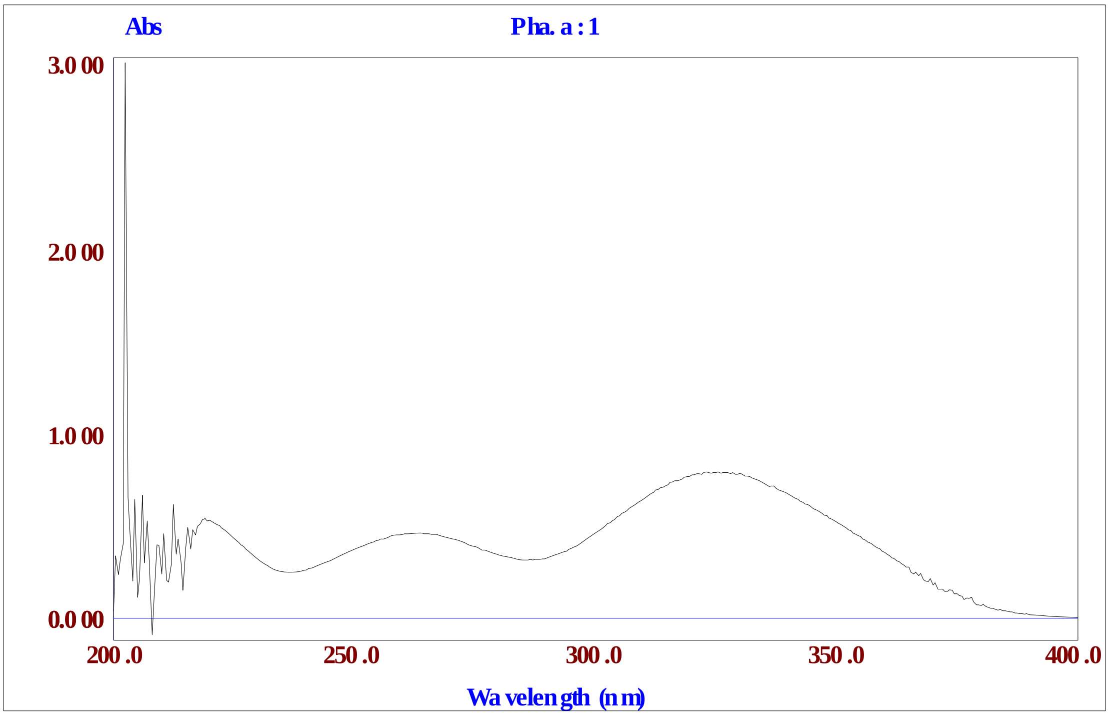

UV of scopararane C (**1**)

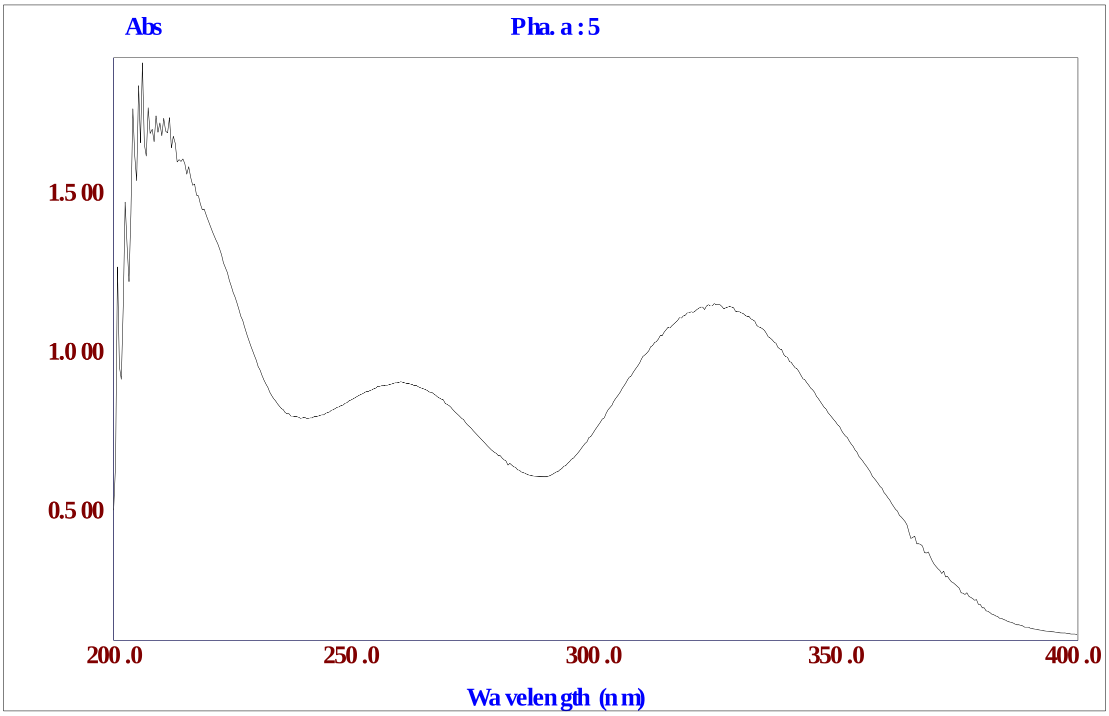

UV of scopararane D (**2**)

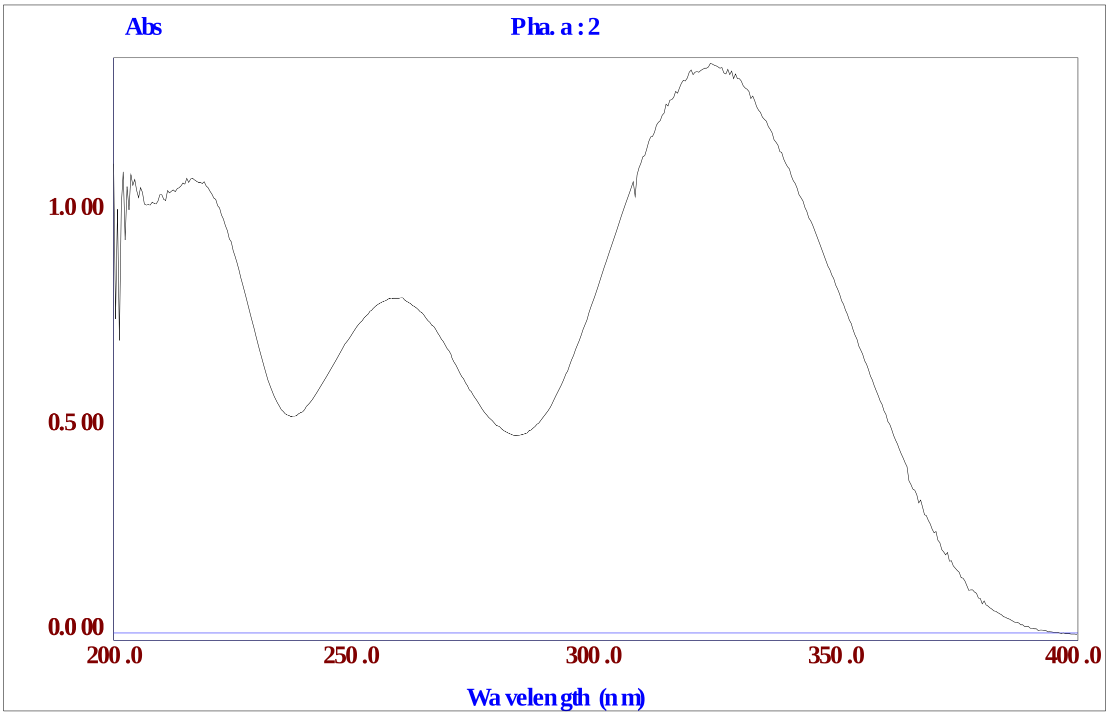

UV of scopararane E (**3**)

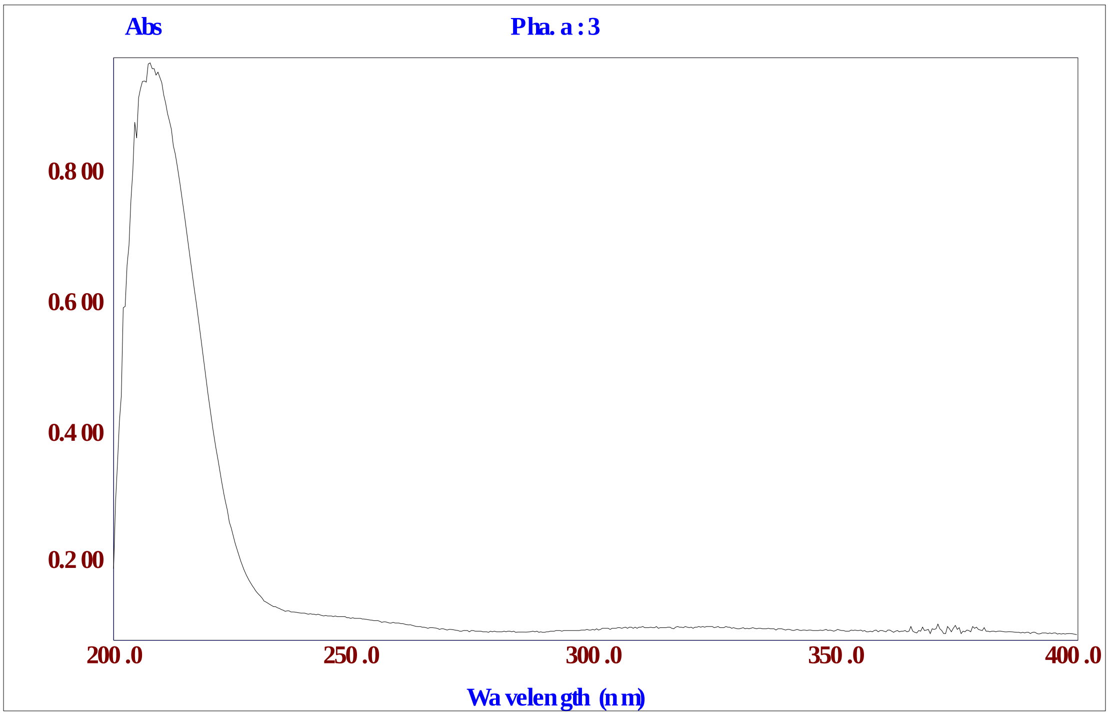

UV of scopararane F (**4**)

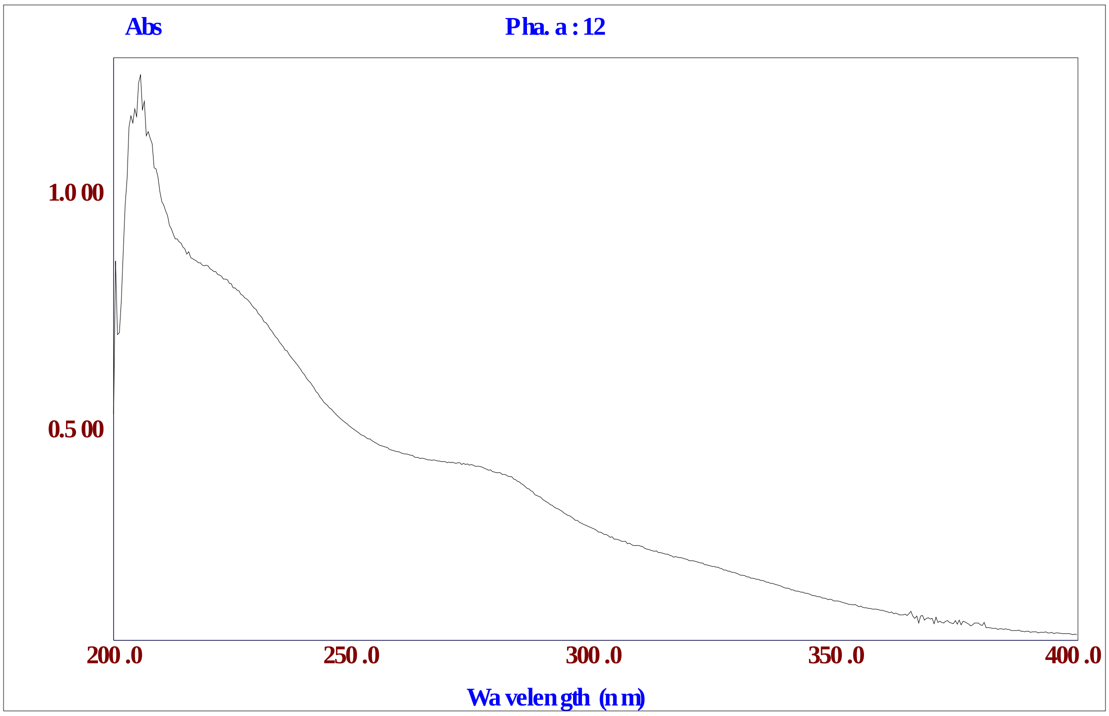

UV of scopararane G (**5**)

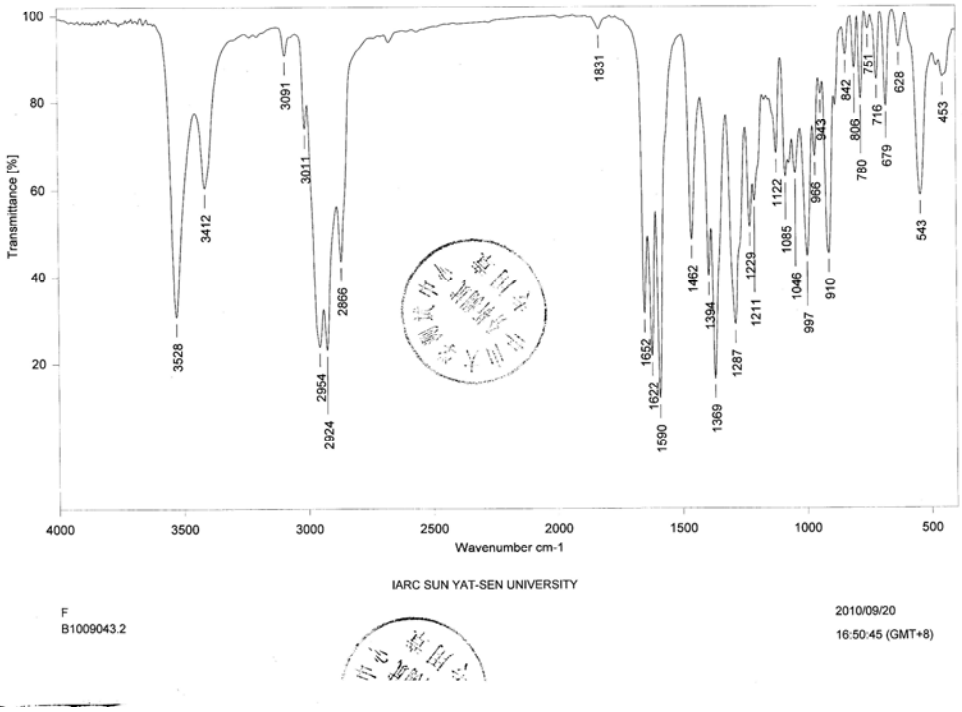

IR of scopararane C

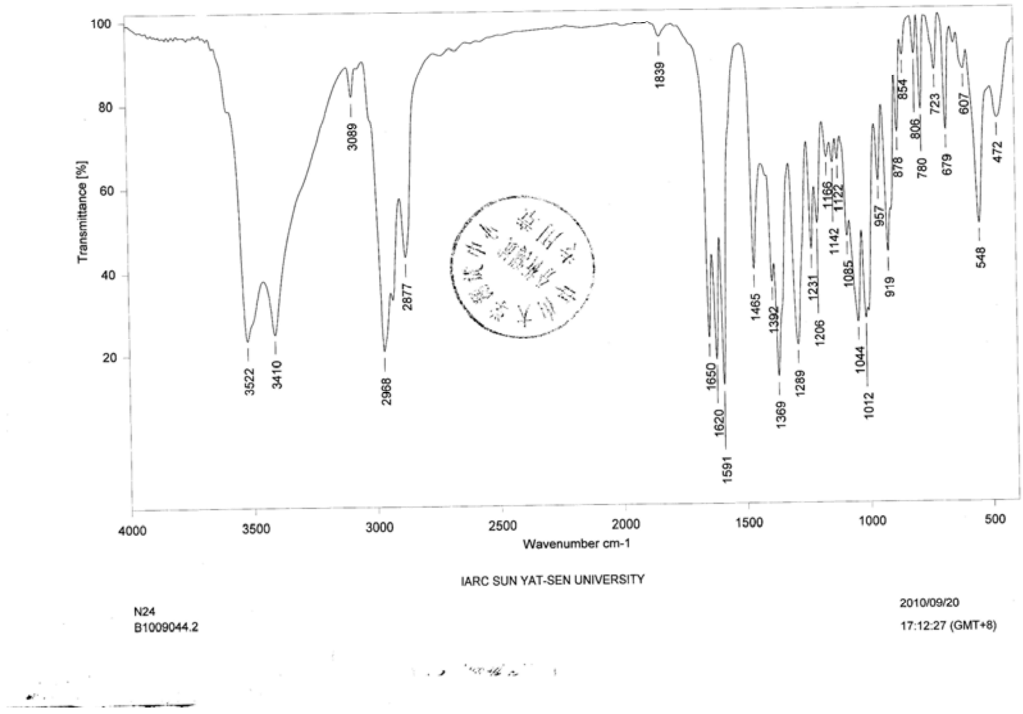

IR of scopararane D

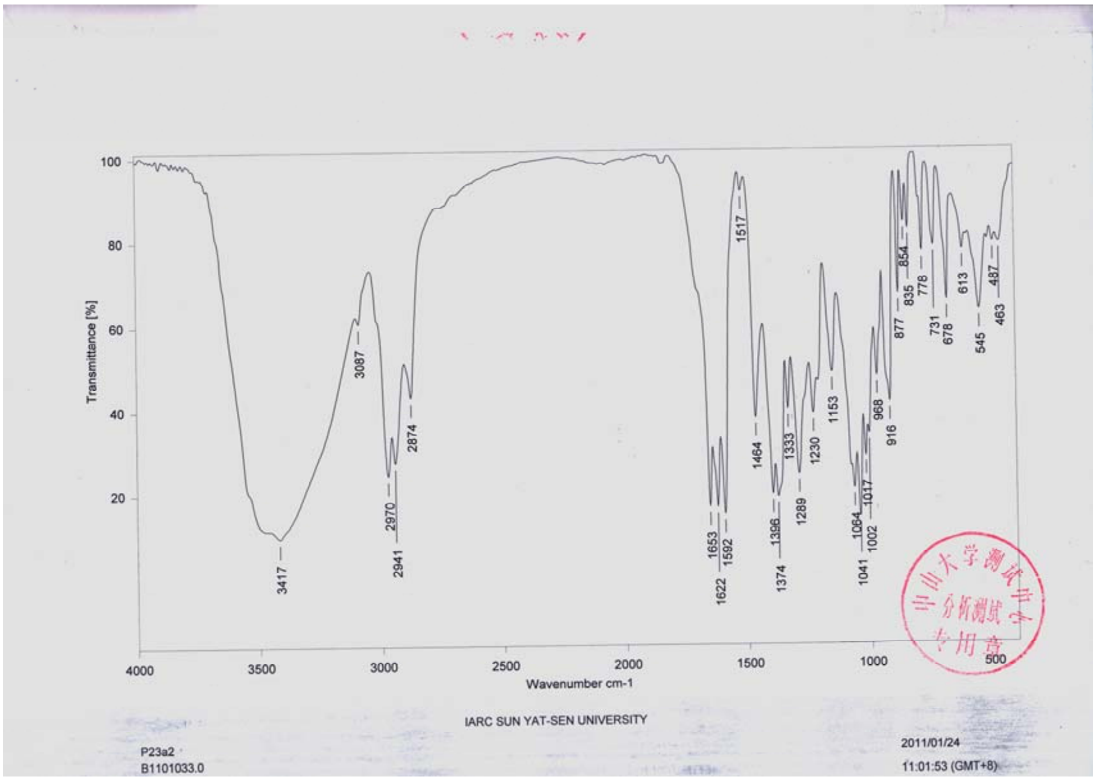

IR of scopararane E

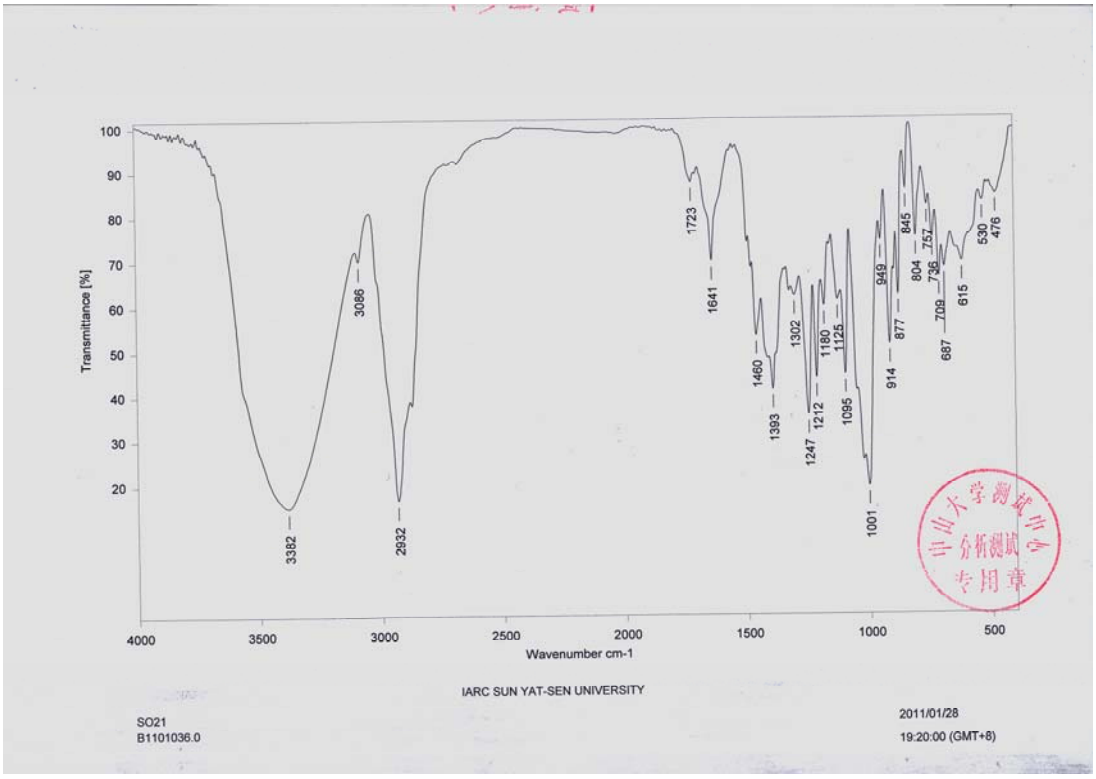

IR of scopararane F

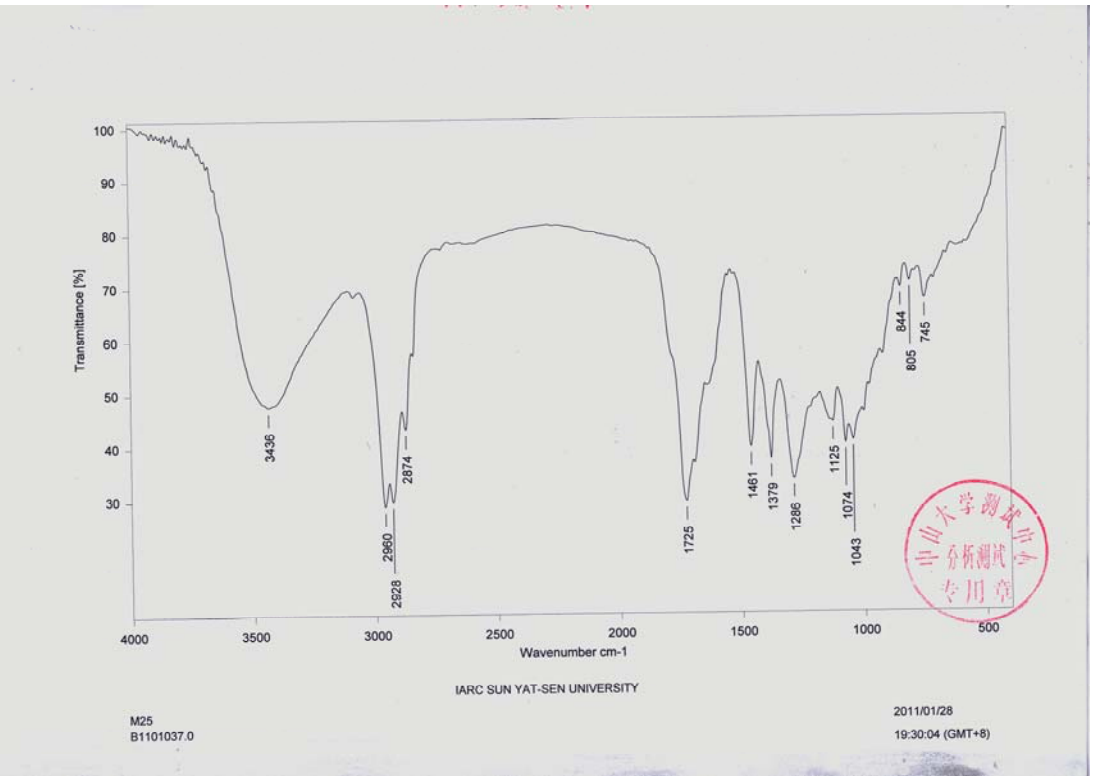

IR of scopararane G

SPECTRUM - MS
File : D:\DATA-HR\10\080701-c1.RAW
Full ms [299.500 - 325.500] - Range: 316.000 - 316.800
Scan No. 3 of 7
Scan #: 3
RT: 0.23
Data points: 1

Mass	Relative Intensity	Theoretical Mass	Delta [ppm]	Delta [mmu]	RDB	Composition
316.2032	92.7	316.2033	-0.2	-0.1	7.0	C_{20} H_{28} O_3

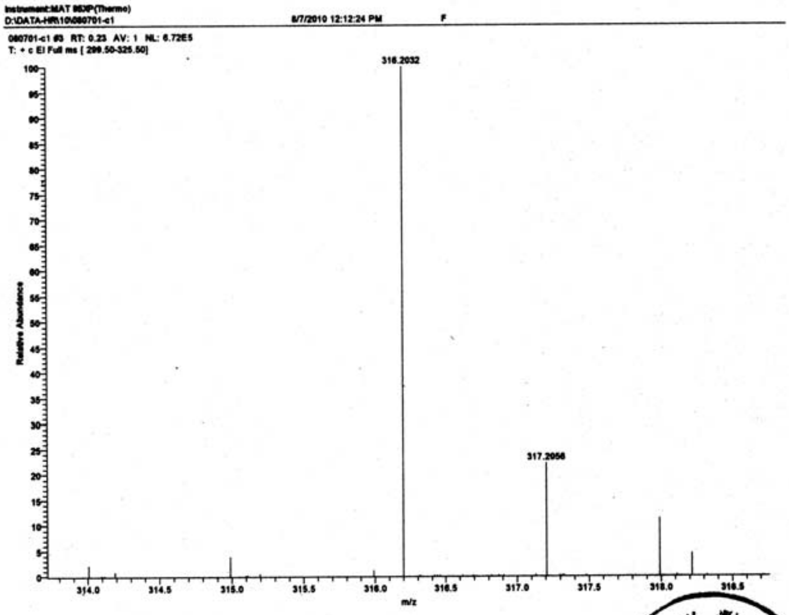

中山大学测试中心 分析测试 专用章

MS of scopararane C

SPECTRUM - MS
File : D:\DATA-HR\10\080702-c1.RAW
Full ms [325.500 - 348.500] - Range: 332.000 - 332.800
Scan No. 10 of 12
Scan #: 10
RT: 0.67
Data points: 1

Mass	Relative Intensity	Theoretical Mass	Delta [ppm]	Delta [mmu]	RDB	Composition
332.1983	79.4	332.1982	0.2	0.1	7.0	C_{20} H_{28} O_4

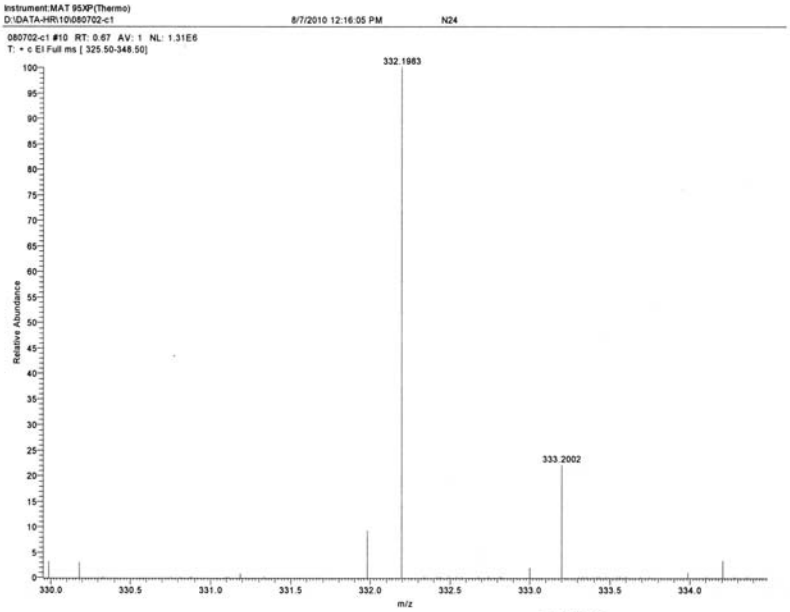

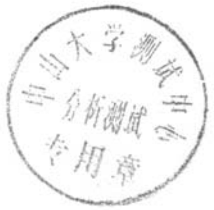

MS of scopararane D

01/01/2006 01:39 02039352523 PAGE 05

Elemental Composition Report

Page 1

Single Mass Analysis

Tolerance = 10.0 PPM / DBE: min = -1.5, max = 50.0
Element prediction: Off
Number of isotope peaks used for i-FIT = 3

Monoisotopic Mass, Even Electron Ions
148 formula(e) evaluated with 3 results within limits (up to 50 best isotopic matches for each mass)
Elements Used:
C: 0-500 H: 0-1000 O: 0-200 Na: 0-1
27-Oct-201010:04:13
P23a2 6 (0.345) AM (Cen,3, 80.00, Ar,5000.0,345.00,0.70,LS 10); Sm (Mn, 2x1.00); Cm (6:32)

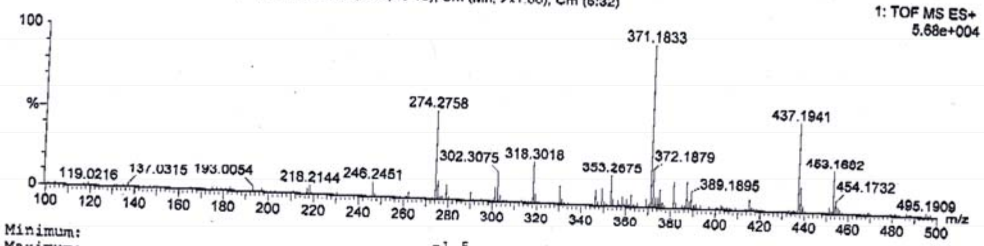

Minimum:				-1.5		
Maximum:		5.0	10.0	50.0		
Mass	Calc. Mass	mDa	PPM	DBE	i-FIT	Formula
371.1833	371.1858	-2.5	6.7	9.5	279.3	C22 H27 O5
348	371.1834	-0.1	-0.3	6.5	318.3	C20 H28 O5 Na ✓
	371.1800	3.3	8.9	18.5	943.1	C29 H23

MS of scopararane E

01/01/2006 01:39 02039352523 PAGE 04

Elemental Composition Report

Page 1

Single Mass Analysis

Tolerance = 10.0 PPM / DBE: min = -1.5, max = 50.0
Element prediction: Off
Number of isotope peaks used for i-FIT = 3

Monoisotopic Mass, Even Electron Ions
142 formula(e) evaluated with 3 results within limits (up to 50 best isotopic matches for each mass)
Elements Used:
C: 0-500 H: 0-1000 O: 0-200 Na: 0-1
27-Oct-201009:55:01
S21 19 (1.224) AM (Cen,3, 80.00, Ar,5000.0,345.00,0.70,LS 10); Sm (Mn, 2x1.00); Cm (5:26)

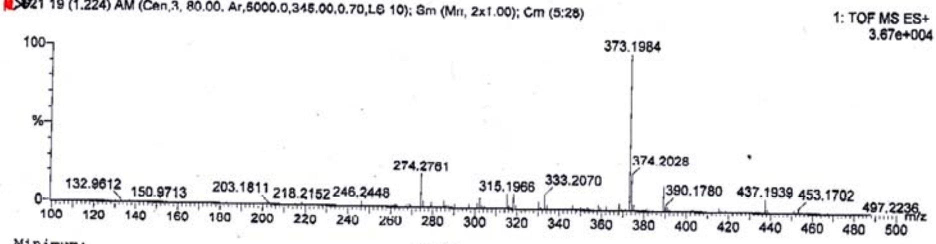

Minimum:				-1.5		
Maximum:		5.0	10.0	50.0		
Mass	Calc. Mass	mDa	PPM	DBE	i-FIT	Formula
373.1984	373.1991	-0.7	-1.9	5.5	19.8	C20 H30 O5 Na ✓
350	373.2015	-3.1	-8.3	8.5	44.3	C22 H29 O5
	373.1956	2.8	7.5	17.5	550.4	C29 H25

MS of scopararane F

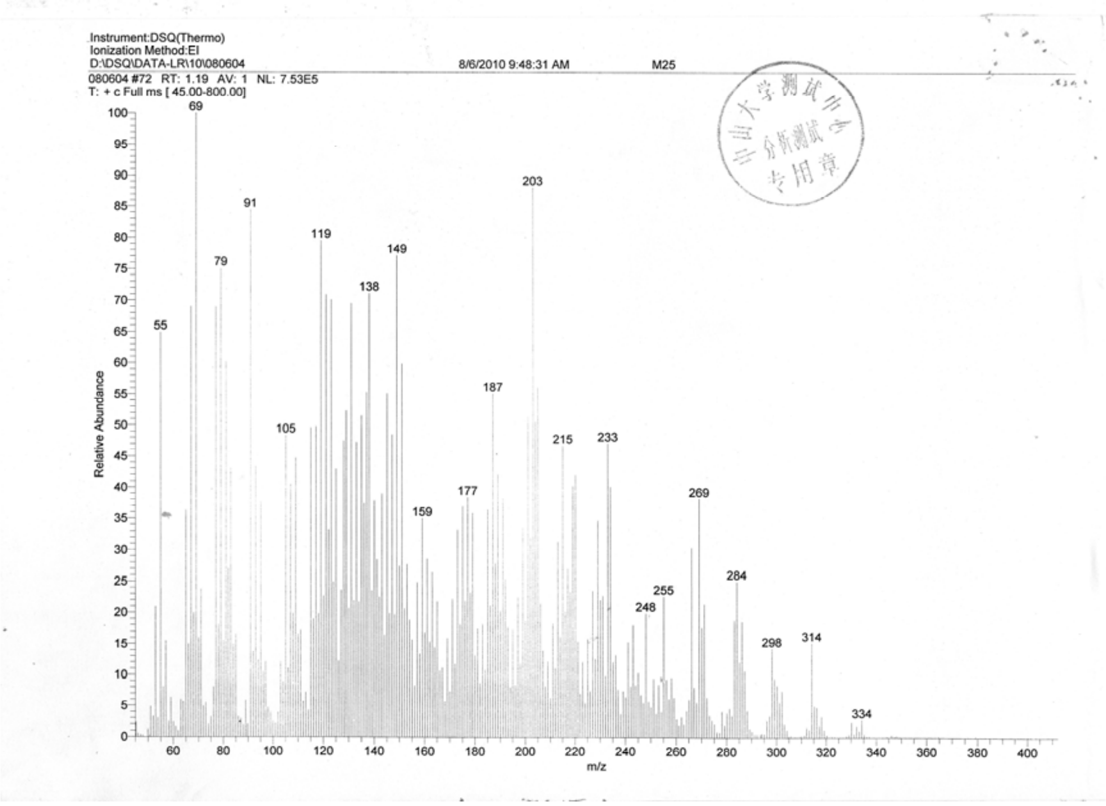

MS of scopararane G
